# Merging Allosteric and Active Site Binding Motifs: De novo Generation of Target Selectivity and Potency via Natural-Product-Derived Fragments

**DOI:** 10.1002/cmdc.201402478

**Published:** 2014-12-08

**Authors:** Jan Lanz, Rainer Riedl

**Affiliations:** [a] Institute for Chemistry and Biological Chemistry, Zurich University of Applied SciencesEinsiedlerstrasse 31, 8820 Wädenswil (Switzerland) E-mail: rainer.riedl@zhaw.ch Homepage:http://www.icbc.zhaw.ch/organic-chemistry

**Keywords:** de novo drug design, enzyme inhibitors, fragment-based drug discovery, natural products, structure–activity relationships, water-mediated interactions

## Abstract

The de novo design of molecules from scratch with tailored biological activity is still the major intellectual challenge in chemical biology and drug discovery. Herein we validate natural-product-derived fragments (NPDFs) as excellent molecular seeds for the targeted de novo discovery of lead structures for the modulation of therapeutically relevant proteins. The application of this de novo approach delivered, in synergy with the combination of allosteric and active site binding motifs, highly selective and ligand-efficient non-zinc-binding (**3**: 4-{[5-(2-{[(3-methoxyphenyl)methyl]carbamoyl}eth-1-yn-1-yl)-2,4-dioxo-1,2,3,4-tetrahydropyrimidin-1-yl]methyl}benzoic acid) as well as zinc-binding (**4**: 4-({5-[2-({[3-(3-carboxypropoxy)phenyl]methyl}carbamoyl)eth-1-yn-1-yl]-2,4-dioxo-1,2,3,4-tetrahydropyrimidin-1-yl}methyl)benzoic acid) uracil-based MMP-13 inhibitors presenting IC_50_ values of 11 nm (**3**: LE=0.35) and 6 nm (**4**: LE=0.31).

Despite the fact that a lot of progress has been made in structure-based drug design, there is still no general approach available for the de novo design of novel drugs. This problem has been addressed by computational tools and by experimental approaches, but it still remains the major challenge for chemists in the search for innovative molecules to modulate biological systems.[1]

Natural products can be considered as biologically validated by evolution and should therefore be ideal starting points for the discovery of bioactive molecules. They have facilitated drug discovery in the past, but due to their chemical complexity their impact on drug discovery relative to synthetic small molecules has decreased over time.[2] Recently, natural products have again drawn increasing attention in drug discovery as a source of molecules that cover a complementary chemical space relative to purely synthetic molecules.[3]

Fragment-based drug discovery has evolved as a new paradigm in the search for small-molecule modulators of therapeutic targets, as it allows the development of ligand-efficient drug molecules by starting with small fragments rather than with complex molecules such as natural products.[4] Very recently, the chemical space targeted by fragments has been extended by the deconstruction of complex natural products in order to provide biocompatible alternatives to synthetic fragments.[5] However, the benefit of natural-product-derived fragments (NPDFs) for the design of high-affinity ligands with selectivity profiles suitable for clinical drug candidates has yet to be proven.

In this study, we set out to establish NPDFs as powerful seeds for the de novo creation of organic molecules with tailored biological activity using matrix metalloproteinase 13 (MMP-13) as an exemplary target. We proved this paradigm by the de novo design, synthesis, and biological evaluation of potent, selective, and ligand-efficient MMP-13 inhibitors based on uracil as the fragment seed (Figure 1).

**Figure 1:**
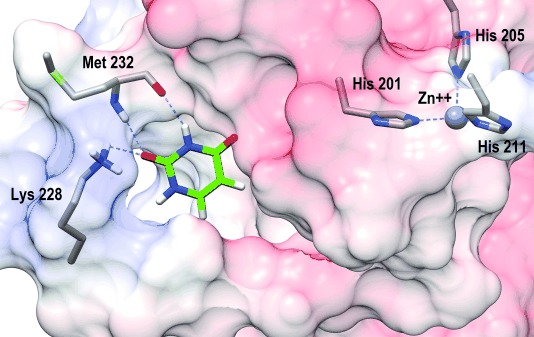
Probing the MMP-13 S1′-binding site by uracil: Uracil addresses the Met232 backbone NH/CO and the side chain amino functionality of Lys228; molecular models generated with MOE;[6] images generated with CHIMERA;[7] (MMP-13 PDB code: 2OW9);[8] C (protein): gray; C (inhibitor scaffold): green; N: blue; O: red.

MMP-13 is a highly relevant and validated target for a multitude of severe diseases such as cancer,[9] osteoarthritis,[10] rheumatoid arthritis,[11] cardiovascular[12] and neurodegenerative diseases.[13] MMP-13 is a member of the zinc-dependent endopeptidase family. It is the dominant MMP involved in type II collagen cleavage in the degradation process of extracellular matrix during growth and tissue remodeling.[14] All clinical candidates containing strong zinc-binding groups have failed in clinical trials.[15] Unsatisfying bioavailability and severe side effects due to a lack of selectivity and the metabolic liability of zinc-binding functionalities such as hydroxamic acids has hampered their development. Hence, the discovery of efficacious MMP inhibitors without strong zinc-binding functional moieties is a very active field of research toward the generation of clinical treatments for those serious conditions.[16]

Peptide bonds of the protein backbone represent the ubiquitous binding motif for small molecules to modulate a biological target. We used this omnipresent H-bonding motif as the starting point for our de novo design concept based on NPDFs. This combines 1) targeting an omnipresent binding motif and 2) the use of biologically validated NPDFs as seeds for de novo drug-design processes. This represents a general de novo design approach by using biocompatible fragments, thus preventing the use of synthetic fragments with debatable biological properties.

First, we selected a NPDF that, based on its molecular architecture, is able to probe the binding site of a biological target for possible H-bond interactions with the protein’s peptide bonds. We chose uracil as NPDF because: 1) this fragment is present in a multitude of very prominent natural products, such as ribonucleic acid (RNA), 2) it offers several synthetic anchor points for the development of a lead structure by organic synthesis, and 3) uracil interacts strongly with biomolecules such as adenine in RNA via H-bonds due to its *cis* amide bonds. Starting with the bare protein of an MMP-13 X-ray crystal structure (PDB code: 2OW9),[8] we probed the selectivity-enabling, non-zinc-binding S1′-binding site of the protein in silico with uracil for preferred H-bonding interactions, and found a remarkable conserved binding motif of the uracil molecule within this allosteric binding site (Figure 1 and Figure S1, Supporting Information (SI)).

Uracil interacts via its *cis* amide bonds with the backbone NH and CO groups of Met232 in the top-ranked docking poses. This binding motif has not been observed in any co-crystal structure of MMP-13 with an inhibitor so far, and therefore offered a unique starting point for the de novo design of novel non-zinc-binding MMP-13 inhibitors. In addition, uracil addresses the side chain amino group of Lys228.

We exploited this distinctive binding motif to elongate the uracil fragment by adding target-oriented functional fragments to strengthen the affinity of the developing inhibitor scaffold: 1) N1-alkylation of the uracil fragment by a benzylic fragment in order to interact with the aromatic side chains of Tyr225 and Phe231 via CH–π interactions, 2) elongation at uracil C5 by a linear propiolic acid fragment to interact with the backbone NH of Thr224 within the linear S1′-binding site, and 3) addition of a benzylic fragment to the propiolic acid terminus to interact with His201 via π–π interaction (Figure 2 and SI Figure S2; further design details are given in the Supporting Information).

**Figure 2:**
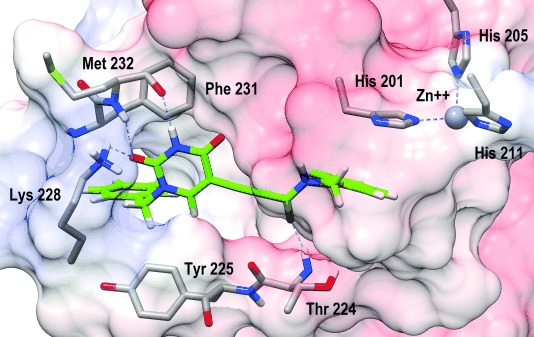
Top-ranked docking pose of the de novo uracil-based MMP-13 inhibitor scaffold 2 within the allosteric binding site.

Subsequent synthesis of the inhibitor scaffold **2** was efficiently carried out by starting with 5-iodouracil (**1**) using palladium-catalyzed cross-coupling chemistry as the central C–C bond formation strategy, which is well suited for introducing various substitution patterns (Scheme 1 and SI Scheme S1).[17]

**Scheme 1:**
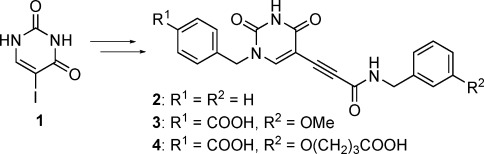
Transformation of 5-iodouracil 1 into protease inhibitors 2–4 (full synthetic details are given in the Supporting Information).

The biological evaluation of scaffold **2** revealed a very promising initial affinity against MMP-13 and a superb selectivity profile against a variety of other members of the MMP family, as intended by our de novo design (Table 1).

**Table 1:** Biological MMP inhibitory potency and selectivity profile

Inhibitor	MMP-13^[a]^	MMP-1, -2, -3, -7, -8, -9, -12, -14^[b]^
**2**	5.13 μm [pIC_50_=5.29±0.17]	<50 % inhibition at 20 μm
**3**	10.90 nm [pIC_50_=7.96±0.05]	<50 % inhibition at 20 μm
**4**	5.78 nm [pIC_50_=8.24±0.15]	<50 % inhibition at 10 μm

[a] Data represent the mean ±SD of one experiment performed in triplicate. [b] Single-dose inhibition data are from one experiment performed in triplicate; confidence intervals, detailed single-dose inhibition data, and full experimental details for the biological assays are given in the Supporting Information.

We calculated the Tanimoto coefficients (JChem for Excel, FP2, ChemAxon)[18] for scaffold **2** in comparison with all currently known MMP-13 inhibitors available through the ChEMBL data base (http://www.ebi.ac.uk/chembl/, ChEMBL-ID: CHEMBL280, 2580 compounds) in order to evaluate the fingerprint-based novelty of the uracil scaffold **2**. The highest Tanimoto similarity index was 0.43. This clearly indicates that based on the NPDF uracil as molecular seed and our structure-based de novo design approach we furnished a novel chemotype for MMP-13 inhibitors.[19]

To understand the binding motif of the inhibitor scaffold, we performed systematic synthetic modifications on **2**. The introduction of *o*-F substitution on the aromatic ring of the benzylamino fragment resulted in a drastic loss of binding affinity, whereas *m*-F and *p*-F substitutions were tolerated (SI Table S1). This can be rationalized by the proposed binding motif and the consequential negative interactions of the *o*-F substituent with the backbone CO group of Phe220 (detailed SAR analysis is given in the Supporting Information, Figure S3).

To improve the initial inhibitor scaffold **2** to a lead-like binding affinity and selectivity profile, water-mediated interactions between the inhibitor and the target protein were targeted.[16a] By introducing a carboxylic acid at the 4-position of the aromatic system on the left-hand side of the inhibitor scaffold to address structural water HOH822 and a methoxy group at the 3-position of the aromatic system on the right-hand side of the inhibitor scaffold to address water molecules HOH836 and HOH915 (SI Figure S4), the potency could be improved substantially (**3**: IC_50_=11 nm) while retaining the superb selectivity profile (Table 1).

Furthermore, we elongated the methoxy group of inhibitor **3** with a propanoic acid fragment in order to verify the proposed binding motif conclusively by merging the two binding motifs of non-zinc-binding and zinc-binding MMP inhibitors. This concept of combining those two motifs (S1′ non-zinc-binding selectivity elements plus a zinc-binding group) has been proposed in the past but has not yet been realized.[20] As expected, inhibitor **4** indeed showed further improved binding affinity (IC_50_=6 nm) and still retained an extraordinary selectivity profile (Table 1). Thus, to our knowledge, inhibitor **4** is the first example of this type of combined binding motif.

Finally, an overlay of the X-ray crystal structure of the non-zinc-binding MMP-13 inhibitor from PDB 2OW9 with the docked inhibitor **4** supports the proposed binding motif and suggests that the combination of allosteric and active site binding elements is a widely applicable approach toward highly potent and selective therapeutic agents (Figure 3).

**Figure 3:**
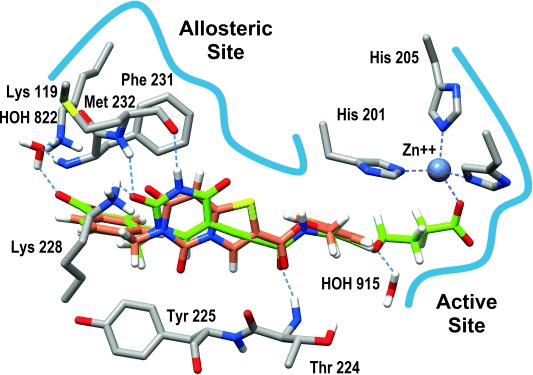
Overlay of PDB 2OW9 and 4: Combination of allosteric and active site binding motifs; C (protein): gray; C (inhibitor in PDB 2OW9): coral; C (4): green; N: blue; O: red; S: yellow.

In conclusion, we have demonstrated that structure-based de novo design from scratch based on NPDFs is a powerful technique for the efficient discovery of potent and selective lead molecules for modulating therapeutically relevant targets. In addition, by combining allosteric and active site binding motifs, the presented approach delivered novel chemotypes of zinc-binding as well as non-zinc-binding MMP-13 inhibitors. This structured approach holds great promise for successful application against other therapeutic targets as well. Finally, our study validates the benefits of fragment-based techniques in combination with natural products, which will reinforce the significance of natural products for future drug-discovery efforts.

## Experimental Section

**Compound 4**: Iodouracil derivative **5 b** (550 mg, 1.48 mmol, 1.0 equiv) and alkyne derivative **8 g** (405 mg, 1.55 mmol, 1.05 equiv) were dissolved in anhydrous DMF (8 mL) under N_2_ atmosphere. Pd(PPh_3_)_4_ (171 mg, 0.15 mmol, 0.1 equiv), CuI (56 mg, 0.30 mmol, 0.2 equiv) and Et_3_N (2 mL) were added at 20 °C. The resulting mixture was stirred for 4 h at 20 °C. The reaction mixture was quenched by adding H_2_O. The resulting suspension was acidified with 2 m HCl. The precipitate was filtered off, washed with H_2_O, suspended in MeOH and cooled to −20 °C. The precipitate was filtered off and washed with cold MeOH. The crude product was purified by reversed-phase flash chromatography and recrystallization from MeOH/H_2_O (10:1). Yield: 295 mg, 0.58 mmol, 39 %, white solid; purity >99 % (HPLC); dp: 224 °C; ^1^H NMR (700 MHz, [D_6_]DMSO): *δ*=12.55 (s, 2 H), 11.84 (s, 1 H), 9.16 (t, *J*=6.2 Hz, 1 H), 8.46 (s, 1 H), 7.93 (d, *J*=7.9 Hz, 2 H), 7.44 (d, *J*=7.6 Hz, 2 H), 7.25–7.20 (m, 1 H), 6.83–6.79 (m, 3 H), 4.99 (s, 2 H), 4.27 (d, *J*=6.2 Hz, 2 H), 3.95 (t, *J*=6.2 Hz, 2 H), 2.39 (t, *J*=7.8 Hz, 2 H), 1.93 ppm (ps. quin, *J*=6.8 Hz, 2 H); ^13^C NMR (176 MHz, [D_6_]DMSO): *δ*=174.06, 166.99, 161.60, 158.56, 152.21, 151.65, 149.90, 141.10, 140.30, 130.22, 129.70 (2 C), 129.43, 127.59 (2 C), 119.48, 113.59, 112.77, 95.64, 86.94, 77.52, 66.48, 50.92, 42.30, 30.16, 24.32 ppm; ESI-TOF-HRMS: *m*/*z* 506.1549 [*M*+H]^+^, calcd for C_26_H_23_N_3_O_8_: 505.1485, found: 505.1475; Anal. calcd for C_26_H_23_N_3_O_8_ [%]: C 61.78, H 4.59, N 8.31, found [%]: C 61.55, H 4.54, N 8.24.

## Supporting Information

As a service to our authors and readers, this journal provides supporting information supplied by the authors. Such materials are peer reviewed and may be re-organized for online delivery, but are not copy-edited or typeset. Technical support issues arising from supporting information (other than missing files) should be addressed to the authors.

miscellaneous_information
