# Supplementary material for: Merging Allosteric and Active Site Binding Motifs: De novo Generation of Target Selectivity and Potency via Natural-Product-Derived Fragments
Source: ChemMedChem. 2014 Dec 8;10(3):451–4. doi: 10.1002/cmdc.201402478 (PMC4506557; doi:10.1002/cmdc.201402478)
Supplement: Supplementary file 1 — miscellaneous_information [file cmdc0010-0451-sd1.pdf]

CHEMMEDCHEM
Chemistry & Drug Discovery

Supporting Information

© Copyright Wiley-VCH Verlag GmbH & Co. KGaA, 69451 Weinheim, 2015

Merging Allosteric and Active Site Binding Motifs: De novo Generation of Target Selectivity and Potency via Natural-Product-Derived Fragments

Jan Lanz and Rainer Riedl*[a]

cmdc_201402478_sm_miscellaneous_information.pdf

Supporting Information

Table of Contents

Molecular Modeling S2

Structure Activity Relationship (SAR) S4

General Experimental Conditions S7

Synthetic Procedures S8

- General procedure for amid coupling S11
- General procedure for Sonogashira cross-coupling S15

Biological Assays S20

Analytical Data S24

- NMR spectra S24
- ESI-TOF-HRMS S62
- IR spectra S70
- HPLC – Purity S78
- Elemental analysis S81

References S83

Molecular Modeling

All molecular modeling experiments were performed using the Molecular Operating Environment (MOE) software (Versions: 2012.10 and 2013.08) from Chemical Computing Group (http://www.chemcomp.com).[1] The co-crystal structure 2OW9[2] was obtained as PDB-file from the RCSB Protein Data Bank (http://www.rcsb.org),[3] loaded to MOE, reduced to a monomer and prepared by calculating the protonation using the *Protonate 3D* application with default settings (Temperature = 300 K, pH = 7, Salt = 0.1). New ligands were loaded to MOE as 2D structures from SD-files. The 3D coordinates were calculated using the *Rebuild3D* application with default settings (RMSD gradient = 0.1). Even though the calculation of the 3D coordinates produces a local minimum of the molecular energy of the ligands, a further minimization was performed using the energy minimize application. The docking experiments were carried out applying the MMFF94x force field and the triangle matcher placement. All ligands were docked against the co-crystal structure (incl. water molecules; without the water molecules 747, 836 and 915 for docking experiments of compound **4**).

Probing the MMP-13 S1'-binding site by uracil

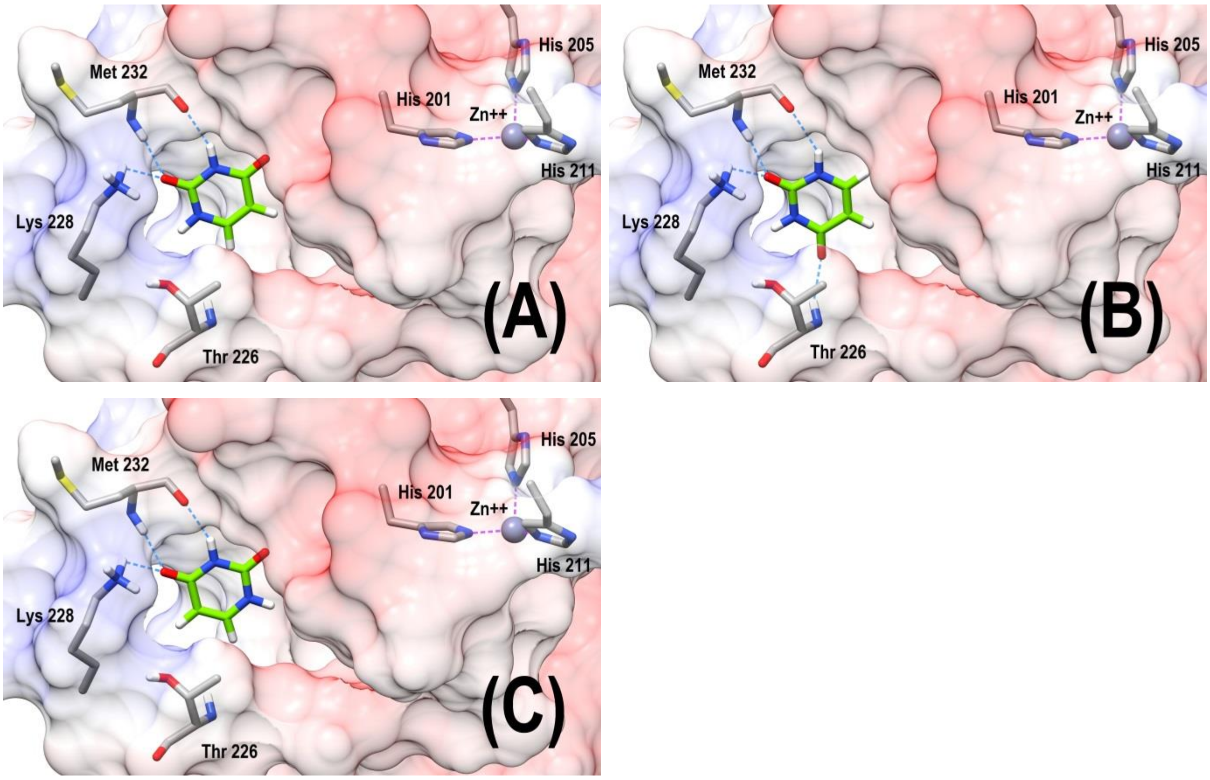

Figure S1: Top-ranked poses of uracil (A, B, C) showing the binding motif which addresses the Met232 backbone NH/CO and the side chain amino functionality of Lys228; color code: C (protein): gray; C (inhibitor scaffold): green; N: blue; O: red. Pose A was used for the *de novo* design approach. Molecular models were generated with MOE; Images were generated with CHIMERA;[4] (MMP-13 PDB code: 2OW9).

De novo design approach from uracil to scaffold 2

Uracil as NPDF interacts via its *cis* amide bonds with the backbone NH and CO of Met232, as well as the side chain amino group of Lys228 in the top ranked poses.

After a thorough investigation of the binding site for possible binding partners and the vectors provided by the uracil fragment for synthetic modifications, we introduced a benzylic group by N1-alkylation of the uracil in order to interact with the aromatic side chains of Tyr225 and Phe231 via CH-pi interactions. The top-ranked pose for this elongation shows the unique H-bonding pattern to Met232 as well as the intended CH-pi interactions in a sandwich-type binding orientation.

Consequently, we aimed to bind deeper into the S1'-binding site of the target protein to further improve the affinity of the emerging inhibitor. C5 of the uracil fragment offered an attractive vector to take advantage of the linear S1'-binding site by the introduction of a linear propiolic acid fragment addressing the backbone NH of Thr224 via the carboxylic acid of the emerging inhibitor.

Adding a benzyl amine to the carboxylic acid terminus in order to interact with His201 via pi-pi-interaction, furnished the final scaffold of our design approach. Again, the top-ranked poses confirmed all the intended interactions between the de novo designed inhibitor scaffold and the target protein.

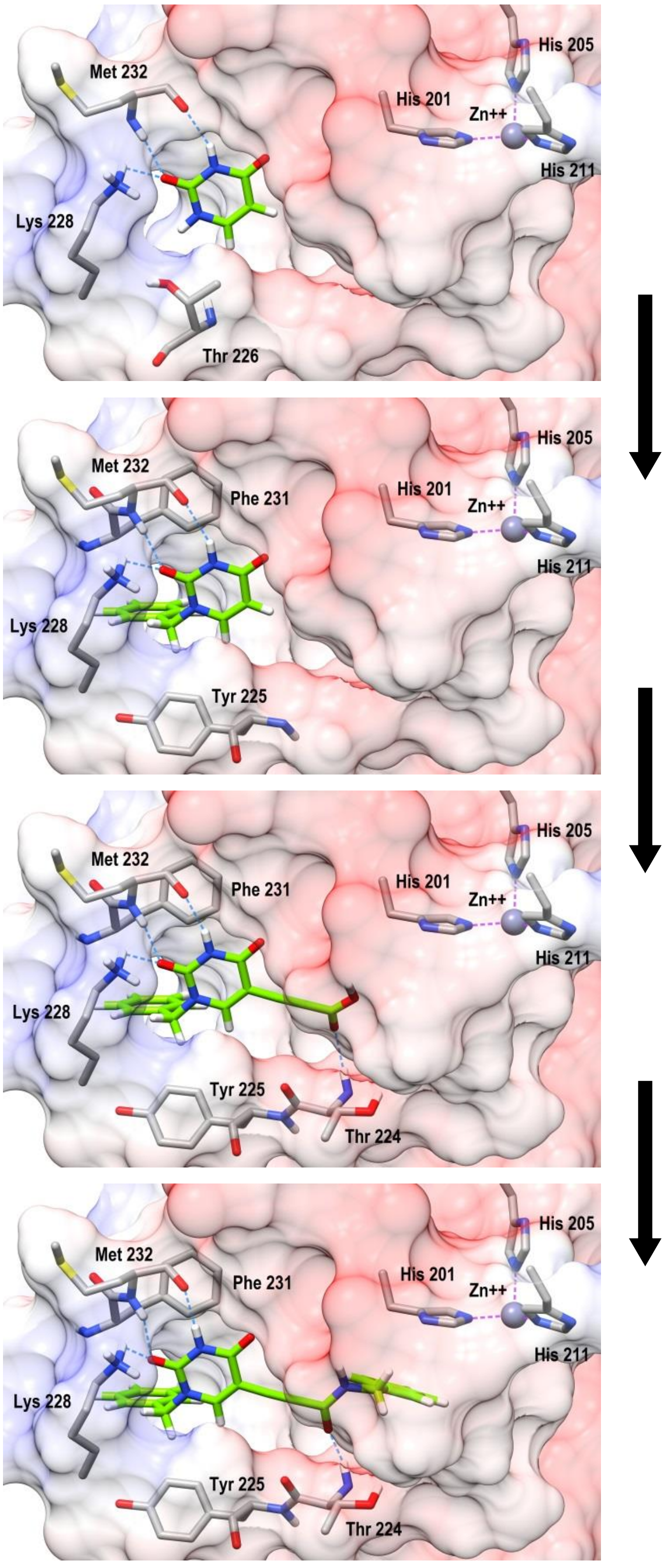

Figure S2: *De novo* design approach from uracil to scaffold **2**; color code: C (protein): gray; C (inhibitor scaffold): green; N: blue; O: red. Molecular models were generated with MOE; images were generated with CHIMERA; (MMP-13 PDB code: 2OW9).

Structure Activity Relationship (SAR)

A directed compound library was designed, synthesized, and tested *in vitro* against MMP-13 in order to verify the docked binding motif of the scaffold **2** (not all data shown). The top ranked poses in the docking experiments of all library compounds showed the same binding motif as the scaffold **2**. Though, the inhibitory potency of all compounds containing a substituent in the *ortho*-position on the benzyl group on the right hand side of the scaffold was eliminated completely at c(inhibitor) = 6.5 µM. These findings correlate consistently regarding the narrowness of the S1'-binding site close to the active site and the clashes that occurred between the docked poses of the library compounds and MMP-13, shown using the example of the three representative library compounds **9a-c**. (Table S1, Figure S3).

Table S1: MMP-13 single dose inhibition data.

Compound	Structure	Inhibition[a]
**2**	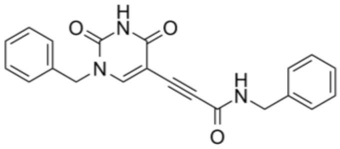	32 ± 9
**3**	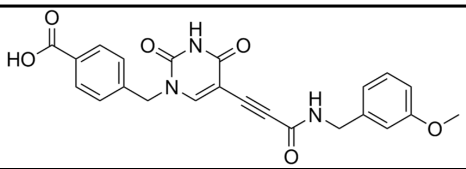	100 ± 1
**9a**	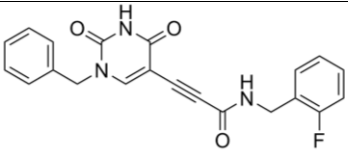	-2 ± 10
**9b**	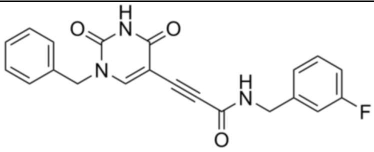	32 ± 4
**9c**	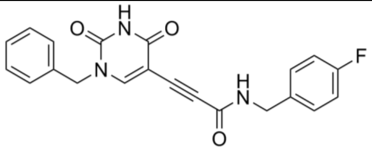	51 ± 2

[a] Single dose values in % ± SD from one experiment measured in-house in triplicate at *c*(inhibitor) = 6.5 µM.

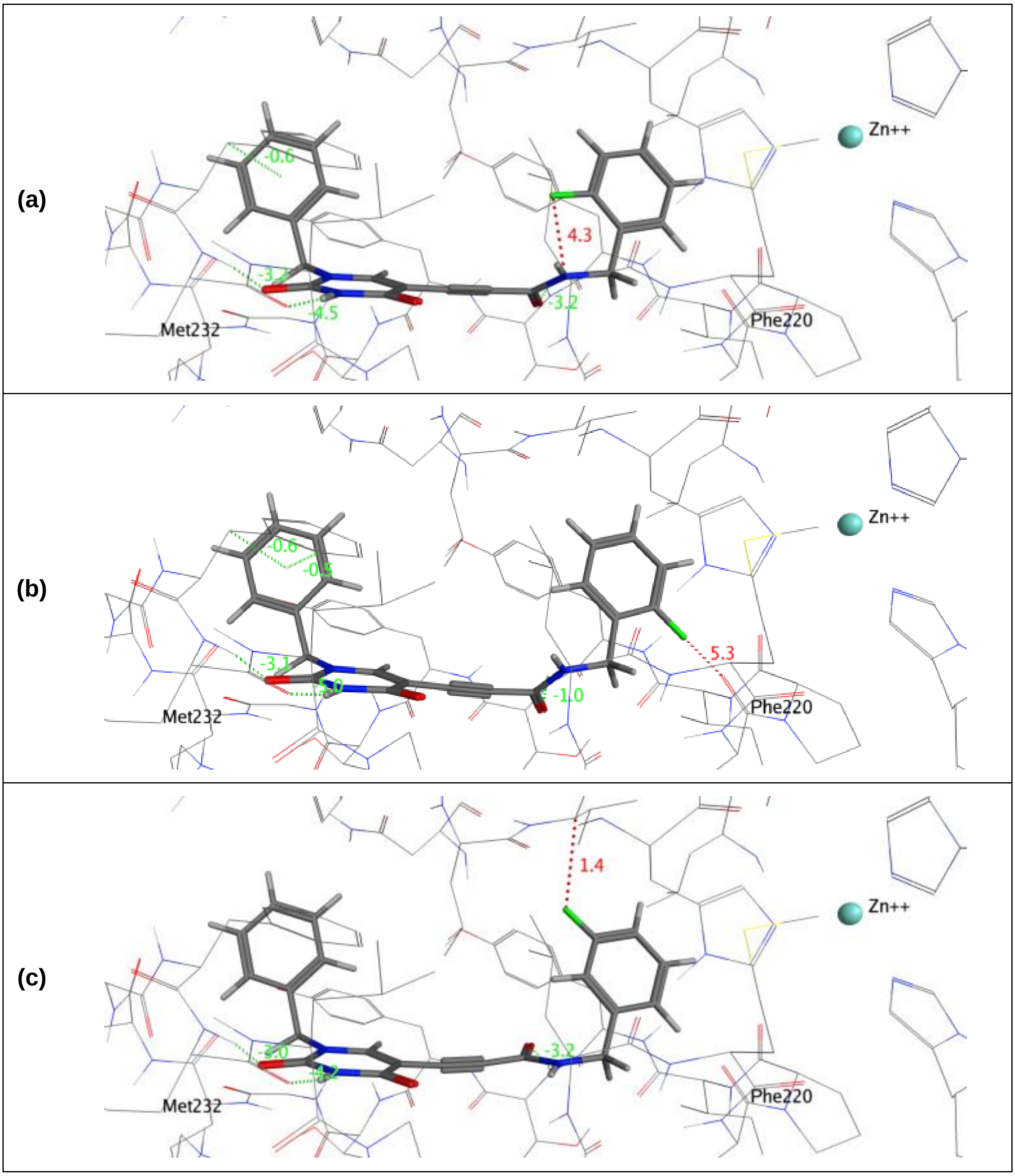

Figure S3: Top-ranked poses of **9a-c** showing the same binding motif as the scaffold **2**; Two possible conformations for **9a**: (a) Intramolecular clash between the fluoro atom and the amid group, (b) intermolecular clash between the fluoro atom and the backbone CO from Phe220; Two possible conformations for **9b**: (c) Weak clash between the fluoro atom and the backbone of Val198; (d) No interaction or clash of the fluoro atom; **9c**: (e) No interaction or clash of the fluoro atom; Color code: C (protein): gray; C (inhibitor): gray; N: blue; O: red. Molecular models and images were generated with MOE; Binding energies (green) and clashes (red) were calculated with MOE, values in kcal/mol; (MMP-13 PDB code: 2OW9).

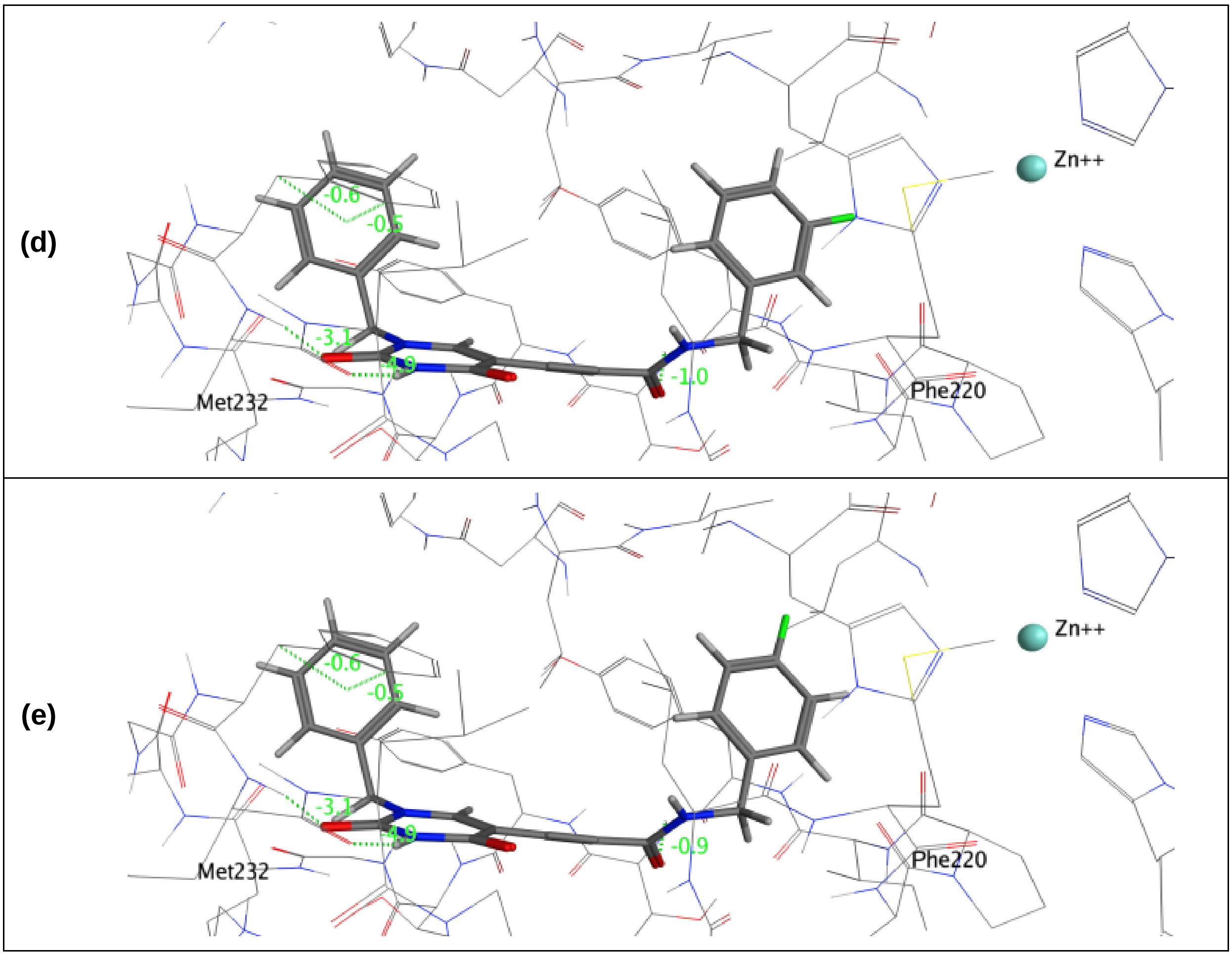

Figure S3 (continued): Top-ranked poses of **9a-c** showing the same binding motif as the scaffold **2**; Two possible conformations for **9a**: (a) Intramolecular clash between the fluoro atom and the amid group, (b) intermolecular clash between the fluoro atom and the backbone CO from Phe220; Two possible conformations for **9b**: (c) Weak clash between the fluoro atom and the backbone of Val198; (d) No interaction or clash of the fluoro atom; **9c**: (e) No interaction or clash of the fluoro atom; Color code: C (protein): gray; C (inhibitor): gray; N: blue; O: red. Molecular models and images were generated with MOE; Binding energies (green) and clashes (red) were calculated with MOE, values in kcal/mol; (MMP-13 PDB code: 2OW9).

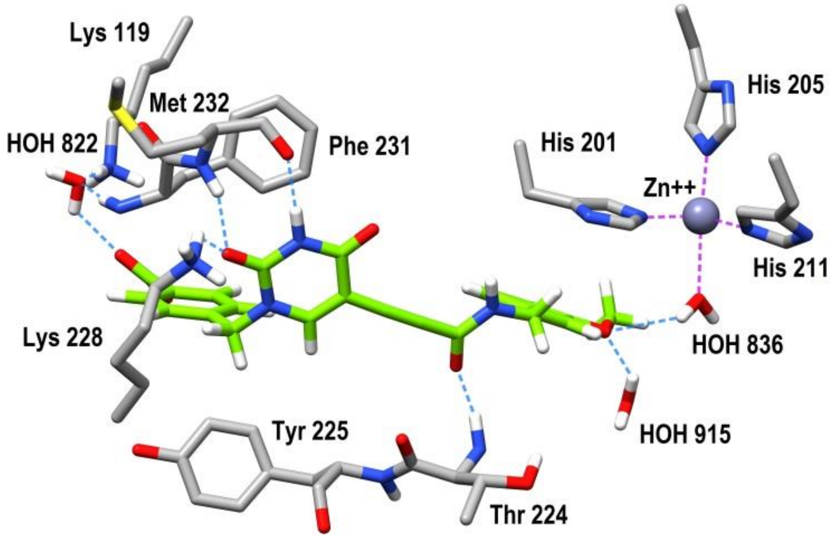

Figure S4: Inhibitor **3** targeting water-mediated interactions; Color code: C (protein): gray; C (inhibitor scaffold): green; N: blue; O: red. Molecular models were generated with MOE; images were generated with CHIMERA; (MMP-13 PDB code: 2OW9).

General Experimental Conditions

Reagents and solvents were purchased from commercial suppliers and used without further purification. Solvents for water free reactions were stored over molecular sieves 4 Å. All microwave assisted reactions were carried out with a Biotage Initiator. Normal-phase (solid phase: silica gel, liquid phase: cyclohexane/ethyl acetate) and reversed-phase (solid phase: C18-reversed phase silica gel, liquid phase: H_2O/MeOH) column chromatography were performed on a Teledyne ISCO CombiFlash Rf system equipped with RediSep Rf columns. NMR spectra were recorded on a Bruker Avance 800 NMR spectrometer (800 MHz for ^{1}H NMR, 201 MHz for ^{13}C NMR), on a Bruker Avance 700 NMR spectrometer (700 MHz for ^{1}H NMR, 176 MHz for ^{13}C NMR) or on a Bruker Avance 300 NMR spectrometer (300 MHz for ^{1}H NMR, 75 MHz for ^{13}C NMR) with chemical shifts reported in ppm relative to the residual solvent peak (DMSO-d6, ^{1}H NMR δ = 2.50 ppm; ^{13}C NMR δ = 39.52 ppm). ^{1}H and ^{13}C NMR data are reported as follows: Chemical shift, multiplicity (s = singlet, d = doublet, t = triplet, quin = quintet, m = multiplet, ps. = pseudo), coupling constants (Hz) and integration. High-resolution mass spectrometry was performed on an Agilent Technologies 6530 Q-TOF. Infrared spectra were recorded on a Bruker Tensor 37 device equipped with an ATR Golden Gate measuring unit. Melting points were measured on a BUCHI Melting Point M-565. The purity of the compounds was determined by HPLC using an Agilent 1200 Series system equipped with an Interchim Uptisphere Strategy C18-2, 5μm, 4.6 x 250mm HPLC column and diode array detector. A standard method was used with conditions as follows: Eluent A: H_2O/MeOH (95:5, v/v) + 0.2 % acetic acid and eluent B: H_2O/MeOH (5:95, v/v) + 0.2 % acetic acid (Table S2); Column temperature ϑ = 40 °C.

Table S2: HPLC Gradient

Time [min]	Flow [mL/min]	Eluent A [%]	Eluent B [%]
0.0	1	100	0
10.0	1	0	100
18.0	1	0	100
18.1	1	100	0
20.0	1	100	0

Synthetic Procedures

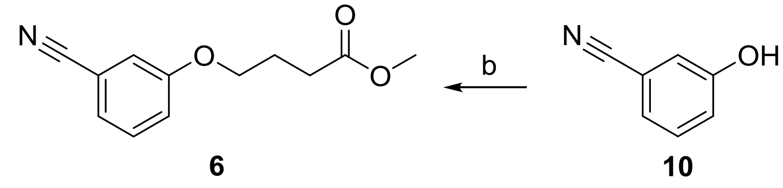
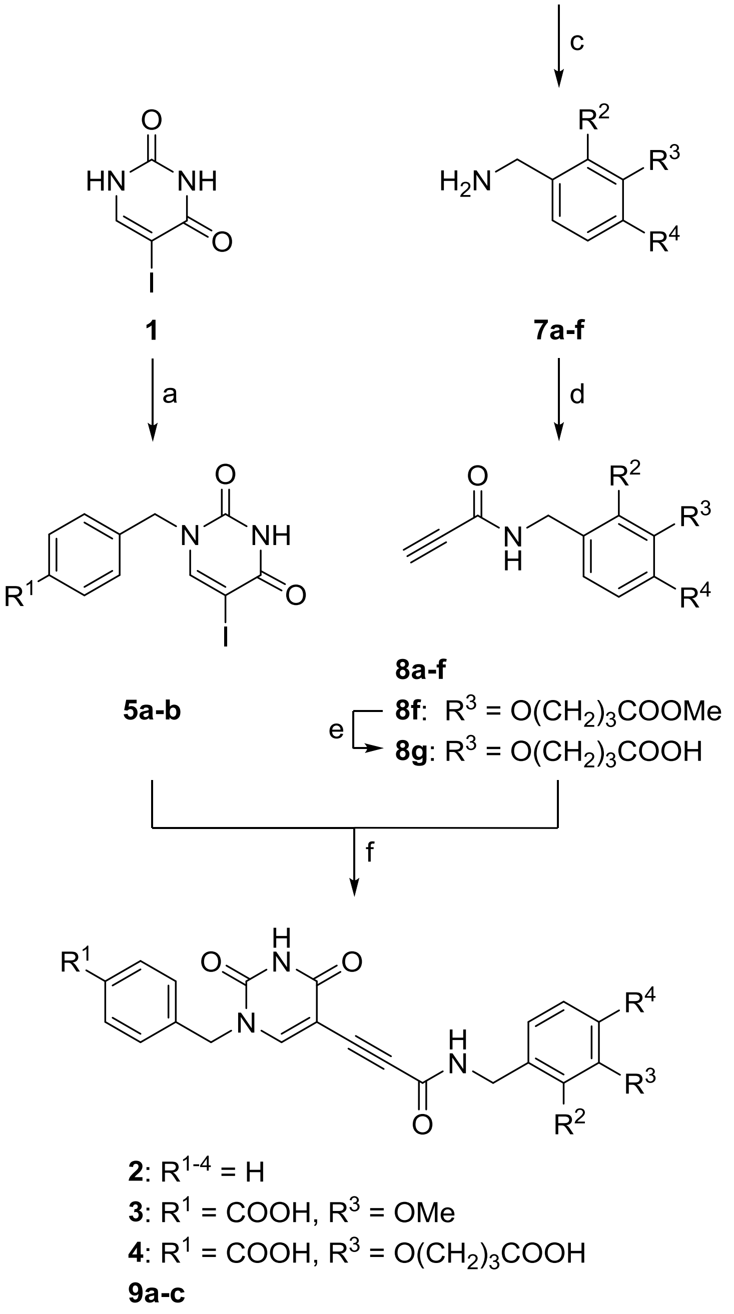

#	R^1	R^2	R^3	R^4
**2**	H	H	H	H
**3**	COOH	H	OMe	H
**4**	COOH	H	$O(CH_2)_3COOH$	H
**5a**	H	-	-	-
**5b**	COOH	-	-	-
**7a**	-	H	H	H
**7b**	-	F	H	H
**7c**	-	H	F	H
**7d**	-	H	H	F
**7e**	-	H	OMe	H
**7f**	-	H	$O(CH_2)_3COOMe$	H
**8a**	-	H	H	H
**8b**	-	F	H	H
**8c**	-	H	F	H
**8d**	-	H	H	F
**8e**	-	H	OMe	H
**8f**	-	H	$O(CH_2)_3COOMe$	H
**8g**	-	H	$O(CH_2)_3COOH$	H
**9a**	H	F	H	H
**9b**	H	H	F	H
**9c**	H	H	H	F

Scheme S1. Synthesis of the *de novo* uracil-based inhibitors. Reagents and conditions: a) BnBr / 4-(Bromomethyl)benzoic acid (1.0 eq.), Cs_2CO_3 (1.05 eq.), DMF, rt, 4 h; b) Methyl 4-bromobutyrate (1.1 eq.), K_2CO_3 (1.5 eq.), DMF, 100 °C, 2 h; c) Pd/C 10% (0.05 eq.), H_2, HCl 32 %, MeOH, rt, 2 h; d) Propiolic acid (1.5 eq.), EEDQ (1.5 eq.), CH_2Cl_2, N_2, rt, overnight; e) LiOH (2 eq.), THF/H_2O (4:1), rt, 3 h; f) $Pd(PPh_3)_4$ (0.1 eq.), CuI (0.2 eq.), TEA, DMF, N_2, rt, 2 h.

1-Benzyl-5-iodo-1,2,3,4-tetrahydropyrimidine-2,4-dione[5] (5a)

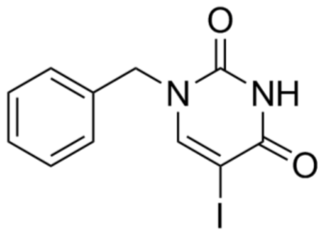

Anhydrous cesium carbonate (6.55 g, 20.3 mmol, 1.05 eq.) was added to a solution of 5-iodouracil (**1**) (4.60 g, 19.3 mmol, 1.00 eq.) in anhydrous DMF (70 mL). The suspension was stirred vigorously at ambient temperature for 1 h. A solution of a benzyl bromide (3.24 g, 19.3 mmol, 1.00 eq.) in anhydrous DMF (10 mL) was added dropwise and the mixture was stirred for another 3 h at ambient temperature. Water (200 mL) was added and the solution was acidified with 2 M HCl. The mixture was extracted with ethyl acetate. The combined organic phase was washed with water and brine, dried over Na_2SO_4 and concentrated under reduced pressure. The crude product was purified by reversed-phase flash chromatography.
Yield: 1.76 g, 5.36 mmol, 28 %, colorless square plates; Purity: >99 %; mp 217-219 °C; ^{1}H NMR (300 MHz, DMSO-d6): δ = 11.67 (s, 1H), 8.32 (s, 1H), 7.40-7.28 (m, 5H), 4.88 (s, 2H); ^{13}C NMR (75 MHz, DMSO-d6): δ = 160.91, 150.67, 149.67, 136.60, 128.58 (2C), 127.67, 127.45 (2C), 68.54, 50.44; IR (ATR): ṽ = 3147, 3112, 3076, 2992, 2824, 1707, 1647, 1608, 1451, 1421, 1338, 882, 729, 691 cm^{-1}; HPLC: t_R 12.07 min; ESI-TOF-HRMS: *m/z* 328.9777 $[M + H]^+$, calculated for $C_{11}H_9IN_2O_2$ 327.9709, found: 327.9705.

4-[(5-Iodo-2,4-dioxo-1,2,3,4-tetrahydropyrimidin-1-yl)methyl]benzoic acid (5b)

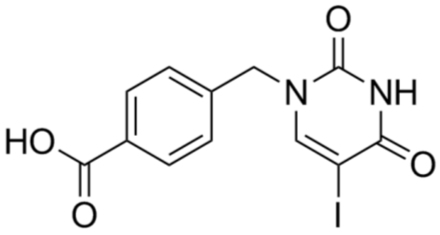

Anhydrous cesium carbonate (2.85 g, 8.82 mmol, 1.05 eq.) was added to a solution of 5-iodouracil (**1**) (10.0 g, 42.02 mmol, 5.0 eq.) in anhydrous DMF (200 mL). The suspension was stirred vigorously at ambient temperature for 1 h. A solution of a 4-(bromomethyl)benzoic acid (1.75 g, 8.40 mol, 1.00 eq.) in anhydrous DMF (50 mL) was added dropwise and the mixture was stirred for another 3 h at ambient temperature. Water (200 mL) was added and the solution was acidified with 2 M HCl. The mixture was extracted

with ethyl acetate. The combined organic phase was washed with water and brine, dried over Na_2SO_4 and concentrated under reduced pressure. The crude product was purified by reversed-phase flash chromatography.

Yield: 0.63 g, 1.70 mmol, 20 %, colorless crystals; Purity: >99 %; dp 300 °C; ^{1}H NMR (300 MHz, DMSO-d6): δ = 12.96 (s, 1H), 11.73 (s, 1H), 8.36 (s, 1H), 7.92 (d, J = 8.3 Hz, 2H), 7.40 (d, J = 8.3 Hz, 2H), 4.95 (s, 2H); ^{13}C NMR (75 MHz, DMSO-d6): δ = 166.99, 161.05, 150.73, 149.78, 141.59, 130.08, 129.64 (2C), 127.40 (2C), 68.90, 50.38; IR (ATR): ṽ = 3071, 2999, 2833, 2561, 1725, 1650, 1603, 1574, 1421, 1324, 1281, 1243, 1184, 889, 748, 619 cm^{-1}; HPLC: t_R 10.19 min; ESI-TOF-HRMS: m/z 372.9665 [M + H]$^+$, calculated for $C_{12}H_9IN_2O_4$ 371.9607, found: 371.9593.

Methyl 4-(3-cyanophenoxy)butanoate (6)

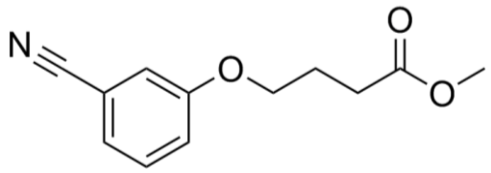

3-Cyanophenol (**10**) (1.00 g, 8.39 mmol, 1.0 eq.), methyl 4-bromobutyrate (1.67 g, 9.23 mmol, 1.1 eq.), anhydrous potassium carbonate (1.74 g, 12.59 mmol, 1.5 eq.) and anhydrous DMF (20 mL) were mixed in a microwave tube with a magnetic stir bar, sealed with a septum, and heated in a microwave at 100 °C for 2 h. After cooling down, the reaction mixture was diluted with ethyl acetate and washed with water and brine, dried over Na_2SO_4 and concentrated under reduced pressure. The crude product was purified by normal-phase flash chromatography.

Yield: 1.54 g, 7.01 mmol, 83 %, yellow oil; Purity: >99 %; ^{1}H NMR (300 MHz, DMSO-d6): δ = 7.50-7.45 (m, 1H), 7.40-7.36 (m, 2H), 7.27 (ddd, J = 8.3 Hz, J = 2.5 Hz, J = 1.3 Hz, 1H), 4.05 (t, J = 6.4 Hz, 2H), 3.60 (s, 3H), 2.47 (t, J = 7.4 Hz, 2H), 1.97 (ps. quin, J = 6.6 Hz, 2H); ^{13}C NMR (75 MHz, DMSO-d6): δ = 172.91, 158.55, 130.82, 124.47, 120.28, 118.62, 117.27, 112.22, 67.06, 51.33, 29.80, 23.96; IR (ATR): ṽ = 2953, 2359, 2230, 1736, 1598, 1579, 1436, 1263, 1048, 888, 792, 684, 631 cm^{-1}; HPLC: t_R 12.04 min.

Methyl 4-[3-(aminomethyl)phenoxy]butanoate (7f)

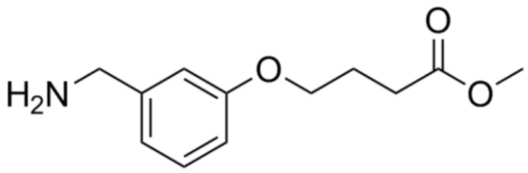

Methyl 4-(3-cyanophenoxy)butanoate (**6**) (1.00 g, 4.56 mmol, 1.0 eq.) was dissolved in MeOH (20 mL) under N_2 atmosphere. Palladium on active charcoal 10% (0.25 g, 0.23 mmol, 0.05 eq.) and 0.5 mL HCl 32 % were added to the stirred solution. The mixture was then stirred under H_2 atmosphere for 2 h at ambient temperature. The catalyst was filtered off and the solvent was removed from the filtrate under reduced pressure. The crude product was dissolved in water and the pH was adjusted to 8 with aqueous $NaHCO_3$ 10 %. The mixture was extracted with ethyl acetate. The combined organic phase was washed with water and brine, dried over Na_2SO_4 and concentrated under reduced pressure. The product was used for the next step without further purification.

Yield: 0.66 g, 2.96 mmol, 65 %, yellow oil; ^{1}H NMR (300 MHz, DMSO-d6): δ = 7.20 (t, J = 7.8 Hz, 1H), 6.97-6.83 (m, 2H), 6.75 (ddd, J = 8.2 Hz, J = 2.6 Hz, J = 1.0 Hz, 1H), 3.97 (t, J = 6.3 Hz, 2H), 3.70 (s, 2H), 3.61 (s, 3H), 2.47 (t, J = 7.3 Hz, 2H), 1.97 (ps. quin, J = 6.7 Hz, 2H); ^{13}C NMR (75 MHz, DMSO-d6): δ = 173.01, 158.46, 144.86, 129.09, 119.40, 113.21, 112.37, 66.27, 51.32, 45.21, 29.94, 24.26; HPLC: t_R 7.5 min; ESI-TOF-HRMS: *m/z* 224.1275 [M + H]$^+$, calculated for $C_{12}H_{17}NO_3$ 223.1208, found: 223.1203.

General procedure for amid coupling

Propiolic acid (1.5 eq.) was added dropwise to a stirred solution of EEDQ (1.5 eq.) and benzyl amine reactant (1.0 eq) in CH_2Cl_2 (15-20 mL/g benzyl amine reactant) at ambient temperature under N_2 atmosphere. The resulting mixture was stirred overnight at ambient temperature. Water (50 mL) was added and the solution was acidified with 2 M HCl. The mixture was extracted with CH_2Cl_2. The combined organic phase was washed with 1 M HCl, water and brine, dried over Na_2SO_4 and concentrated under reduced pressure. The crude product was purified by reversed-phase flash chromatography.

N-Benzylprop-2-ynamide[6] (8a)

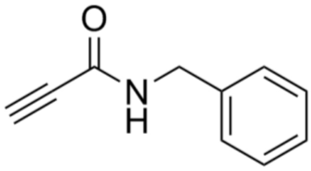

Yield: 2.27 g, 14.24 mmol, 78 %, colorless needles; Purity: >99 %; mp 92-94 °C; ^{1}H NMR (300 MHz, DMSO-d6): δ = 9.25 (t, J = 6.1 Hz, 1H), 7.36-7.23 (m, 5H), 4.29 (d, J = 6.1 Hz, 2H), 4.17 (s, 1H); ^{13}C NMR (75 MHz, DMSO-d6): δ = 151.65, 138.49, 128.34 (2C), 127.29 (2C), 126.98, 78.16, 76.01, 42.27; IR (ATR): ṽ = 3201, 3062, 2106, 1616, 1556, 1293, 1001, 748, 725, 696 cm^{-1}; HPLC: t_R 11.26 min; ESI-TOF-HRMS: *m/z* 160.0751 [M + H]$^+$, calculated for $C_{10}H_9NO$ 159.0684, found: 159.0678.

N-[(2-Fluorophenyl)methyl]prop-2-ynamide (8b)

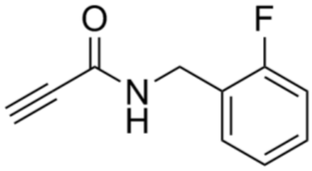

Yield: 0.33 g, 1.87 mmol, 43 %, colorless needles; Purity: >99 %; mp 90-92 °C; ^{1}H NMR (300 MHz, DMSO-d6): δ = 9.22 (t, J = 5.9 Hz, 1H), 7.37-7.29 (m, 2H), 7.20-7.14 (m, 2H), 4.34 (d, J = 5.9 Hz, 2H), 4.15 (s, 1H); ^{13}C NMR (75 MHz, DMSO-d6): δ = 159.96 (d, J = 244.9 Hz), 129.66 (d, J = 4.3 Hz), 129.14 (d, J = 8.1 Hz), 124.95 (d, J = 14.8 Hz), 124.30 (d, J = 3.5 Hz), 115.09 (d, J = 21.2 Hz), 77.96, 76.08, 36.14 (d, J = 4.5 Hz); ^{19}F NMR (282 MHz, DMSO-d6): δ = - 118.72-118.81 (m); IR (ATR): ṽ = 3229, 3069, 2108, 1653, 1617, 1588, 1551, 1488, 1456, 1436, 1274, 1229, 1181, 1108, 996, 843, 755, 697 cm^{-1}; HPLC: t_R 11.41 min; ESI-TOF-HRMS: *m/z* 178.0658 [M + H]$^+$, calculated for $C_{10}H_8FNO$: 177.0590, found: 177.0586.

N-[(3-Fluorophenyl)methyl]prop-2-ynamide (8c)

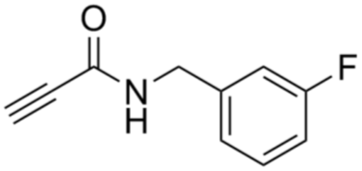

Yield: 0.25 g, 1.43 mmol, 33 %, colorless needles; Purity: >99 %; mp 75-77 °C; ^{1}H NMR (300 MHz, DMSO-d6): δ = 9.25 (t, J = 6.1 Hz, 1H), 7.41-7.34 (m, 1H), 7.11-7.05 (m, 3H), 4.31 (d, J = 6.1 Hz, 2H), 4.17 (s, 1H); ^{13}C NMR (75 MHz, DMSO-d6): δ = 162.12 (d, J = 243.5 Hz), 151.70, 141.40 (d, J = 7.4 Hz), 130.28 (d, J = 8.3 Hz), 123.21 (d, J = 2.7 Hz), 113.90 (d, J = 21.6 Hz), 113.71 (d, J = 20.9 Hz), 77.98, 76.15, 41.76 (d, J = 1.8 Hz); ^{19}F NMR (282 MHz, DMSO-d6): δ = - 113.44 (td, J = 9.7 Hz, J = 6.2 Hz); IR (ATR): ṽ = 3281, 3214, 3065, 2106, 1659, 1632, 1615, 1591, 1540, 1485, 1449, 1421, 1359, 1279, 1265, 1236, 1138, 1026, 945, 780, 758, 728, 680 cm^{-1}; HPLC: t_R 11.54 min; ESI-TOF-HRMS: m/z 178.0658 [M + H]$^{+}$, calculated for $C_{10}H_8FNO$: 177.0590, found: 177.0586.

_N_-[(4-Fluorophenyl)methyl]prop-2-ynamide (8d)

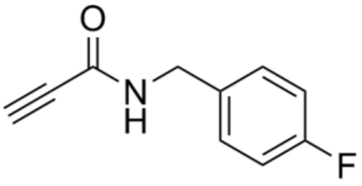

Yield: 0.21 g, 1.17 mmol, 27 %, colorless needles; Purity: >99 %; mp 77-79 °C; ^{1}H NMR (300 MHz, DMSO-d6): δ = 9.22 (t, J = 6.1 Hz, 1H), 7.32-7.25 (m, 2H), 7.19-7.11 (m, J = 9.0 Hz, 2H), 4.27 (d, J = 6.1 Hz, 2H), 4.15 (s, 1H); ^{13}C NMR (75 MHz, DMSO-d6): δ = 161.21 (d, J = 242.5 Hz), 151.60, 134.66 (d, J = 3.1 Hz), 129.30 (d, J = 8.2 Hz, 2C), 115.02 (d, J = 21.3 Hz, 2C), 78.06, 76.00, 41.55; ^{19}F NMR (282 MHz, DMSO-d6): δ = -115.85 (tt, J = 9.1 Hz, J = 5.6 Hz); IR (ATR): ṽ = 3288, 3040, 2360, 2325, 2114, 1622, 1527, 1509, 1259, 1219, 1159, 836, 682, 665 cm^{-1}; HPLC: t_R 11.57 min; ESI-TOF-HRMS: m/z 178.0655 [M + H]$^{+}$, calculated for $C_{10}H_8FNO$: 177.0590, found: 177.0583.

_N_-[(3-Methoxyphenyl)methyl]prop-2-ynamide (8e)

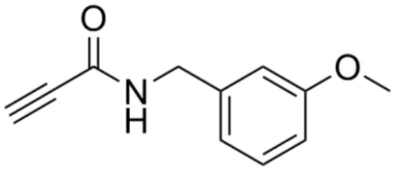

Yield: 0.99 g, 5.25 mmol, 68 %, colorless crystals: Purity: >99 %; mp 63-65 °C; ^{1}H NMR (300 MHz, DMSO-d6): δ = 9.23 (t, J = 5.7 Hz, 1H), 7.28-7.20 (m, 1H), 6.85-6.79 (m, 3H), 4.26 (d, J

= 6.1 Hz, 2H), 4.16 (s, 1H), 3.74 (s, 3H); ^{13}C NMR (75 MHz, DMSO-d6): δ = 159.30, 151.66, 140.06, 129.44, 119.42, 112.99, 112.36, 78.15, 76.04, 54.98, 42.21; IR (ATR): ṽ = 3265, 3053, 2110, 1641, 1603, 1543, 1282, 1261, 1164, 1032, 789, 689 cm^{-1}; HPLC: t_R 10.97 min; ESI-TOF-HRMS: *m/z* 190.0853 [M + H]$^+$, calculated for $C_{11}H_{11}NO_2$ 189.0790, found: 189.0780.

Methyl 4-[3-(prop-2-ynamidomethyl)phenoxy]butanoate (8f)

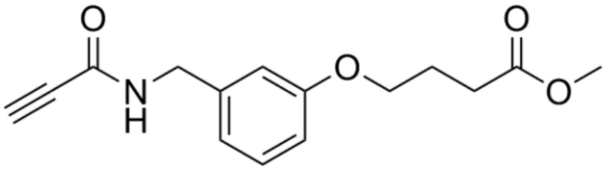

Yield: 0.52 g, 1.90 mmol, 85 %, yellow oil; Purity: >95 %; ^{1}H NMR (300 MHz, DMSO-d6): δ = 9.22 (t, *J* = 6.0 Hz, 1H), 7.27-7.16 (m, 1H), 6.84-6.75 (m, 3H), 4.25 (d, *J* = 6.1 Hz, 2H), 4.17 (s, 1H), 3.96 (t, *J* = 6.3 Hz, 2H), 3.61 (s, 3H), 2.47 (t, *J* = 7.3 Hz, 2H), 1.97 (m, 2H); ^{13}C NMR (75 MHz, DMSO-d6): δ = 173.00, 158.48, 151.64, 140.07, 129.43, 119.48, 113.51, 112.81, 78.14, 76.05, 66.36, 51.32, 42.18, 29.94, 24.22; IR (ATR): ṽ = 3280, 2918, 2360, 2109, 1734, 1652, 1541, 1456, 1266, 1172, 1053, 892, 631 cm^{-1}; HPLC: t_R 11.83 min; ESI-TOF-HRMS: *m/z* 276.1225 [M + H]$^+$, calculated for $C_{15}H_{17}NO_4$: 275.1158, found: 275.1153.

4-[3-(Prop-2-ynamidomethyl)phenoxy]butanoic acid (8g)

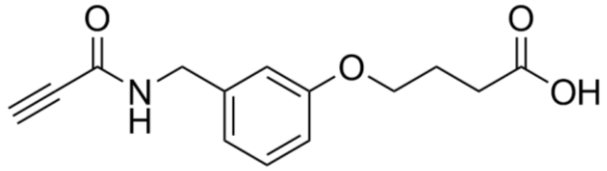

Lithium hydroxide monohydrate (0.62 g, 14.75 mmol, 2 eq.) was added to a stirred solution of **8f** (2.03 g, 7.37 mmol, 1 eq.) in THF (60 mL) and water (15 mL) at 0 °C. The reaction mixture was allowed to warm ambient temperature and stirred for 3 h. The reaction mixture was concentrated under reduced pressure, neutralized with 1 M HCl and extracted with ethyl acetate. The combined organic phase was washed with water and brine, dried over Na_2SO_4 and concentrated under reduced pressure. The crude product was purified by normal-phase flash chromatography.

Yield: 0.90 g, 3.46 mmol, 47 %, white solid; Purity: >99 %; mp 103-105 °C; ^{1}H NMR (300 MHz, DMSO-d6): δ = 12.15 (s, 1H), 9.23 (t, J = 6.1 Hz, 1H), 7.29-7.16 (m, 1H), 6.87-6.75 (m, 3H), 4.25 (d, J = 6.1 Hz, 2H), 4.18 (s, 1H), 3.95 (t, J = 6.4 Hz, 2H), 2.38 (t, J = 7.3 Hz, 2H), 1.93 (ps. quin, J = 6.9 Hz, 2H); ^{13}C NMR (75 MHz, DMSO-d6): δ = 174.07, 158.55, 151.64, 140.05, 129.44, 113.52, 112.85, 78.15, 76.08, 66.46, 42.19, 30.11, 24.25; IR (ATR): ṽ = 3272, 2940, 2361, 2112, 1688, 1628, 1614, 1586, 1539, 1454, 1269, 1161, 1072, 1062, 1008, 924, 773, 711, 688, 670 cm^{-1}; HPLC: t_R 10.25 min; ESI-TOF-HRMS: *m/z* 262.1070 $[M + H]^+$, calculated for $C_{14}H_{15}NO_4$: 261.1001, found: 261.0998.

General procedure for Sonogashira cross-coupling

The iodouracil intermediate (100 mg, 1.0 eq.) and the alkyne intermediate (1.5 eq.) were dissolved in anhydrous DMF (2 mL). Stirred, at ambient temperature and under N_2 atmosphere, $Pd(PPh_3)_4$ (0.1 eq.), CuI (0.2 eq.) and triethylamine (0.5 mL) were added in succession. The resulting mixture was continued to stir for 2 h at ambient temperature. The reaction was quenched by adding water. The aqueous phase was acidified with 2 M HCl and extracted with DCM. The combined organic phase was washed with water and brine, dried over Na_2SO_4 and concentrated under reduced pressure. The crude product was purified by reverse-phase flash chromatography and recrystallization from MeOH.

***N*-Benzyl-3-(1-benzyl-2,4-dioxo-1,2,3,4-tetrahydropyrimidin-5-yl)prop-2-ynamide (2)**

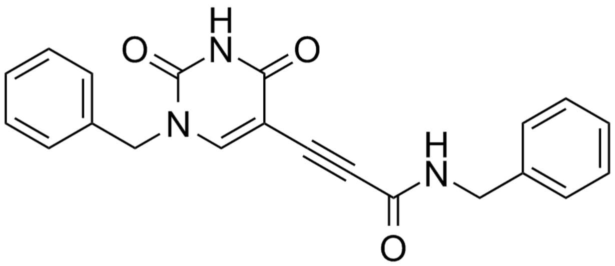

The iodouracil intermediate **5a** (300 mg, 0.91 mmol, 1.0 eq.) and the alkyne intermediate **8a** (218 mg, 1.37 mmol, 1.5 eq.) were dissolved in anhydrous DMF (4 mL) under N_2 atmosphere. $Pd(PPh_3)_4$ (106 mg, 0.091 mmol, 0.1 eq.), CuI (35 mg, 0.183 mmol, 0.2 eq.) and triethylamine (1 mL) were added at 20 °C. The resulting mixture was continued to stir for 2 h at 20 °C. The reaction mixture was quenched by adding water. The aqueous phase was acidified with 2 M HCl and extracted with CH_2Cl_2. The organic phase was washed with water

and brine, dried over Na_2SO_4 and concentrated under reduced pressure. The crude product was purified by reversed-phase flash chromatography and recrystallization from MeOH.

Yield: 121 mg, 0.34 mmol, 37 %, colorless crystals; Purity: >99 %; dp 195 °C; ^{1}H NMR (800 MHz, DMSO-d6): δ = 11.82 (s, 1H), 9.19 (t, J = 6.2 Hz, 1H), 8.43 (s, 1H), 7.38-7.35 (m, 2H), 7.34-7.30 (m, 5H), 7.27-7.23 (m, 3H), 4.92 (s, 2H), 4.31 (d, J = 6.2 Hz, 2H); ^{13}C NMR (201 MHz, DMSO-d6): δ = 161.57, 152.23, 151.55, 149.90, 138.74, 136.21, 128.70 (2C), 128.33 (2C), 127.89, 127.66 (2C), 127.33 (2C), 126.95, 95.47, 86.91, 77.50, 51.01, 42.36; IR (ATR): ṽ = 3334, 3170, 3035, 2846, 2227, 1721, 1672, 1639, 1543, 1429, 1282, 1250, 1026, 969, 728, 696 cm^{-1}; HPLC: t_R 12.74 min; ESI-TOF-HRMS: *m/z* 382.1152 [M + Na]$^+$, calculated for $C_{21}H_{17}N_3O_3$: 359.1270, found: 359.1259; Anal. calculated for $C_{21}H_{17}N_3O_3$ [%]: C 70.18, H 4.77, N 11.69, found [%]: C 70.22, H 4.69, N 11.66.

4-{[5-(2-{[(3-methoxyphenyl)methyl]carbamoyl}eth-1-yn-1-yl)-2,4-dioxo-1,2,3,4-tetrahydropyrimidin-1-yl]methyl}benzoic acid (3)

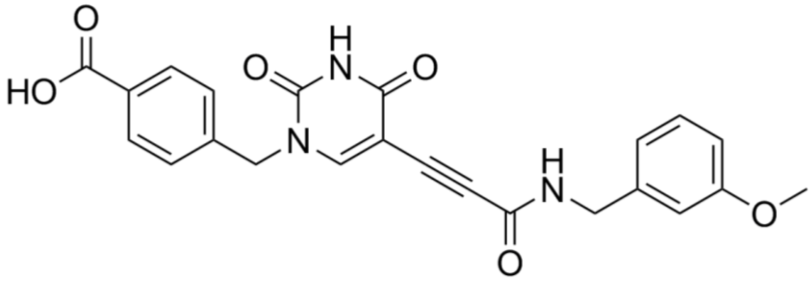

The iodouracil intermediate **5b** (250 mg, 0.67 mmol, 1.0 eq.) and the alkyne intermediate **8e** (191 mg, 1.01 mmol, 1.5 eq.) were dissolved in anhydrous DMF (4 mL) under N_2 atmosphere. $Pd(PPh_3)_4$ (78 mg, 0.067 mmol, 0.1 eq.), CuI (26 mg, 0.134 mmol, 0.2 eq.) and triethylamine (1 mL) were added at 20 °C. The resulting mixture was continued to stir for 2 h at 20 °C. The reaction mixture was quenched by adding water. The resulting suspension was acidified with 2 M HCl. The precipitate was filtered off, washed with water, suspended in MeOH and cooled to -20 °C. Again the precipitate was filtered off and washed with cold MeOH. The crude product was purified by reversed-phase flash chromatography and recrystallization from MeOH.

Yield: 125 mg, 0.29 mmol, 43 %, white solid; Purity: >99 %; dp 207 °C; ^{1}H NMR (800 MHz, DMSO-d6): δ = 13.05 (s, 1H), 11.84 (s, 1H), 9.17 (t, J = 6.2 Hz, 1H), 8.46 (s, 1H), 7.93 (d, J = 8.3 Hz, 2H), 7.43 (d, J = 8.3 Hz, 2H), 7.25-7.23 (m, 1H), 6.83-6.81 (m, 3H), 4.99 (s, 2H), 4.28 (d, J = 6.2 Hz, 2H), 3.73 (s, 3H); ^{13}C NMR (201 MHz, DMSO-d6): δ = 167.11, 161.61, 159.30, 152.23, 151.66, 149.91, 140.99, 140.31, 130.51, 129.65 (2C), 129.43, 127.51 (2C), 119.46,

113.04, 112.31, 95.64, 86.94, 77.52, 55.00, 50.92, 42.32; IR (ATR): ṽ = 3457, 3355, 3036, 2846, 2220, 1675, 1636, 1286, 1148, 1048, 779, 691 cm^{-1}; HPLC: t_R 11.79 min; ESI-TOF-HRMS: *m/z* 456.1157 $[M + Na]^+$, calculated for $C_{23}H_{19}N_3O_6$: 433.1274, found: 433.1264; Anal. calculated for $C_{23}H_{19}N_3O_6$ [%]: C 63.74, H 4.42, N 9.70, found [%]: C 63.73, H 4.37, N 9.81.

4-({5-[2-({[3-(3-Carboxypropoxy)phenyl]methyl}carbamoyl)eth-1-yn-1-yl]-2,4-dioxo-1,2,3,4-tetrahydropyrimidin-1-yl}methyl)benzoic acid (4)

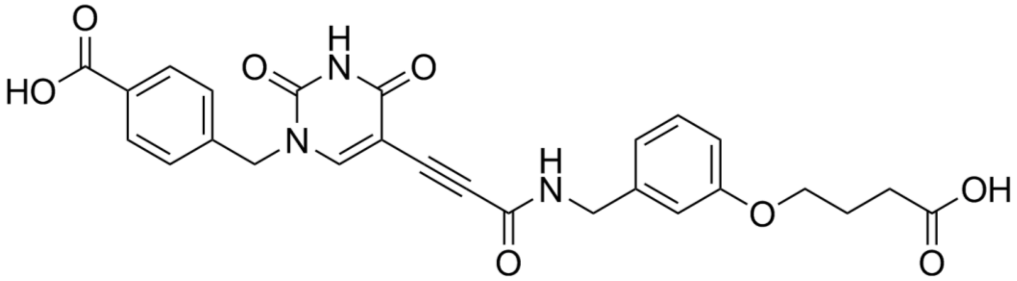

The iodouracil intermediate **5b** (550 mg, 1.48 mmol, 1.0 eq.) and the alkyne intermediate **8g** (405 mg, 1.55 mmol, 1.05 eq.) were dissolved in anhydrous DMF (8 mL) under N_2 atmosphere. $Pd(PPh_3)_4$ (171 mg, 0.15 mmol, 0.1 eq.), CuI (56 mg, 0.30 mmol, 0.2 eq.) and triethylamine (2 mL) were added at 20 °C. The resulting mixture was continued to stir for 4 h at 20 °C. The reaction mixture was quenched by adding water. The resulting suspension was acidified with 2 M HCl. The precipitate was filtered off, washed with water, suspended in MeOH and cooled to -20 °C. Again the precipitate was filtered off and washed with cold MeOH. The crude product was purified by reversed-phase flash chromatography and recrystallization from MeOH/water (10:1).

Yield: 295 mg, 0.58 mmol, 39 %, white solid; Purity: >99 %; dp 224 °C; ^1H NMR (700 MHz, DMSO-d6): δ = 12.55 (s, 2H), 11.84 (s, 1H), 9.16 (t, J = 6.2 Hz, 1H), 8.46 (s, 1H), 7.93 (d, J = 7.9 Hz, 2H), 7.44 (d, J = 7.6 Hz, 2H), 7.25-7.20 (m, 1H), 6.83-6.79 (m, 3H), 4.99 (s, 2H), 4.27 (d, J = 6.2 Hz, 2H), 3.95 (t, J = 6.2 Hz, 2H), 2.39 (t, J = 7.8 Hz, 2H), 1.93 (ps. quin, J = 6.8 Hz, 2H); ^{13}C NMR (176 MHz, DMSO-d6): δ = 174.06, 166.99, 161.60, 158.56, 152.21, 151.65, 149.90, 141.10, 140.30, 130.22, 129.70 (2C), 129.43, 127.59 (2C), 119.48, 113.59, 112.77, 95.64, 86.94, 77.52, 66.48, 50.92, 42.30, 30.16, 24.32; IR (ATR): ṽ = 3334, 3021, 2900, 2837, 2227, 1741, 1706, 1673, 1644, 1461, 1291, 1272, 1253, 1152, 1042, 868, 747 cm^{-1}; HPLC: t_R 10.98 min; ESI-TOF-HRMS: *m/z* 506.1549 $[M + H]^+$, calculated for $C_{26}H_{23}N_3O_8$: 505.1485, found: 505.1475; Anal. calculated for $C_{26}H_{23}N_3O_8$ [%]: C 61.78, H 4.59, N 8.31, found [%]: C 61.55, H 4.54, N 8.24.

3-(1-Benzyl-2,4-dioxo-1,2,3,4-tetrahydropyrimidin-5-yl)-N-[(2-fluorophenyl)methyl]prop-2-ynamide (9a)

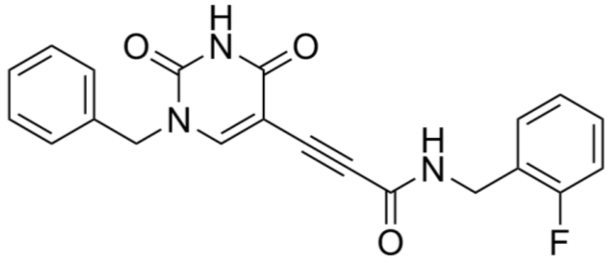

This compound was synthesized out of **5a** and **8b** following the general procedure for Sonogarshira cross-coupling.

Yield: 70.0 mg, 0.19 mmol, 61 %, colorless needles; Purity: >99 %; dp 144 °C; ^{1}H NMR (300 MHz, DMSO-d6): δ = 11.81 (s, 1H), 9.17 (t, J = 5.9 Hz, 1H), 8.42 (s, 1H), 7.43-7.27 (m, 7H), 7.21-7.12 (m, 2H), 4.92 (s, 2H), 4.35 (d, J = 5.9 Hz, 2H); ^{13}C NMR (75 MHz, DMSO-d6): δ = 161.51, 159.99 (d, J = 245.1 Hz), 152.26, 151.57, 149.87, 136.17, 129.69 (d, J = 4.2 Hz), 129.13 (d, J = 8.1 Hz), 128.67 (2C), 127.86, 127.63 (2C), 125.19 (d, J = 14.8 Hz), 124.33 (d, J = 3.5 Hz), 115.12 (d, J = 21.1 Hz), 95.40, 86.74, 77.63, 51.00, 36.24 (d, J = 4.7 Hz); ^{19}F NMR (282 MHz, DMSO-d6): δ = -118.72-118.80 (m); IR (ATR): ṽ = 3613, 3419, 3041, 2853, 2360, 2216, 1707, 1674, 1634, 1293, 870, 752, 697 cm^{-1}; HPLC: t_R 12.81 min; ESI-TOF-HRMS: m/z 378.1242 [M + H]$^{+}$, calculated for $C_{21}H_{16}FN_3O_3$: 377.1176, found: 377.1168; Anal. calculated for $C_{21}H_{16}FN_3O_3$ [%]: C 66.84, H 4.27, N 11.13, found [%]: C 66.67, H 4.05, N 11.02.

3-(1-Benzyl-2,4-dioxo-1,2,3,4-tetrahydropyrimidin-5-yl)-N-[(3-fluorophenyl)methyl]prop-2-ynamide (9b)

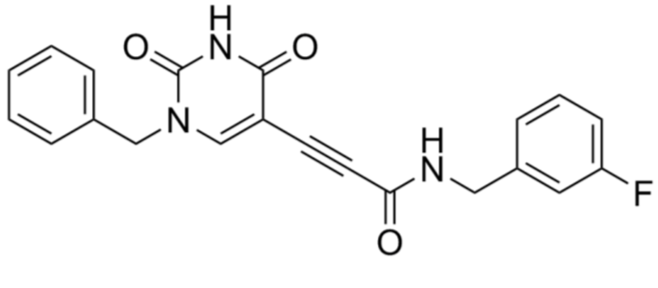

This compound was synthesized out of **5a** and **8c** following the general procedure for Sonogarshira cross-coupling.

Yield: 79.3 mg, 0.21 mmol, 69 %, colorless needles; Purity: >99 %; dp 185 °C; ^{1}H NMR (300 MHz, DMSO-d6): δ = 11.82 (s, 1H), 9.22 (t, J = 6.1 Hz, 1H), 8.44 (s, 1H), 7.41-7.31 (m, 6H),

7.11-7.05 (m, 3H), 4.92 (s, 2H), 4.32 (d, *J* = 6.1 Hz, 2H); ^{13}C NMR (75 MHz, DMSO-d6): *δ* = 161.96 (d, *J* = 243.4 Hz), 161.55, 152.32, 151.63, 149.89, 141.70 (d, *J* = 7.2 Hz), 136.18, 130.32 (d, *J* = 8.4 Hz), 128.69 (2C), 127.88, 127.65 (2C), 123.29 (d, *J* = 2.7 Hz), 113.96 (d, *J* = 21.7 Hz), 113.72 (d, *J* = 21.0 Hz), 95.38, 86.77, 77.73, 51.03, 41.88; ^{19}F NMR (282 MHz, DMSO-d6): *δ* = -113.42-113.51 (m); IR (ATR): ṽ = 3338, 3033, 2924, 2850, 2360, 2226, 1722, 1669, 1632, 1541, 1284, 867, 698 cm^{-1}; HPLC: t_R 12.87 min; ESI-TOF-HRMS: *m/z* 378.1242 [M + H]$^+$, calculated for $C_{21}H_{16}FN_3O_3$: 377.1176, found: 377.1168.

3-(1-Benzyl-2,4-dioxo-1,2,3,4-tetrahydropyrimidin-5-yl)-N-[(4-fluorophenyl)methyl]prop-2-ynamide (9c)

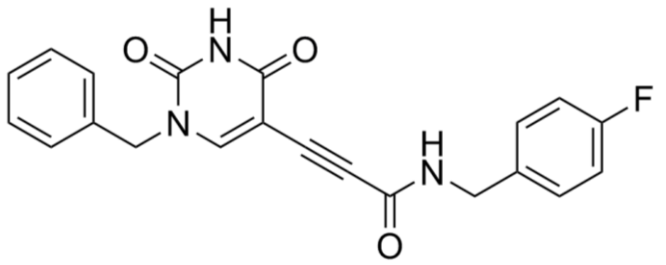

This compound was synthesized out of **5a** and **8d** following the general procedure for Sonogarshira cross-coupling.

Yield: 79.6 mg, 0.21 mmol, 69 %, colorless needles; Purity: >99 %; dp 195 °C; ^{1}H NMR (300 MHz, DMSO-d6): *δ* = 11.79 (s, 1H), 9.16 (t, *J* = 6.1 Hz, 1H), 8.41 (s, 1H), 7.41-7.26 (m, 7H), 7.19-7.10 (m, *J* = 9.0 Hz, 2H), 4.92 (s, 2H), 4.28 (d, *J* = 6.1 Hz, 2H); ^{13}C NMR (75 MHz, DMSO-d6): *δ* = 161.58, 161.25 (d, *J* = 242.4 Hz), 152.24, 151.59, 149.91, 136.20, 134.95 (d, *J* = 3.0 Hz), 129.39 (d, *J* = 8.2 Hz), 128.72 (2C), 127.91, 127.66 (2C), 115.08 (d, *J* = 21.3 Hz), 95.43, 86.86, 77.59, 51.04, 41.69; ^{19}F NMR (282 MHz, DMSO-d6): *δ* = -115.91 (tt, *J* = 9.1 Hz, *J* = 5.5 Hz); IR (ATR): ṽ = 3319, 2989, 2849, 2361, 2326, 2228, 1719, 1670, 1630, 1542, 1278, 1227, 817, 695 cm^{-1}; HPLC: t_R 12.87 min; ESI-TOF-HRMS: *m/z* 378.1242 [M + H]$^+$, calculated for $C_{21}H_{16}FN_3O_3$: 377.1176, found: 377.1168; Anal. calculated for $C_{21}H_{16}FN_3O_3$ [%]: C 66.84, H 4.27, N 11.13, found [%]: C 66.59, H 4.05, N 11.17.

Biological Assays

Enzo Life Sciences drug discovery kits

The following assay kits were purchased from Enzo Life Sciences (http://www.enzolifesciences.com) and used to test the synthesized inhibitors for their inhibitory activity against the catalytic domains of MMP-1, MMP-2, MMP-3, MMP-7, MMP-8, MMP-9, MMP-12, MMP-13 and MMP-14: BML-AK404-0001, MMP-1 colorimetric drug discovery kit, enzyme concentration of 15.3 U per well; BML-AK408-0001, MMP-2 colorimetric drug discovery kit, enzyme concentration of 1.16 U per well; BML-AK400-0001, MMP-3 colorimetric drug discovery kit, enzyme concentration of 2 U per well; BML-AK406-0001, MMP-7 colorimetric drug discovery kit, enzyme concentration of 1.28 U per well; BML-AK414-0001, MMP-8 colorimetric drug discovery kit, enzyme concentration of 1.84 U per well; BML-AK410-0001, MMP-9 colorimetric drug discovery kit, enzyme concentration of 0.9 U per well; BML-AK402-0001, MMP-12 colorimetric drug discovery kit, enzyme concentration of 0.7 U per well; BML-AK412-0001, MMP-13 colorimetric drug discovery kit, enzyme concentration of 1.38 U per well; BML-AK416-0001, MMP-14 colorimetric drug discovery kit, enzyme concentration of 2.4 U per well.

All test compounds were dissolved in DMSO. The final amount of DMSO in the reaction was 5 % (v/v). Single dose measurements were performed at a compound concentration of 6.5 µM. For IC_{50} determinations a serial dilution consisting of seven concentrations was measured. To get 100 % activity of the enzyme, control measurements containing buffer, enzyme, substrate and 5 % DMSO were measured.

Reaction Biology Corp. – Protease Assays

The fluorometric assays were carried out by Reaction Biology Corp. (http://www.reactionbiology.com) using the catalytic domain of the proteases and the FRET peptide 5-FAM/QXLTM as substrate. Enzyme concentration in the assays were as follows: MMP-1, 1.48 nM; MMP-2, 3.78 nM; MMP-3, 7.01 nM; MMP-7, 1.44 nM; MMP-8, 1.73 nM; MMP-9, 1.60 nM; MMP-12, 0.37 nM; MMP-13, 0.66 nM; MMP-14, 4.39 nM. Buffer: 50 mM HEPES (pH 7.5), 10 mM $CaCl_2$, 0.01 % Brij-35, add 0.1 mg/mL BSA and 1 % DMSO before use.

Compounds tested by Reaction Biology Corp. were dissolved in DMSO. The final amount of DMSO in the reaction was 1.1 % (v/v). Single dose measurements were performed at a compound concentration of 10 µM. For IC_{50} determinations a serial dilution consisting of ten concentrations was measured.

IC_{50} measurements:

***N*-Benzyl-3-(1-benzyl-2,4-dioxo-1,2,3,4-tetrahydropyrimidin-5-yl)prop-2-ynamide (2)**

The scaffold **2** was measured in-house using the colorimetric drug discovery kit from Enzo Life Sciences due to its bad solubility: No reliable measurements for *c*(**2**) > 20 µM.

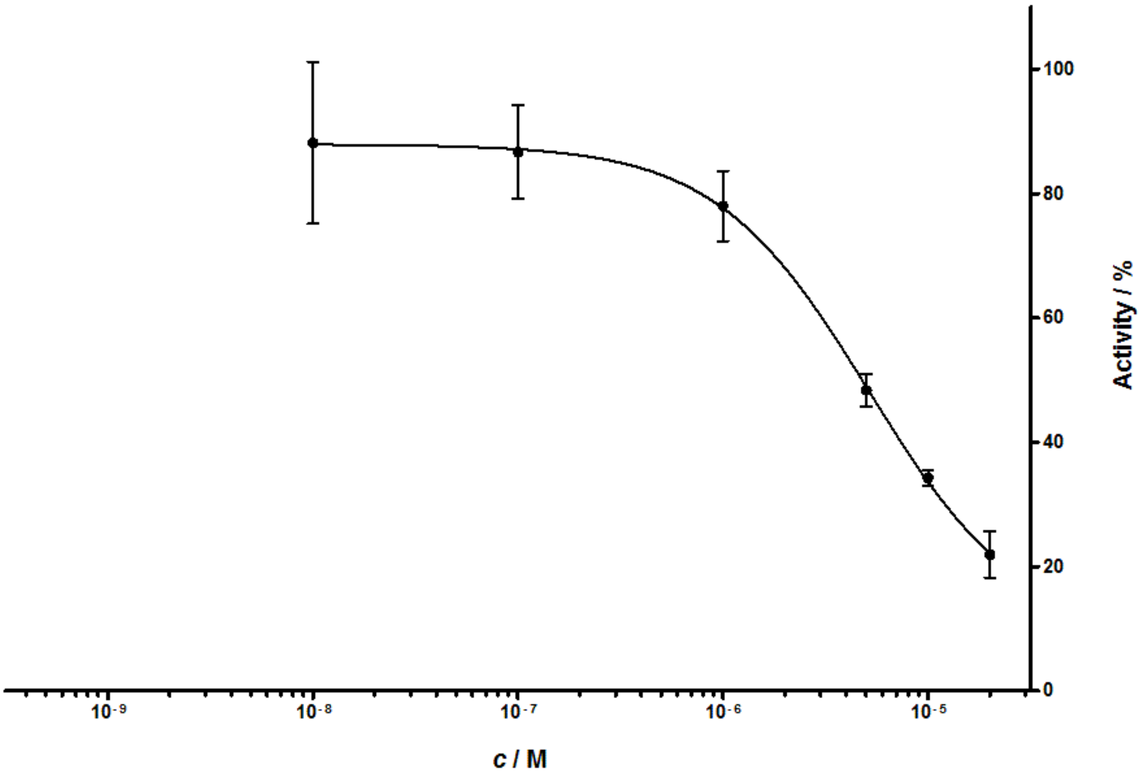

Best-fit values (Sigmoidal dose-response):

Bottom	8.724
Top	87.94
LogIC50	-5.290 ± 0.1652
Hill Slope	-1.165
**IC50**	**5.126E-06 M**<br>**2.266E-06 – 1.159E-05 M (95% CI)**
R square	0.9558

4-{[5-(2-{[(3-methoxyphenyl)methyl]carbamoyl}eth-1-yn-1-yl)-2,4-dioxo-1,2,3,4-tetrahydropyrimidin-1-yl]methyl}benzoic acid (3)

Measured by Reaction Biology Corp.

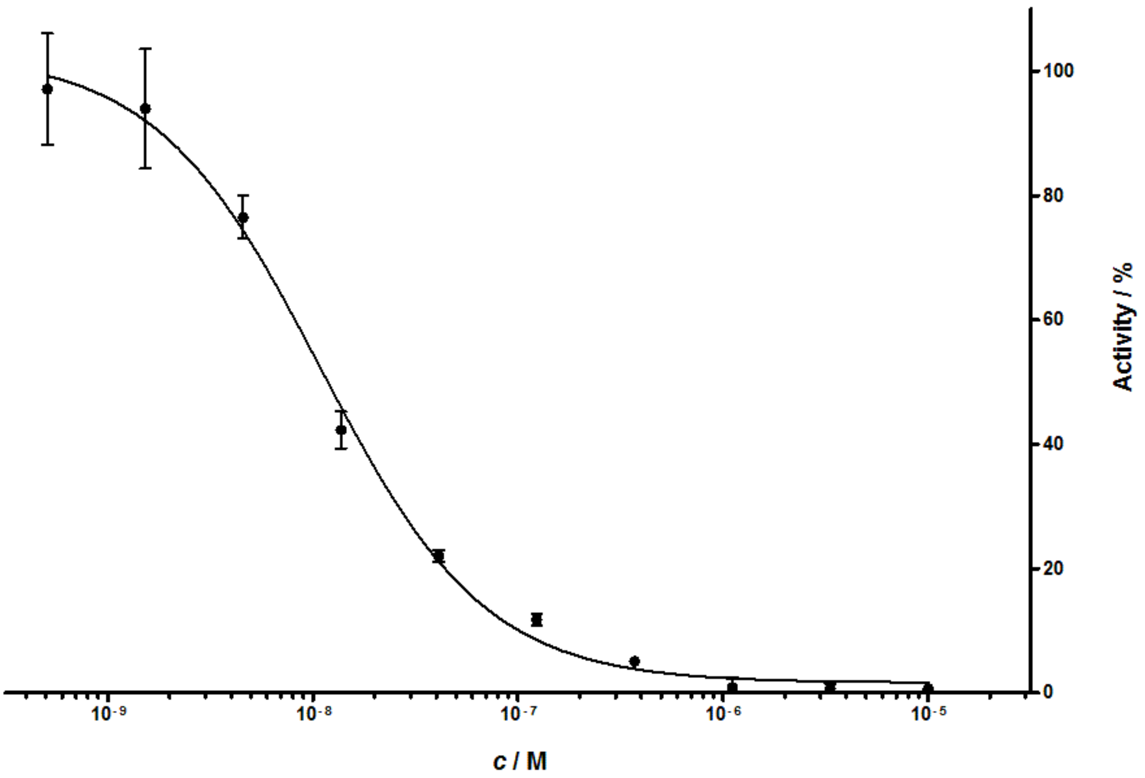

Best-fit values (Sigmoidal dose-response):

Bottom	1.677
Top	102.8
LogIC50	-7.963 ± 0.04705
Hill Slope	-1.080
**IC50**	**1.090E-08 M**<br>**8.723E-09 – 1.362E-08 M (95% CI)**
R square	0.9877

4-({5-[2-({[3-(3-Carboxypropoxy)phenyl]methyl}carbamoyl)eth-1-yn-1-yl]-2,4-dioxo-1,2,3,4-tetrahydropyrimidin-1-yl}methyl)benzoic acid (4)

Measured by Reaction Biology Corp.

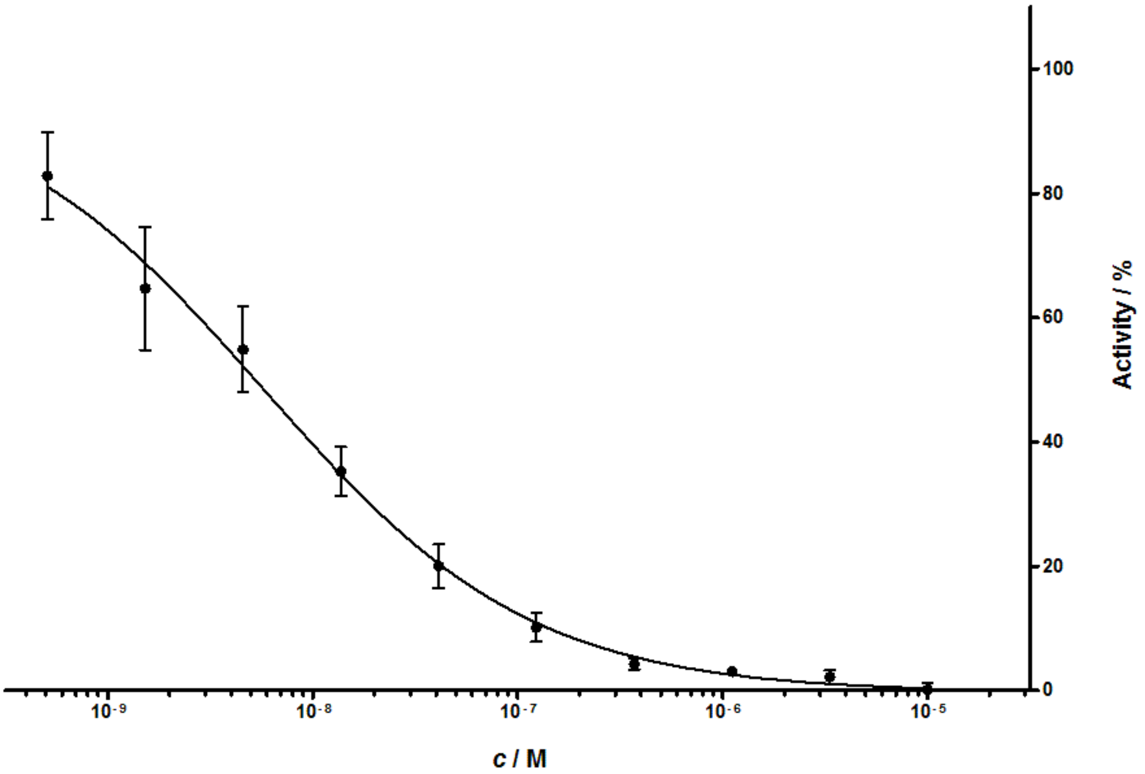

Best-fit values (Sigmoidal dose-response):

Bottom	-0.2977
Top	97.20
LogIC50	-8.238 ± 0.1530
Hill Slope	-0.6662
**IC50**	**5.775E-09 M**<br>**2.799E-09 – 1.191E-08 M (95% CI)**
R square	0.9778

Profiling:

Anti-Target	Activity		
	**2**[a]	**3**[a]	**4**[b]
MMP 1	93 ± 5	116 ± 29	103 ± 1
MMP 2	101 ± 4	76 ± 21	96 ± 2
MMP 3	96 ± 4	85 ± 18	102 ± 1
MMP 7	102 ± 3	107 ± 23	104 ± 2
MMP 8	96 ± 7	108 ± 22	113 ± 2
MMP 9	104 ± 5	96 ± 11	107 ± 3
MMP 12	100 ± 5	106 ± 29	113 ± 4
MMP 14	95 ± 3	109 ± 28	101 ± 1

Remaining activity of anti-targets in % ± SD; [a] Triplicates measured in-house at *c*(inhibitor) = 20 µM; [b] Triplicates measured by Reaction Biology Corp. at *c*(inhibitor) = 10 µM.

Analytical Data

NMR spectra

1-Benzyl-5-iodo-1,2,3,4-tetrahydropyrimidine-2,4-dione (5a)

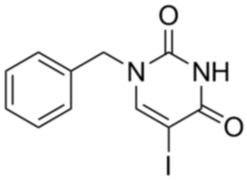

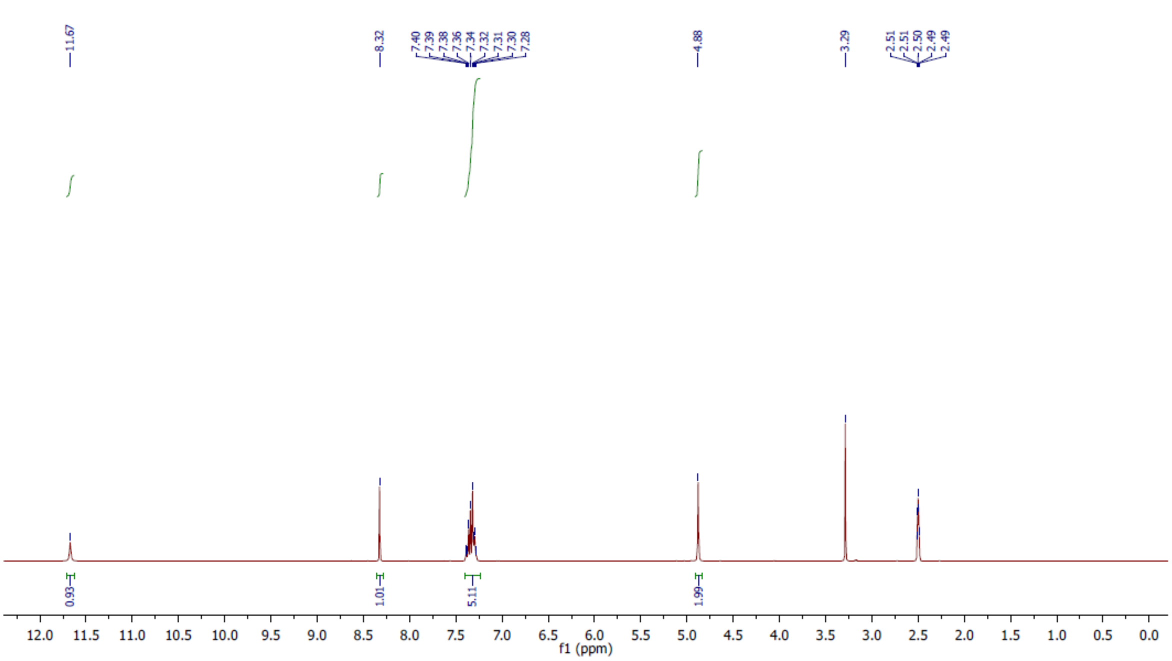

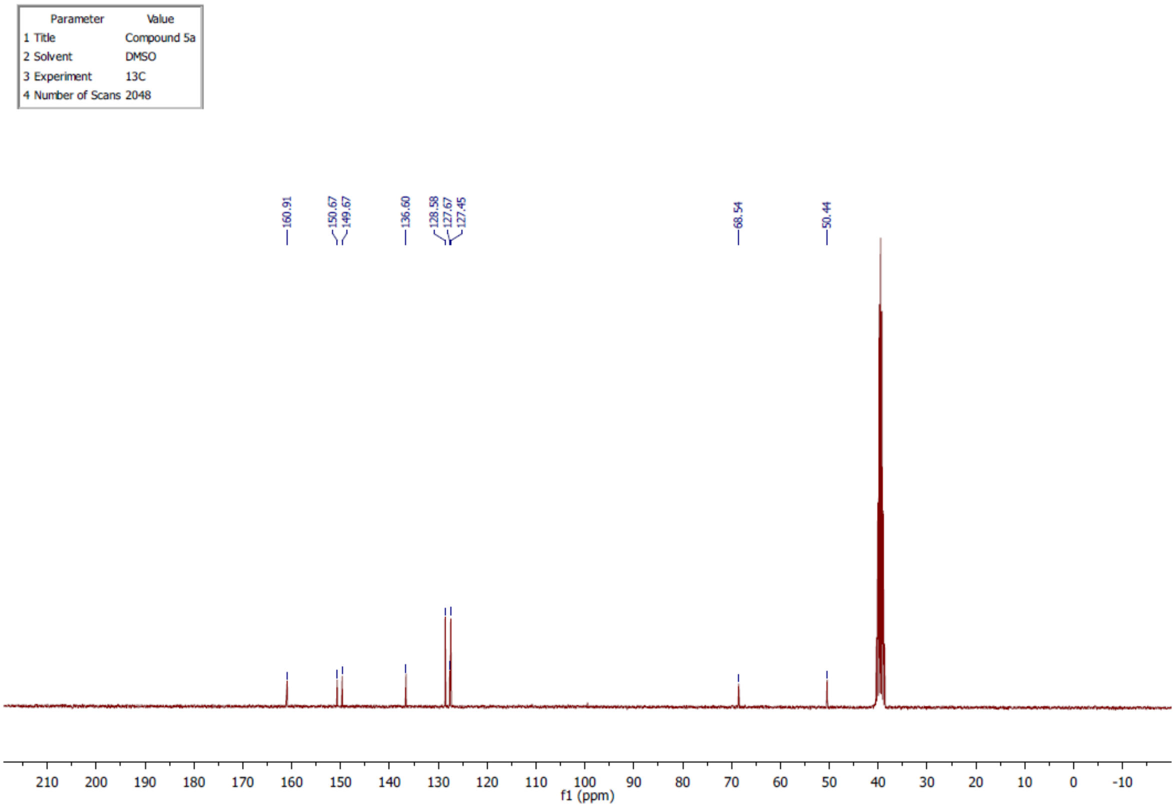

Parameter	Value
1 Title	Compound 5a
2 Solvent	DMSO
3 Experiment	DEPT-135
4 Number of Scans	512

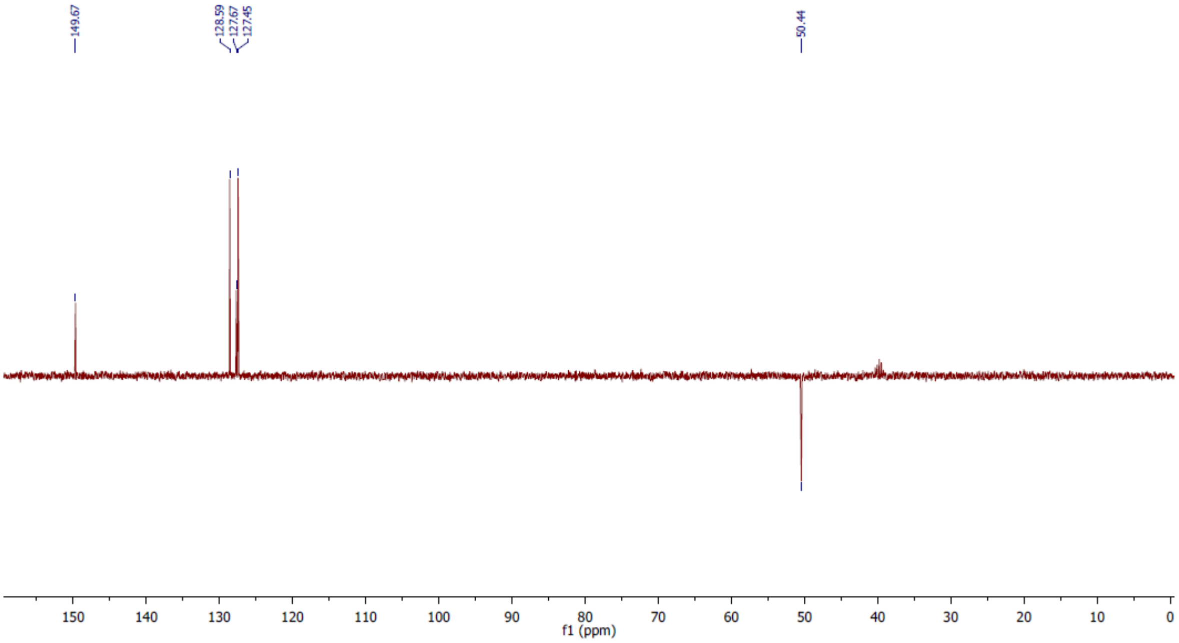

Parameter	Value
1 Title	Compound 5a
2 Solvent	DMSO
3 Experiment	DEPT-90
4 Number of Scans	512

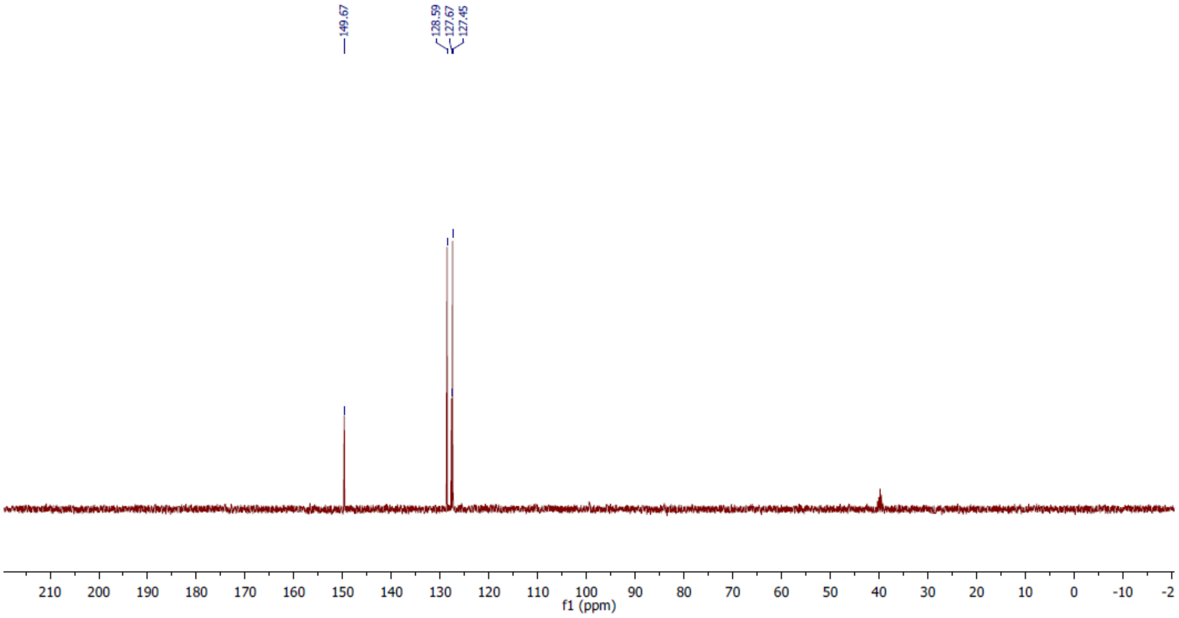

4-[(5-Iodo-2,4-dioxo-1,2,3,4-tetrahydropyrimidin-1-yl)methyl]benzoic acid (5b)

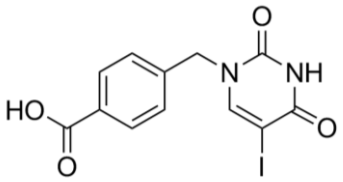

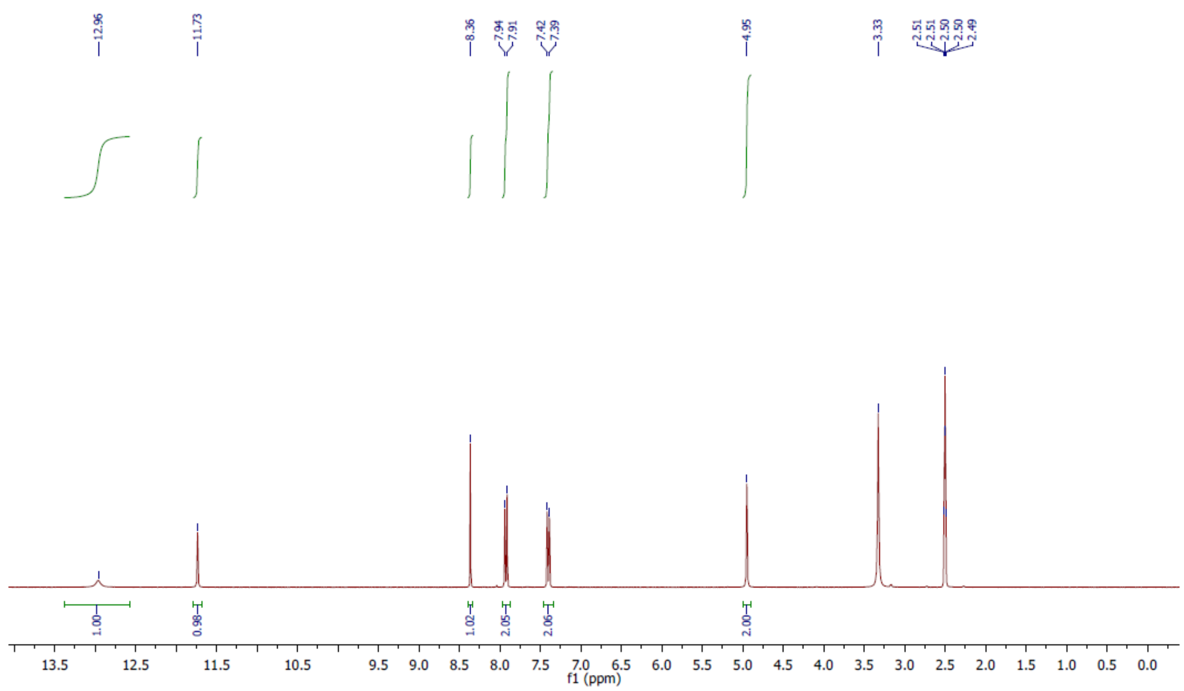

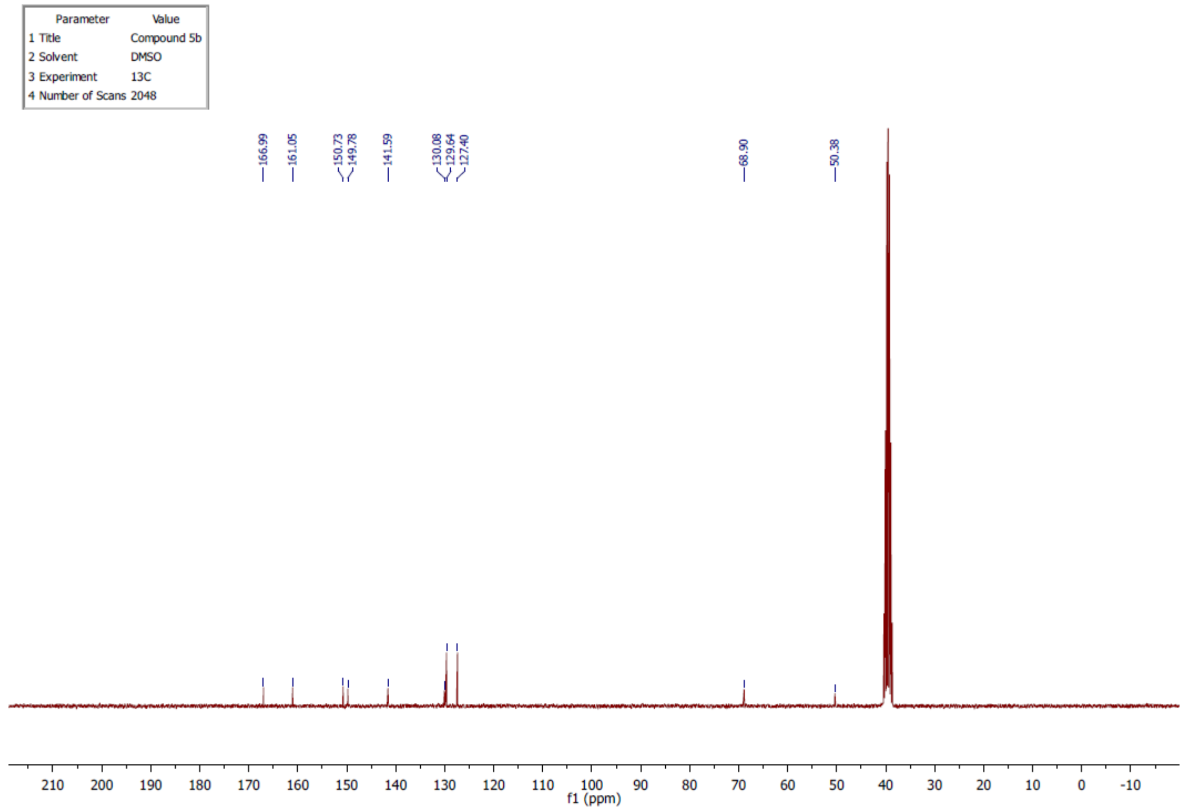

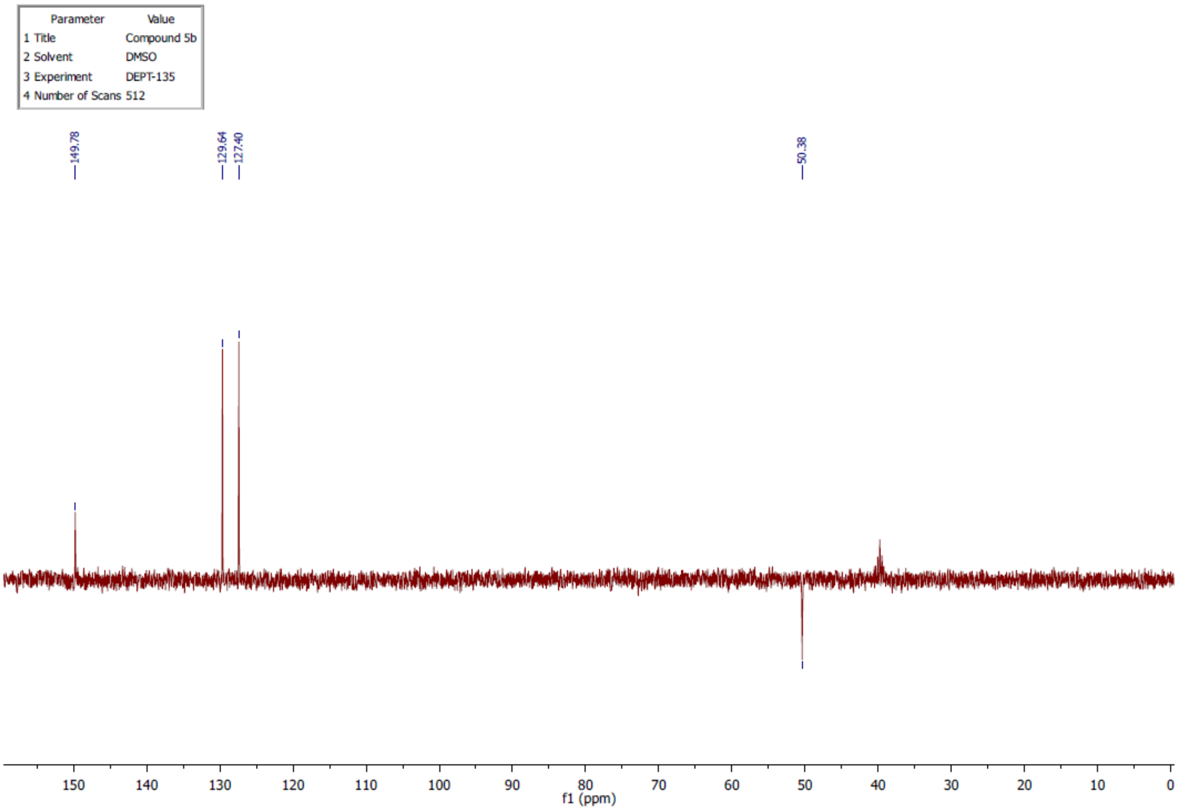
Parameter Value
1 Title Compound 5b
2 Solvent DMSO
3 Experiment DEPT-135
4 Number of Scans 512
149.78
129.64
127.40
50.38
150 140 130 120 110 100 90 80 70 60 50 40 30 20 10 0
f1 (ppm)

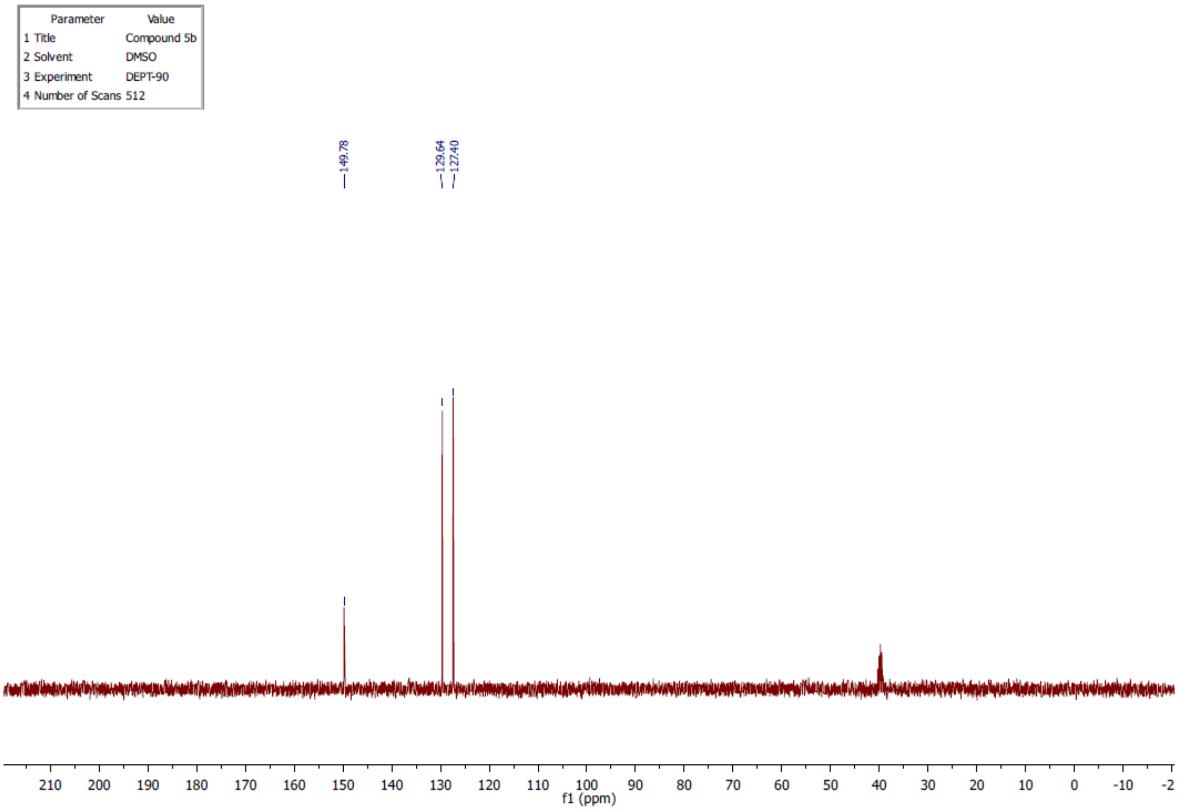
Parameter Value
1 Title Compound 5b
2 Solvent DMSO
3 Experiment DEPT-90
4 Number of Scans 512
149.78
129.64
127.40
210 200 190 180 170 160 150 140 130 120 110 100 90 80 70 60 50 40 30 20 10 0 -10 -2
f1 (ppm)

Methyl 4-(3-cyanophenoxy)butanoate (6)

Parameter	Value
1 Title	Compound 6
2 Solvent	DMSO
3 Experiment	1D
4 Number of Scans	32

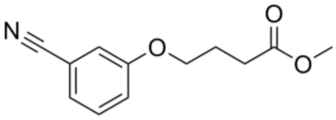

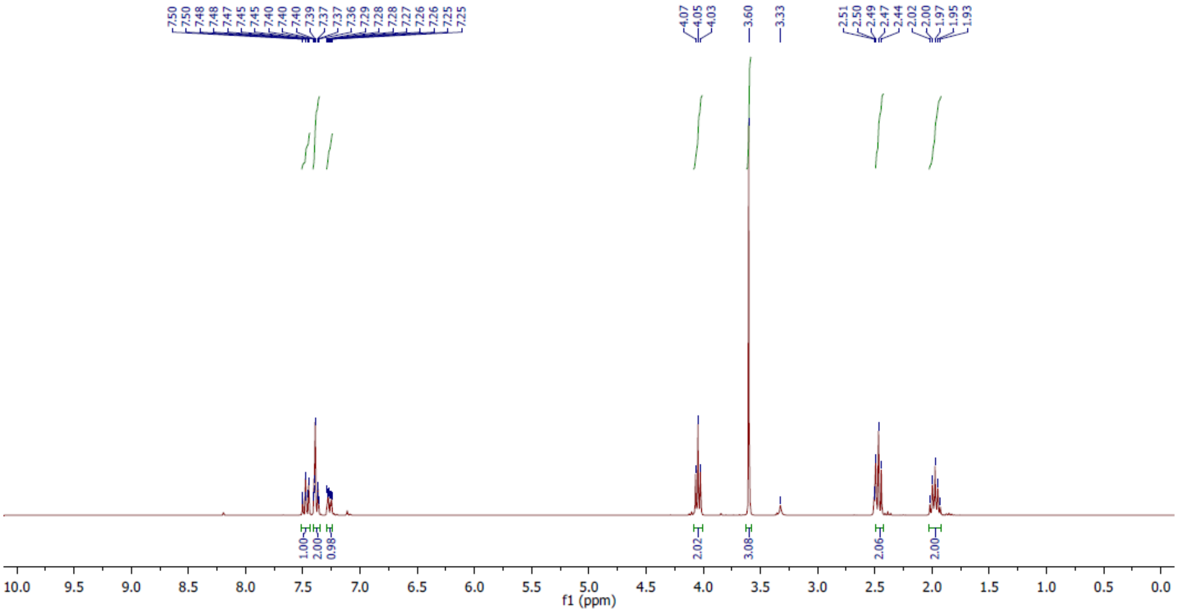

Parameter	Value
1 Title	Compound 6
2 Solvent	DMSO
3 Experiment	13C
4 Number of Scans	1024

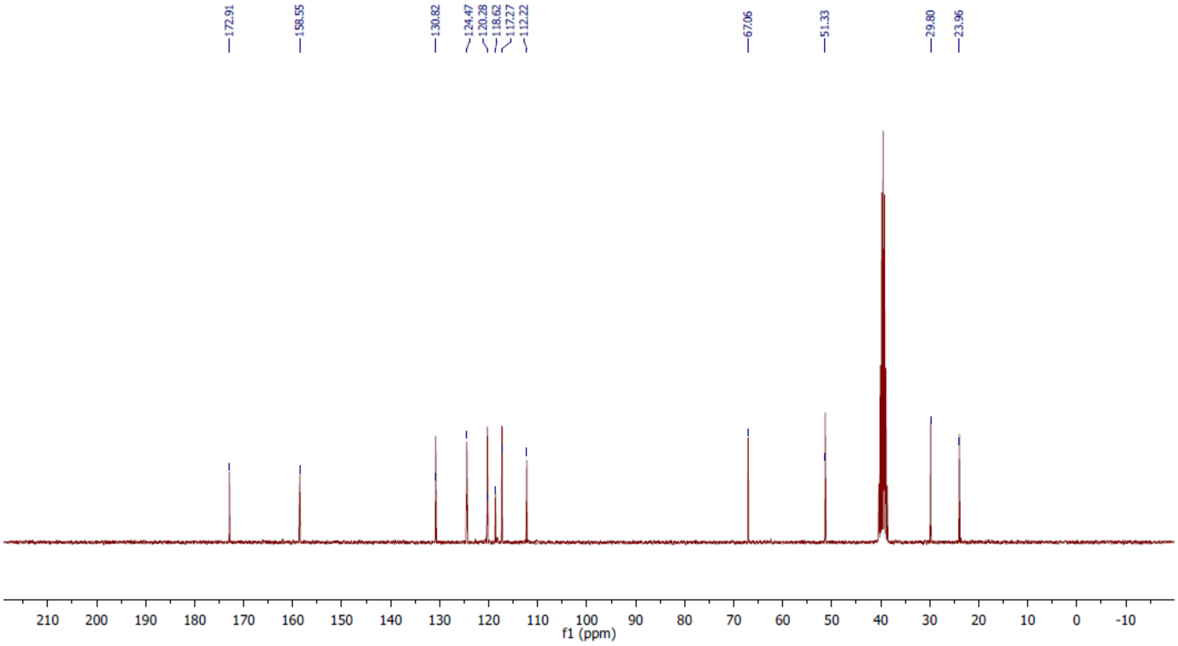

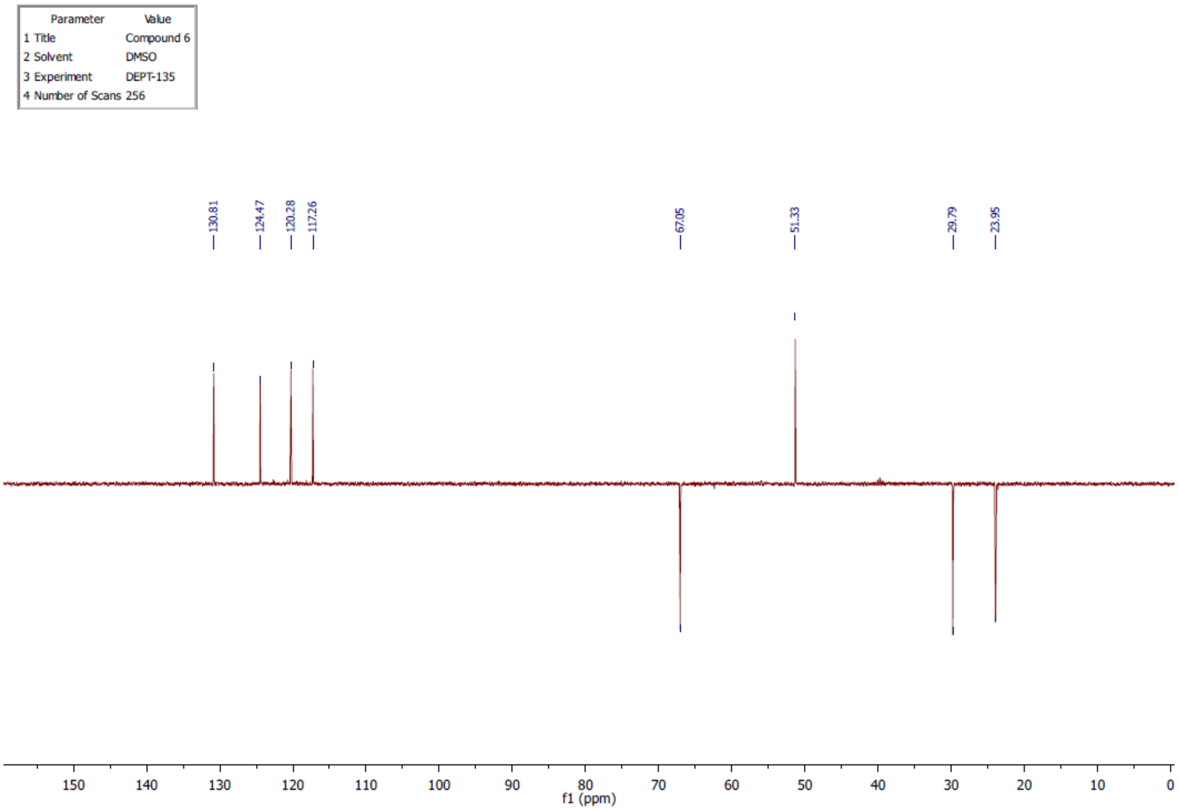
Parameter Value
1 Title Compound 6
2 Solvent DMSO
3 Experiment DEPT-135
4 Number of Scans 256
130.81
124.47
120.28
117.26
67.05
51.33
29.79
23.95
f1 (ppm)

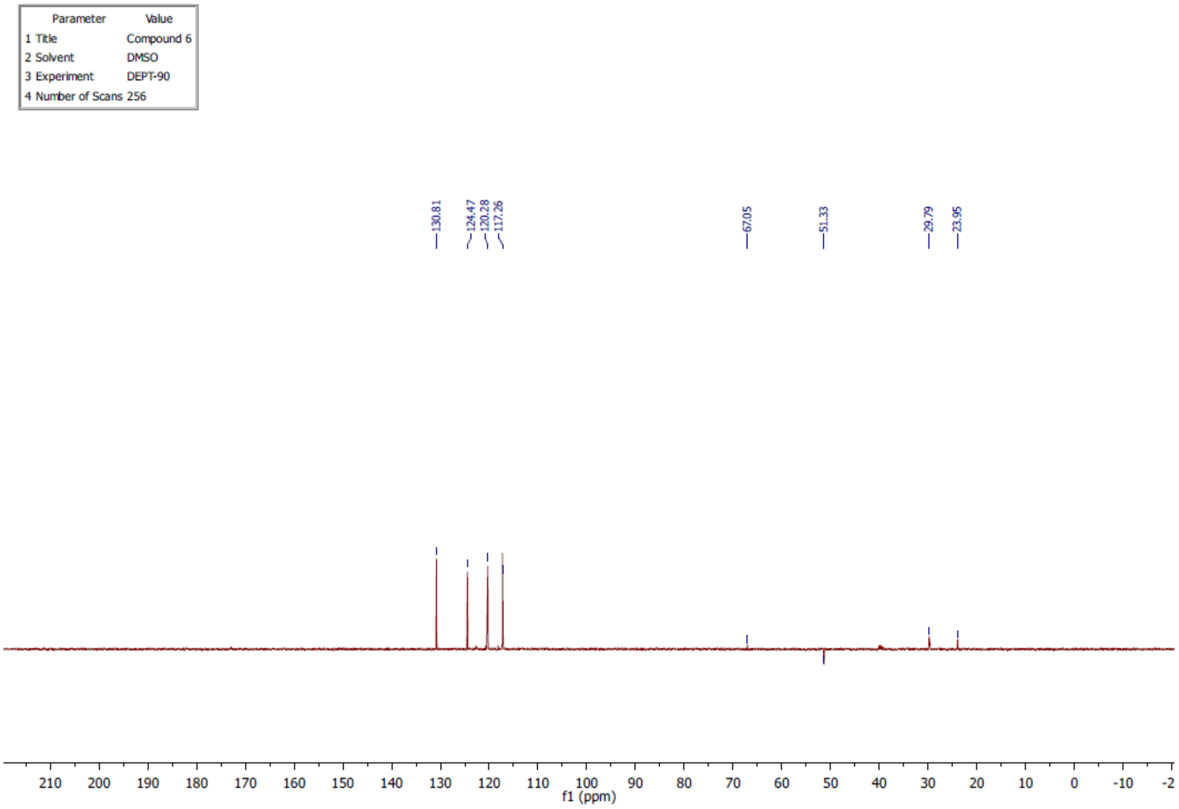
Parameter Value
1 Title Compound 6
2 Solvent DMSO
3 Experiment DEPT-90
4 Number of Scans 256
130.81
124.47
120.28
117.26
67.05
51.33
29.79
23.95
f1 (ppm)

Methyl 4-[3-(aminomethyl)phenoxy]butanoate (7f)

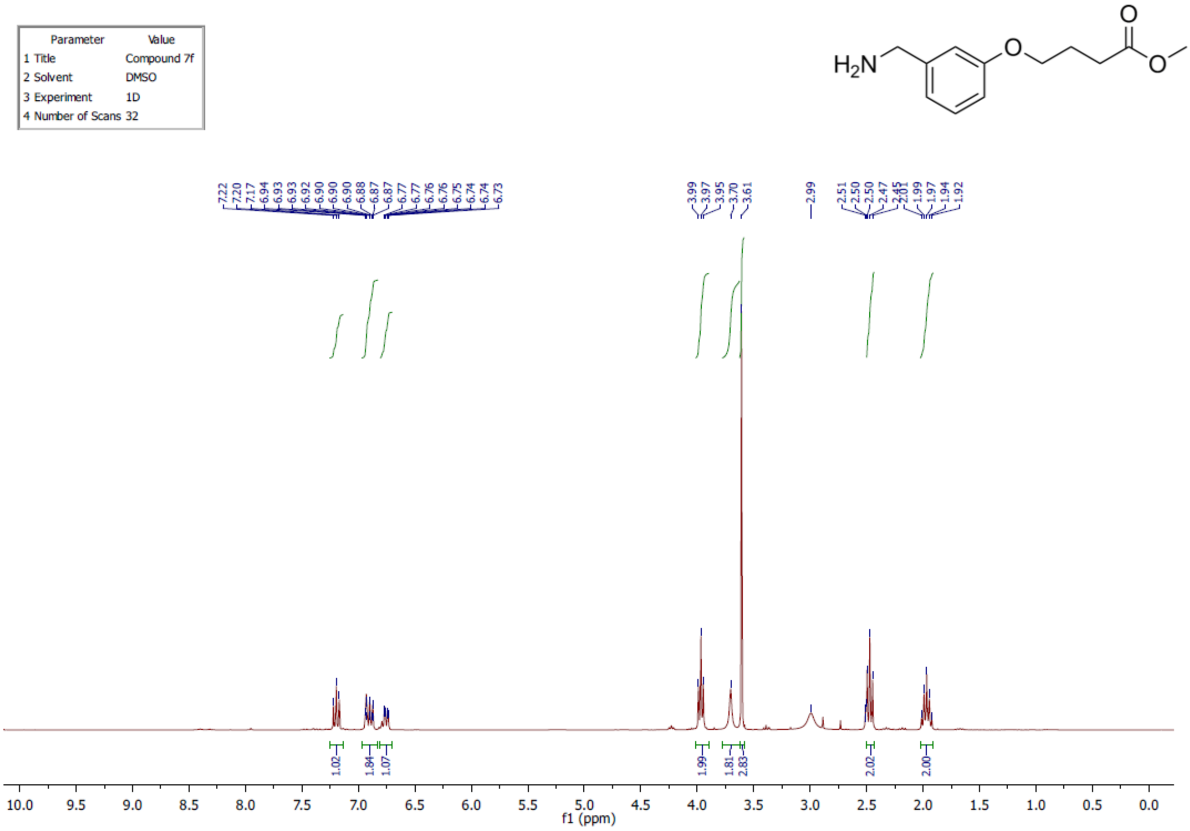

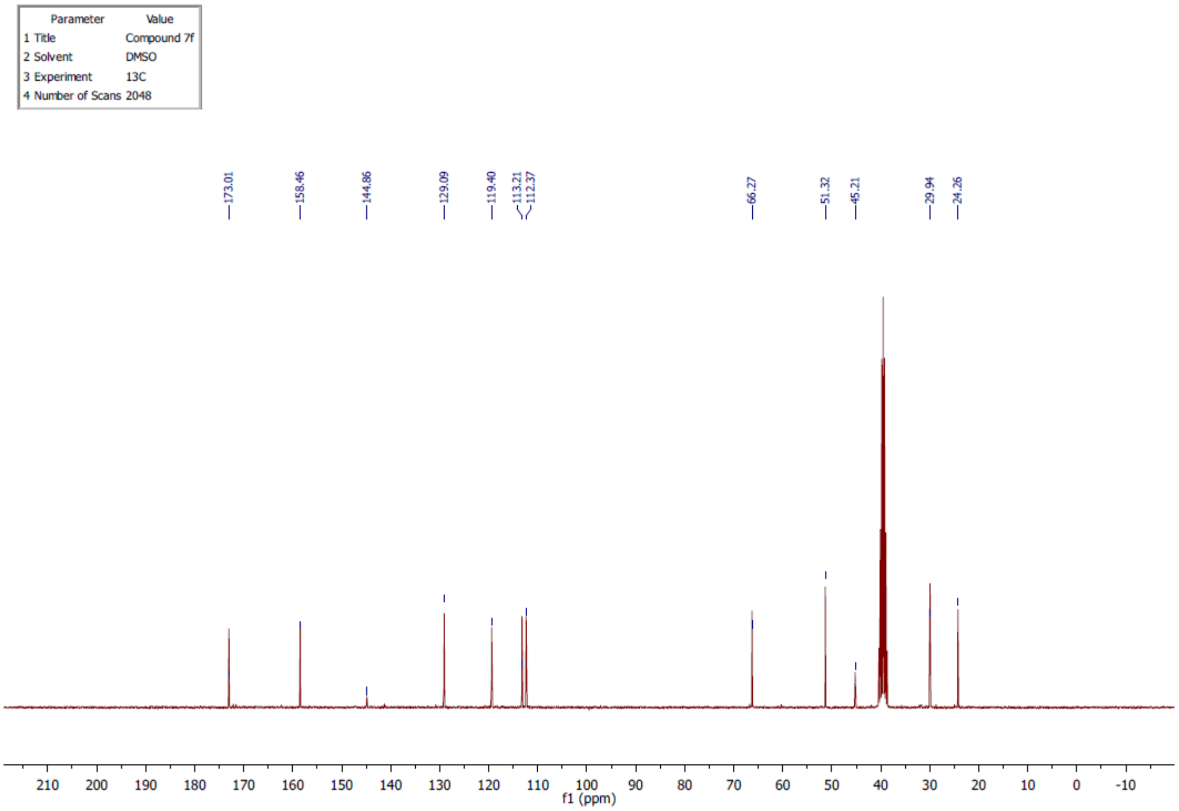

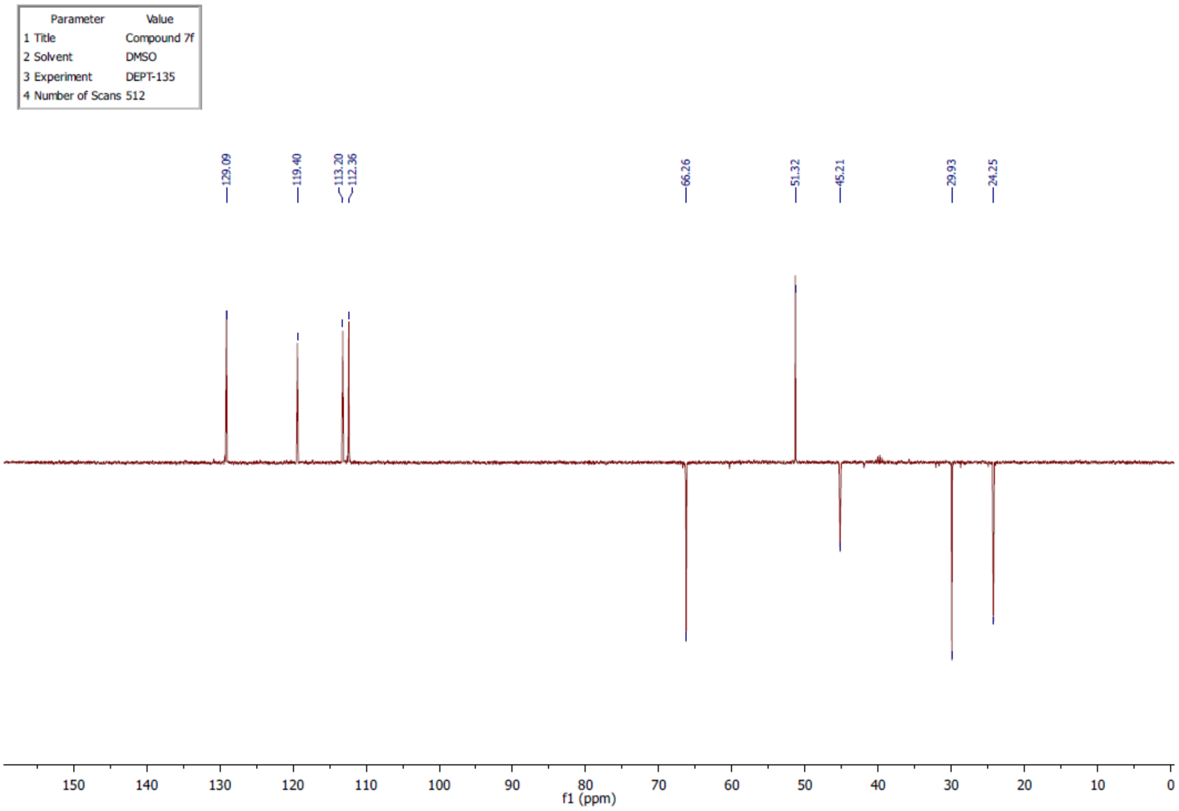
Parameter Value
1 Title Compound 7f
2 Solvent DMSO
3 Experiment DEPT-135
4 Number of Scans 512
129.09
119.40
113.20
112.36
66.26
51.32
45.21
29.93
24.25
f1 (ppm)

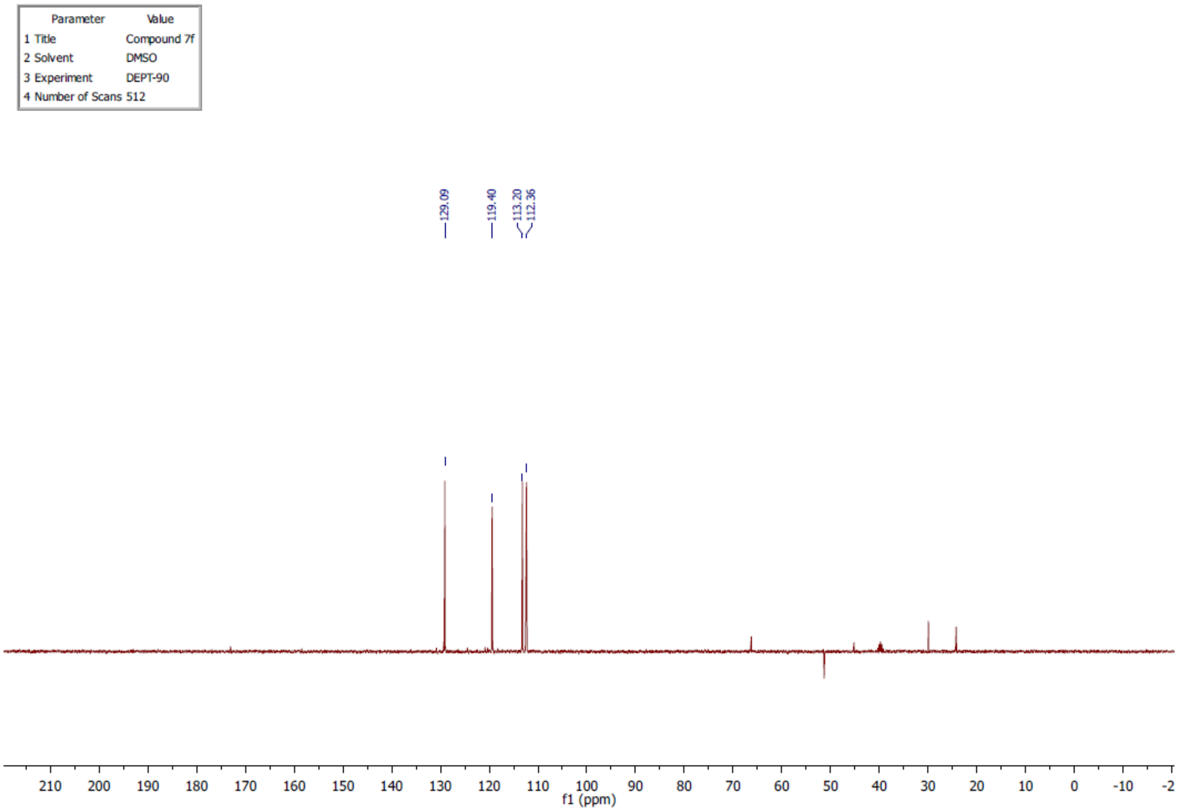
Parameter Value
1 Title Compound 7f
2 Solvent DMSO
3 Experiment DEPT-90
4 Number of Scans 512
129.09
119.40
113.20
112.36
f1 (ppm)

N-Benzylprop-2-ynamide (8a)

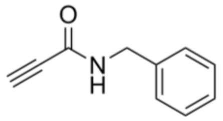

Parameter	Value
1 Title	Compound 8a
2 Solvent	DMSO
3 Experiment	1D
4 Number of Scans	16

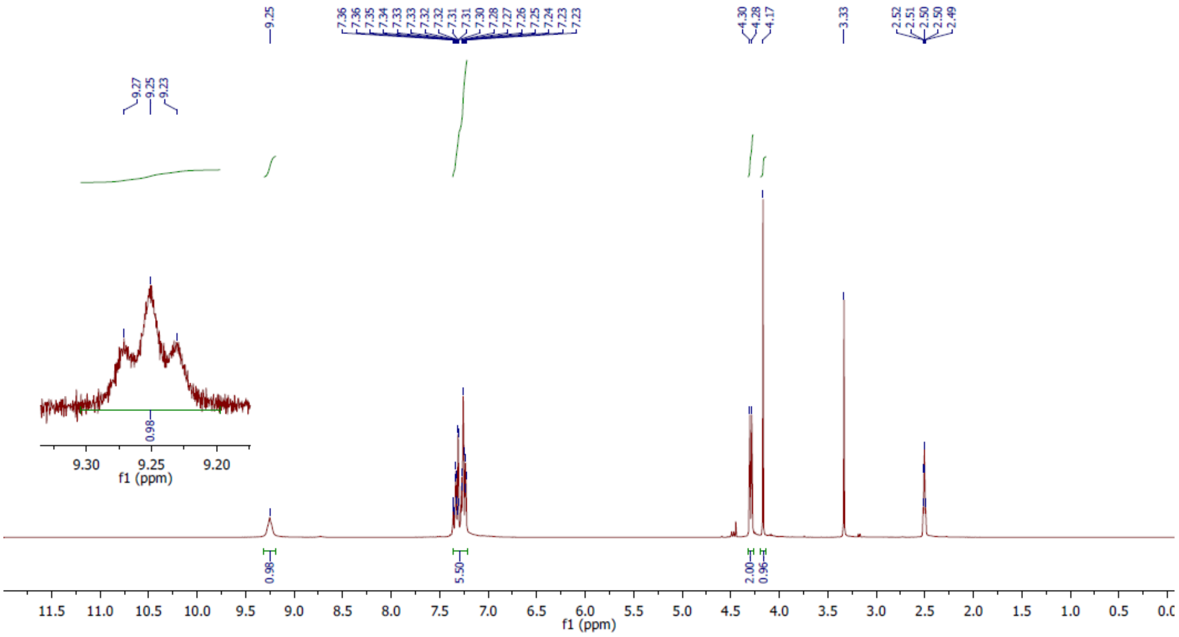

Parameter	Value
1 Title	Compound 8a
2 Solvent	DMSO
3 Experiment	13C
4 Number of Scans	2048

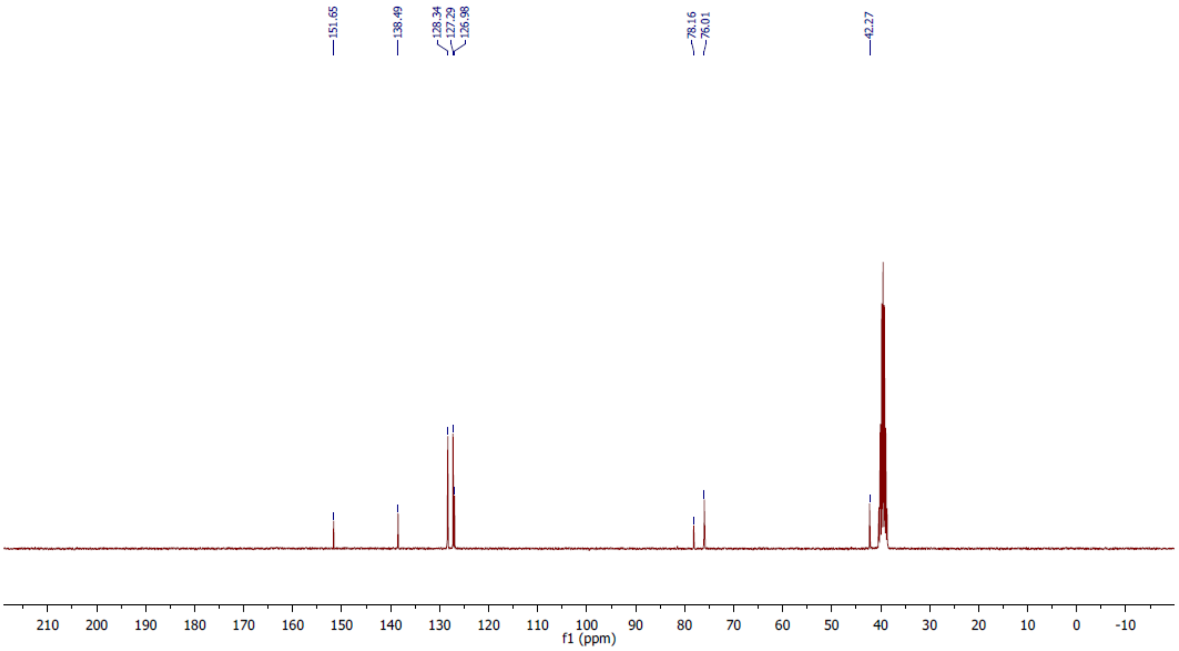

Parameter	Value
1 Title	Compound 8a
2 Solvent	DMSO
3 Experiment	DEPT-135
4 Number of Scans	512

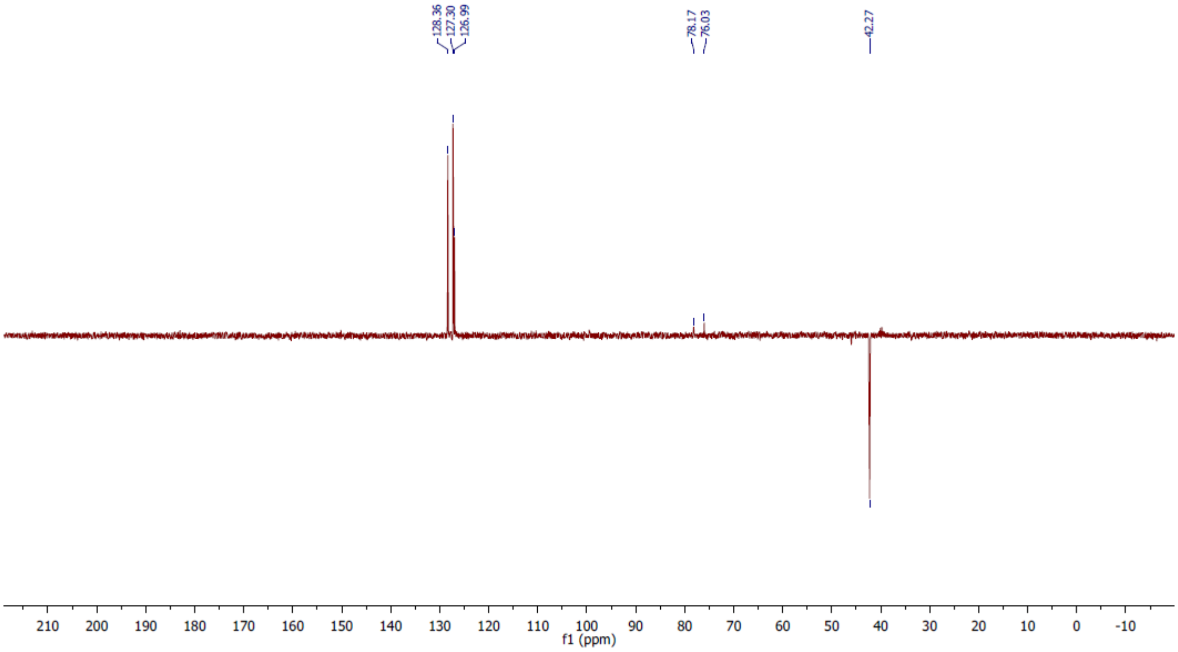

Parameter	Value
1 Title	Compound 8a
2 Solvent	DMSO
3 Experiment	DEPT-90
4 Number of Scans	512

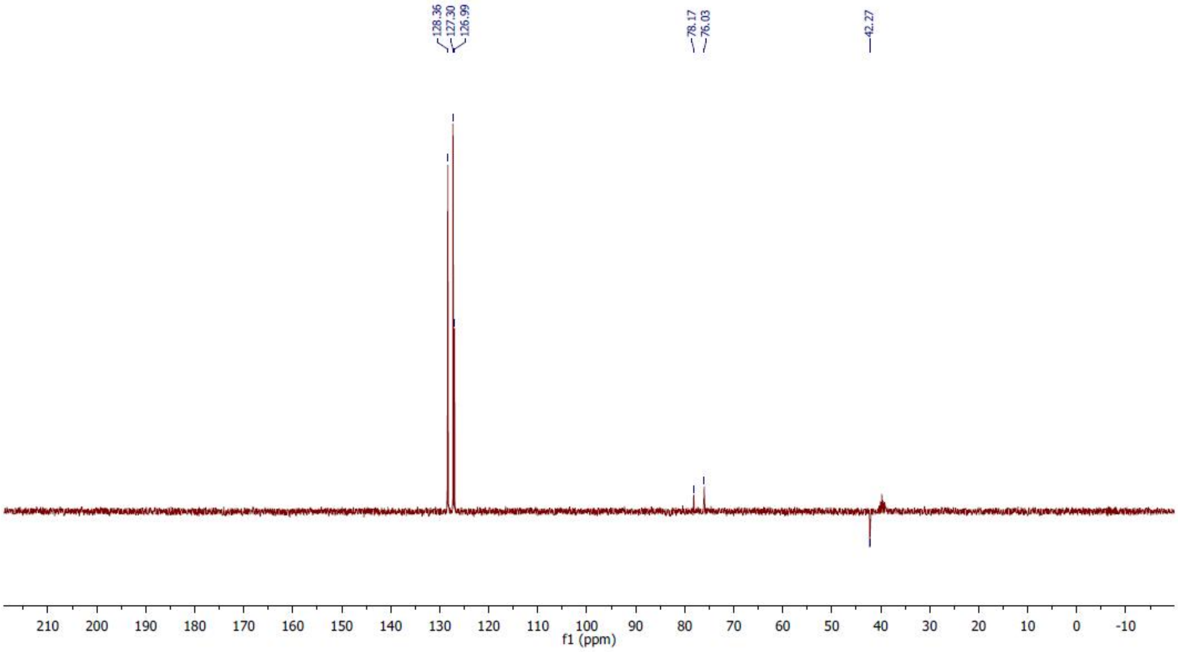

***N*-[(2-Fluorophenyl)methyl]prop-2-ynamide (8b)**

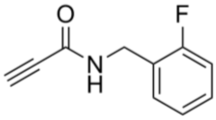

Parameter	Value
1 Title	Compound 8b
2 Solvent	DMSO
3 Experiment	1D
4 Number of Scans	32

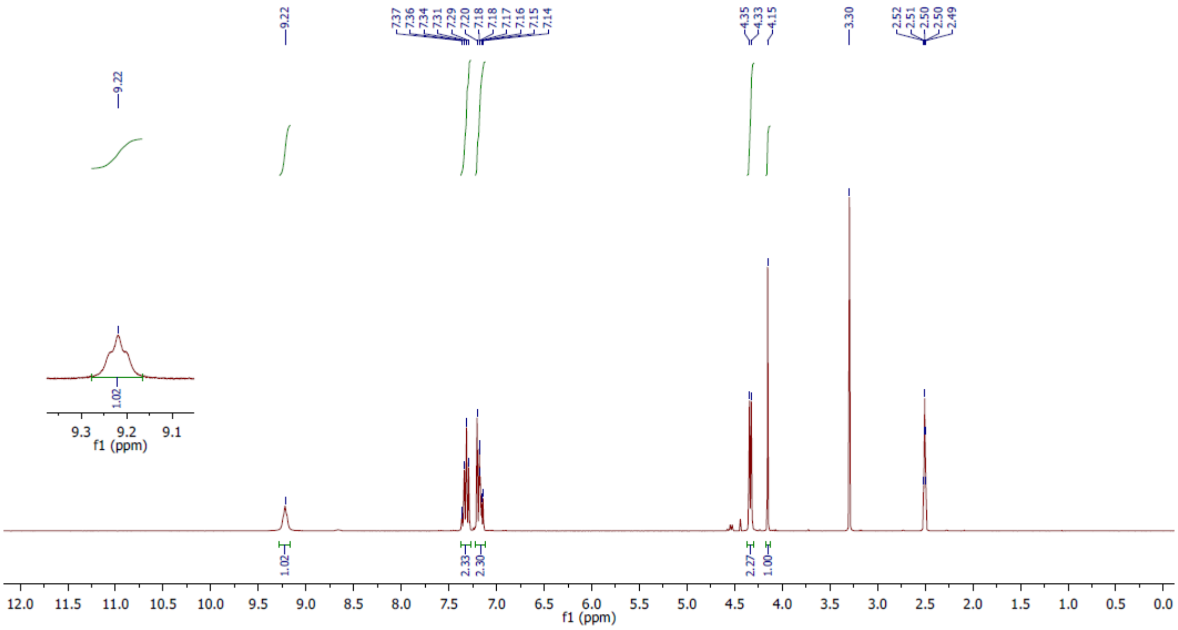

Parameter	Value
1 Title	Compound 8b
2 Solvent	DMSO
3 Experiment	13C
4 Number of Scans	2048

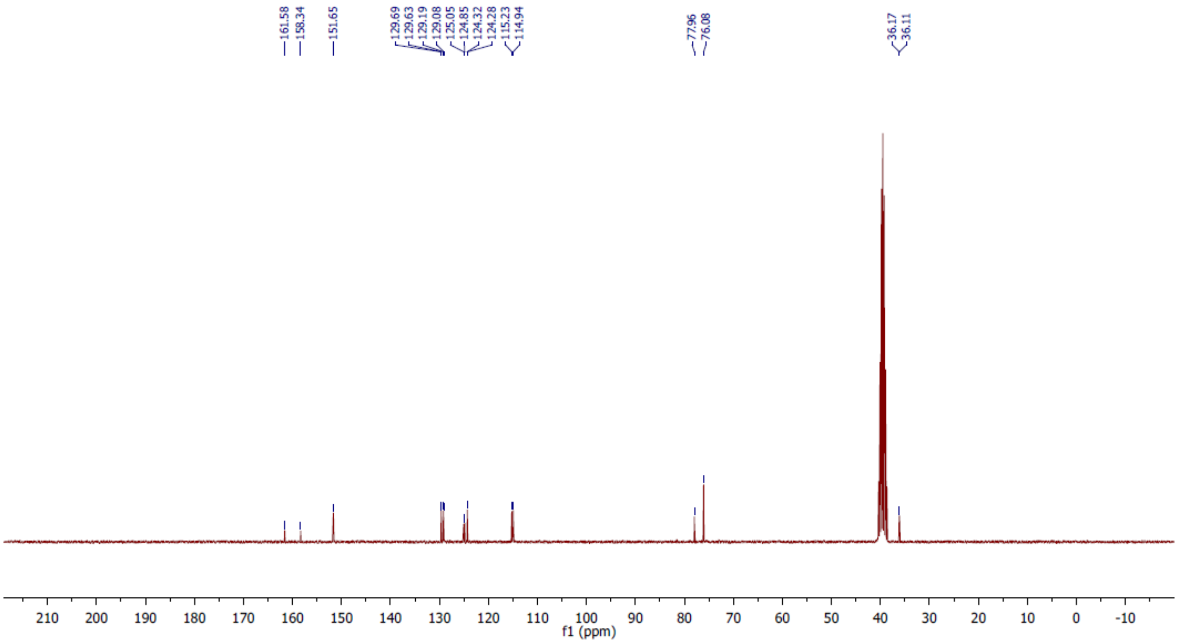

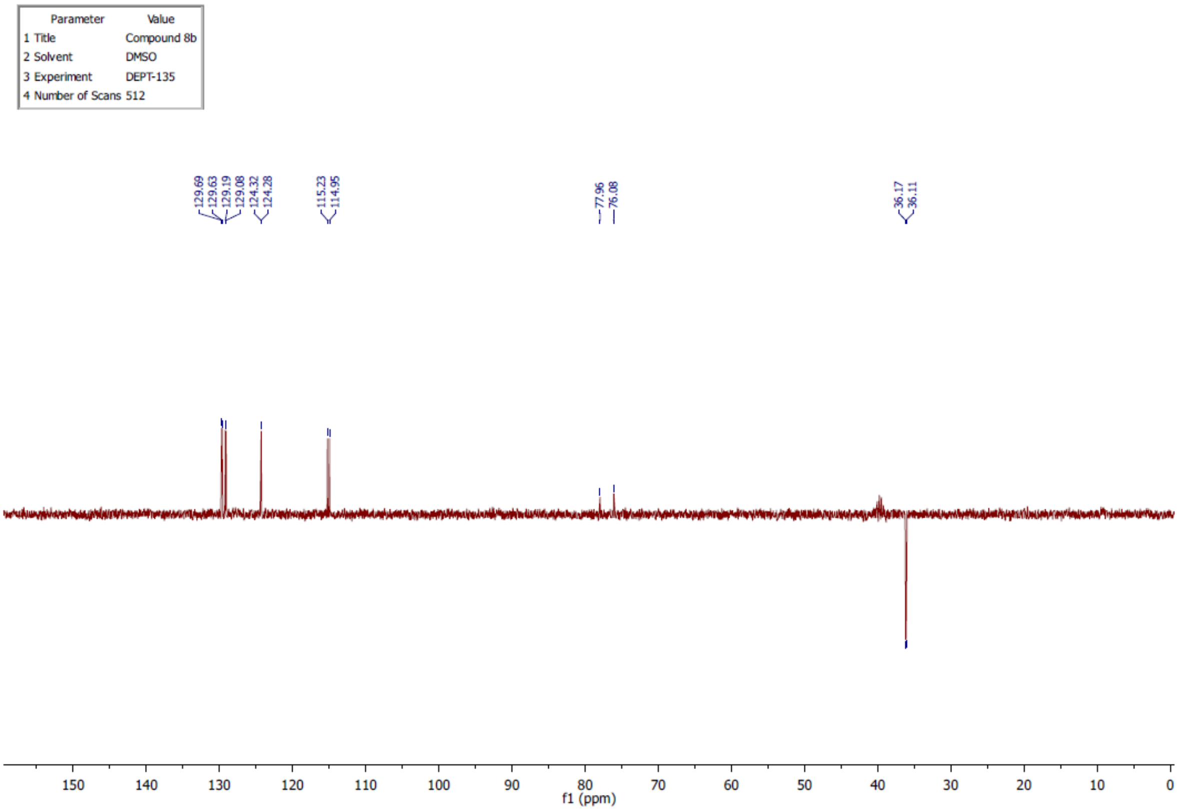
Parameter Value
1 Title Compound 8b
2 Solvent DMSO
3 Experiment DEPT-135
4 Number of Scans 512
129.69
129.63
129.19
129.08
124.32
124.28
115.23
114.95
77.96
76.08
36.17
36.11
f1 (ppm)

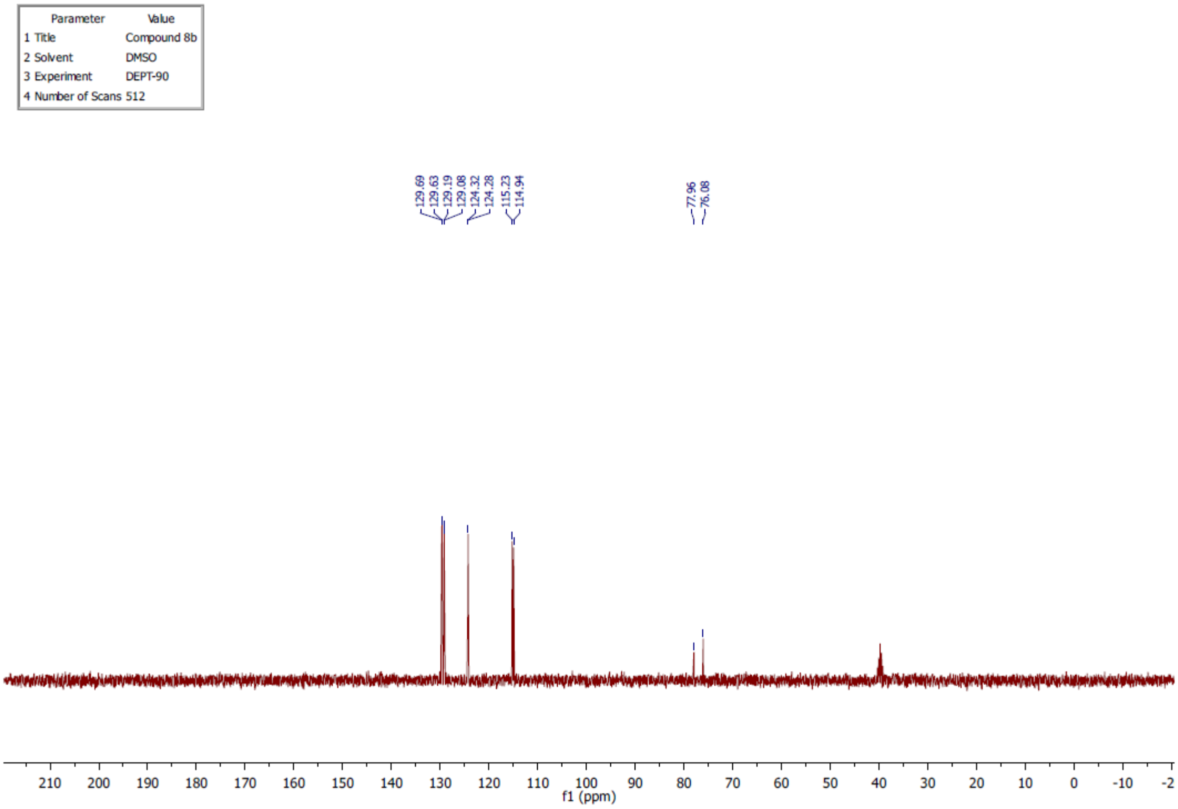
Parameter Value
1 Title Compound 8b
2 Solvent DMSO
3 Experiment DEPT-90
4 Number of Scans 512
129.69
129.63
129.19
129.08
124.32
124.28
115.23
114.94
77.96
76.08
f1 (ppm)

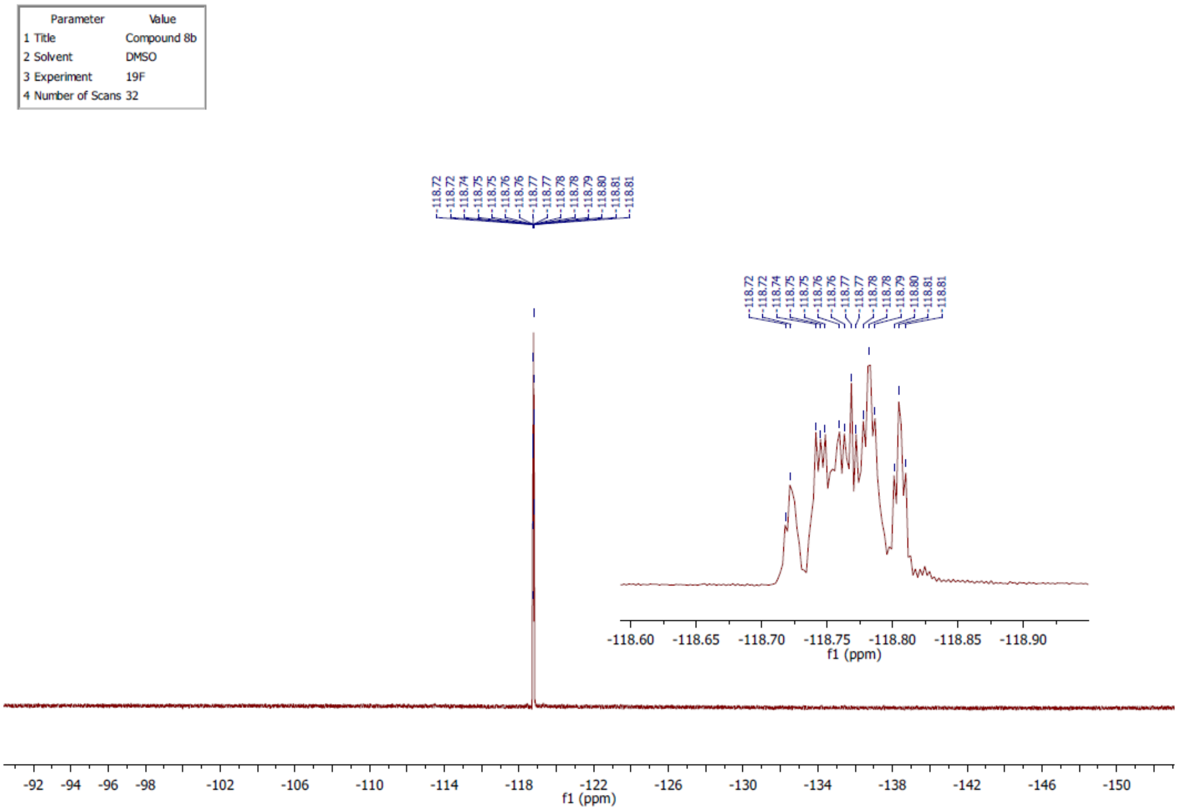

N-[(3-Fluorophenyl)methyl]prop-2-ynamide (8c)

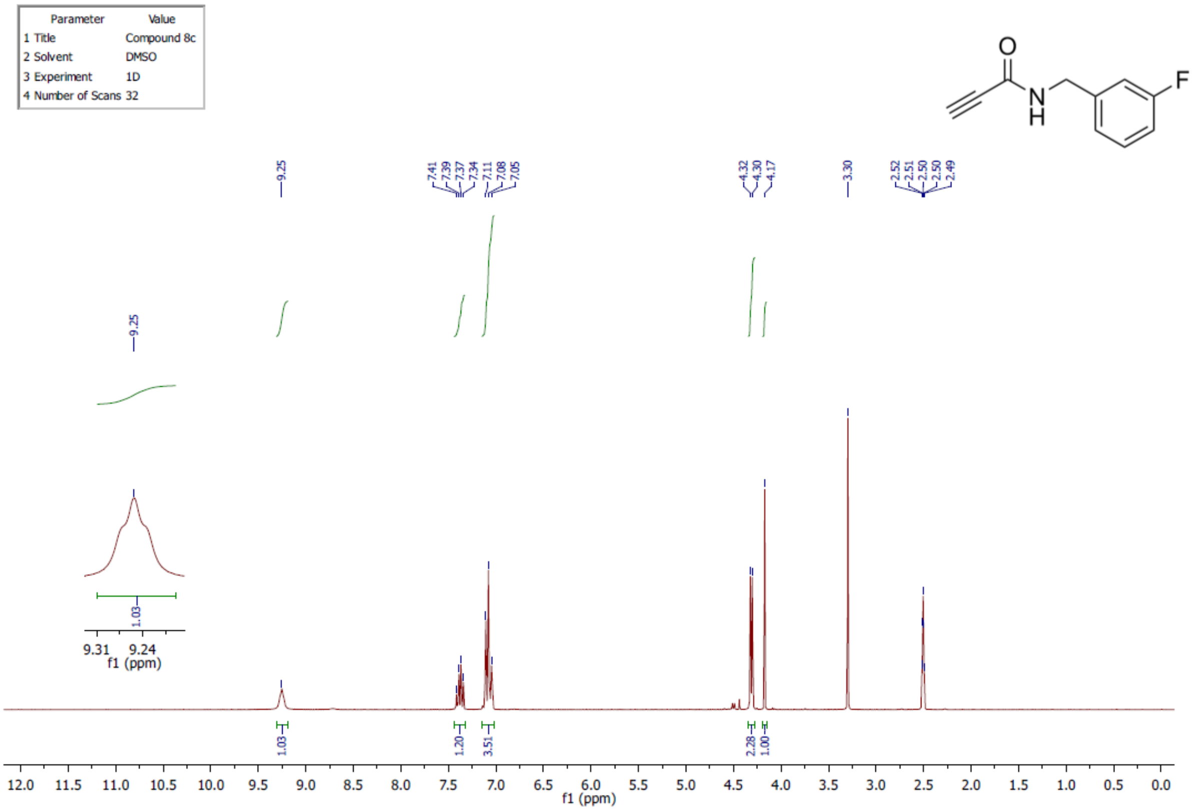

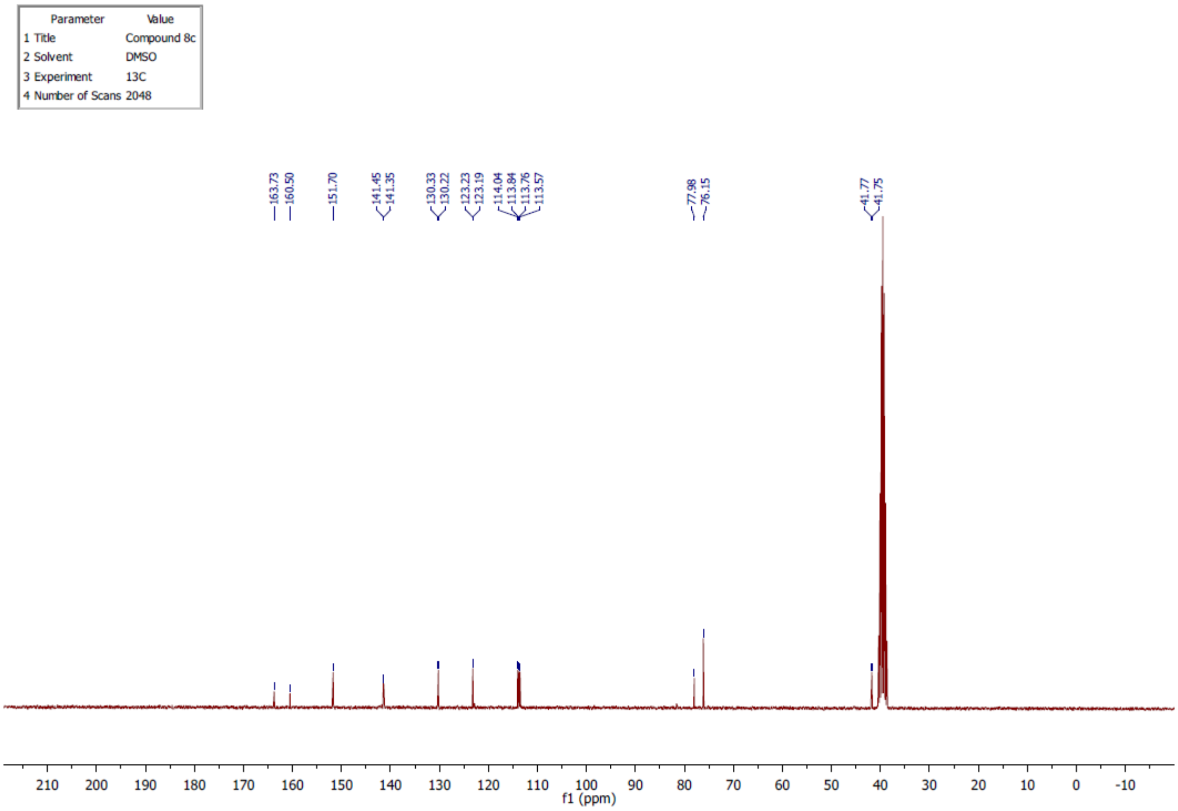
Parameter Value
1 Title Compound 8c
2 Solvent DMSO
3 Experiment 13C
4 Number of Scans 2048
163.73
160.50
151.70
141.45
141.35
130.33
130.22
123.23
123.19
114.04
113.84
113.76
113.57
77.98
76.15
41.77
41.75
210 200 190 180 170 160 150 140 130 120 110 100 90 80 70 60 50 40 30 20 10 0 -10
f1 (ppm)

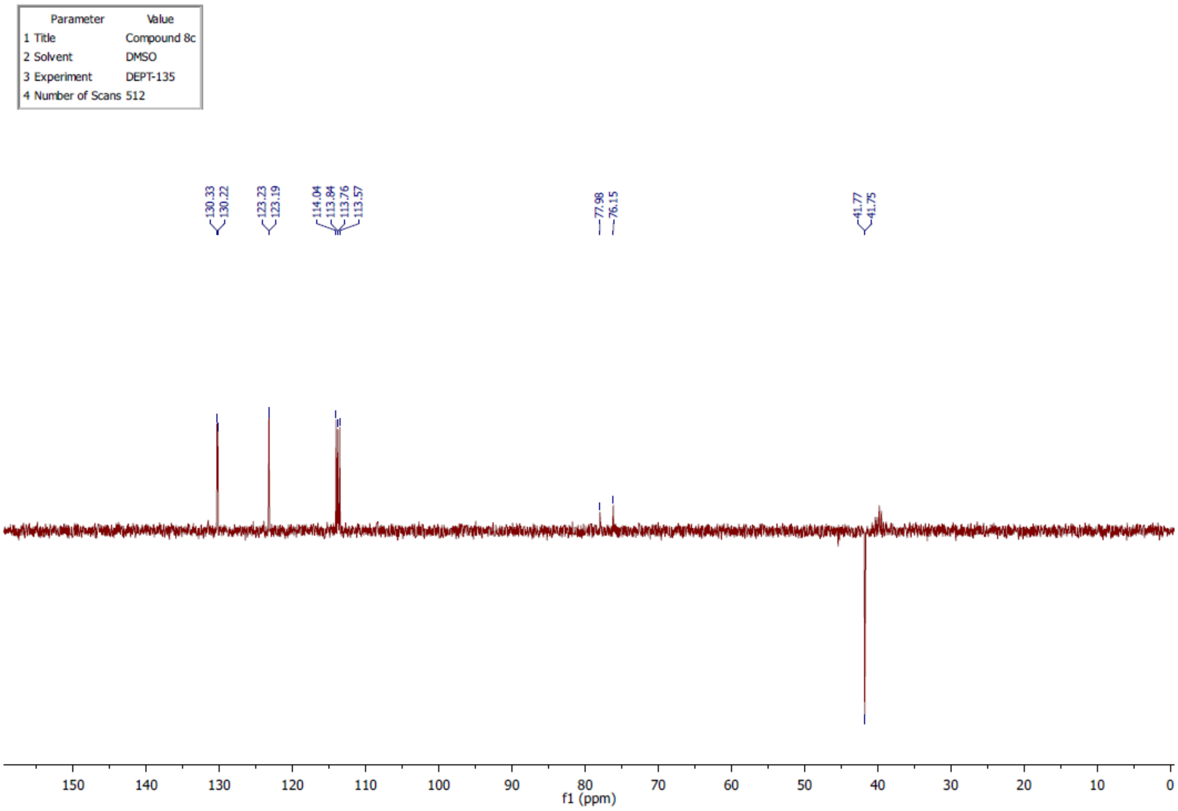
Parameter Value
1 Title Compound 8c
2 Solvent DMSO
3 Experiment DEPT-135
4 Number of Scans 512
130.33
130.22
123.23
123.19
114.04
113.84
113.76
113.57
77.98
76.15
41.77
41.75
150 140 130 120 110 100 90 80 70 60 50 40 30 20 10 0
f1 (ppm)

Parameter	Value
1 Title	Compound 8c
2 Solvent	DMSO
3 Experiment	DEPT-90
4 Number of Scans	512

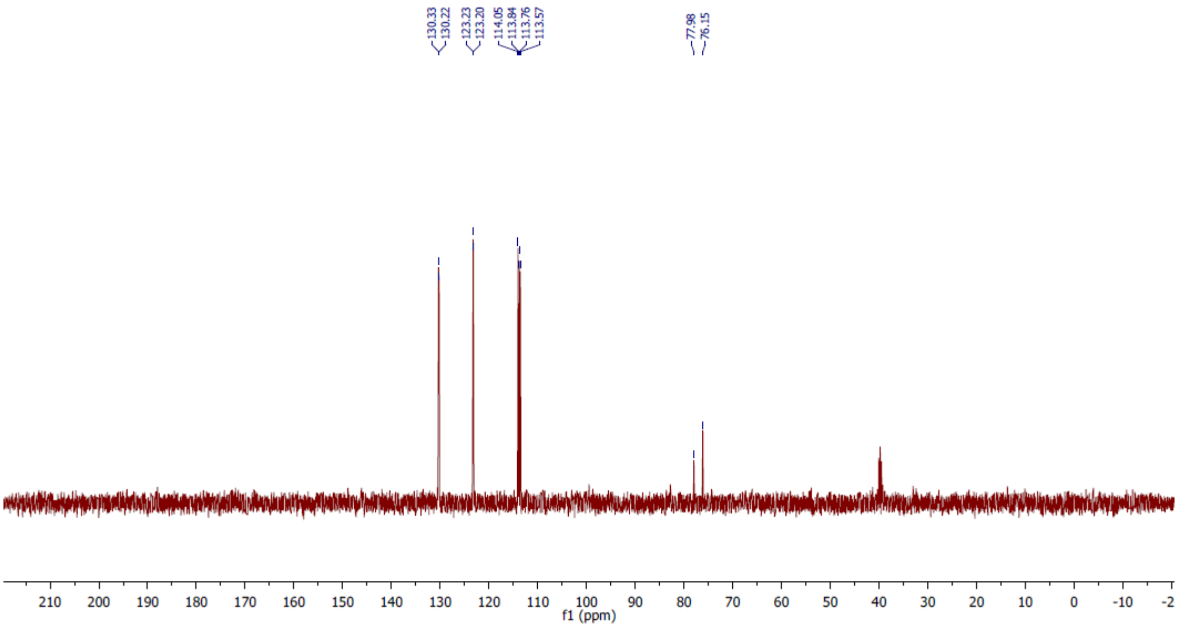

Parameter	Value
1 Title	Compound 8c
2 Solvent	DMSO
3 Experiment	19F
4 Number of Scans	32

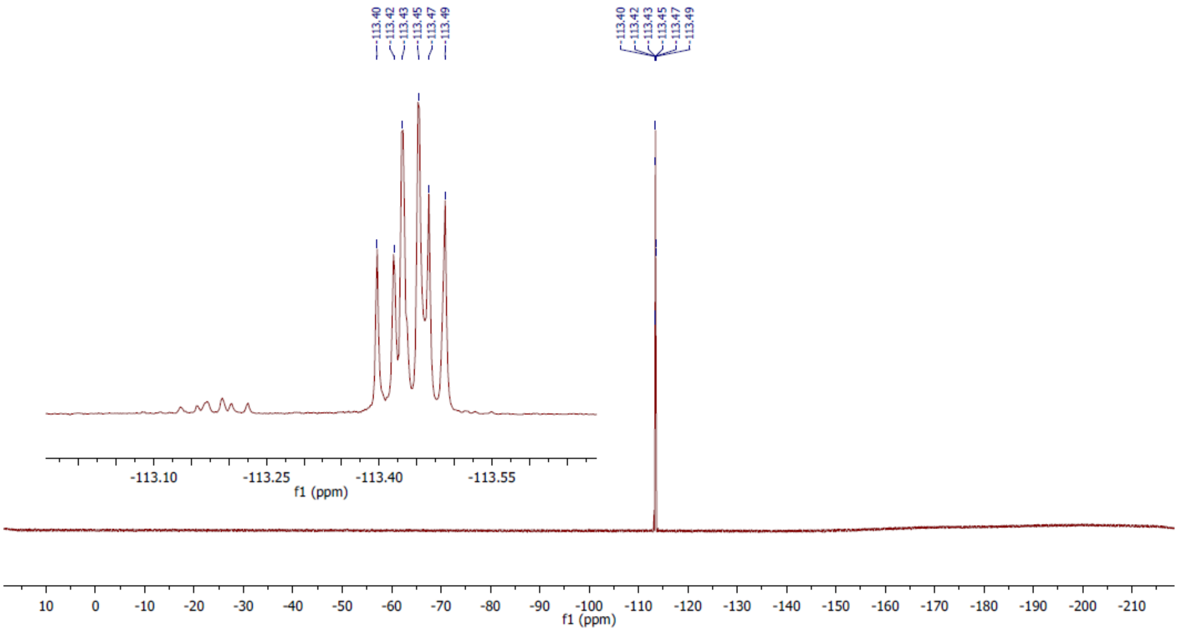

N-[(4-Fluorophenyl)methyl]prop-2-ynamide (8d)

Parameter	Value
1 Title	Compound 8d
2 Solvent	DMSO
3 Experiment	1D
4 Number of Scans	32

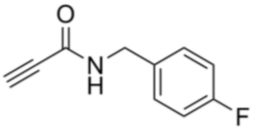

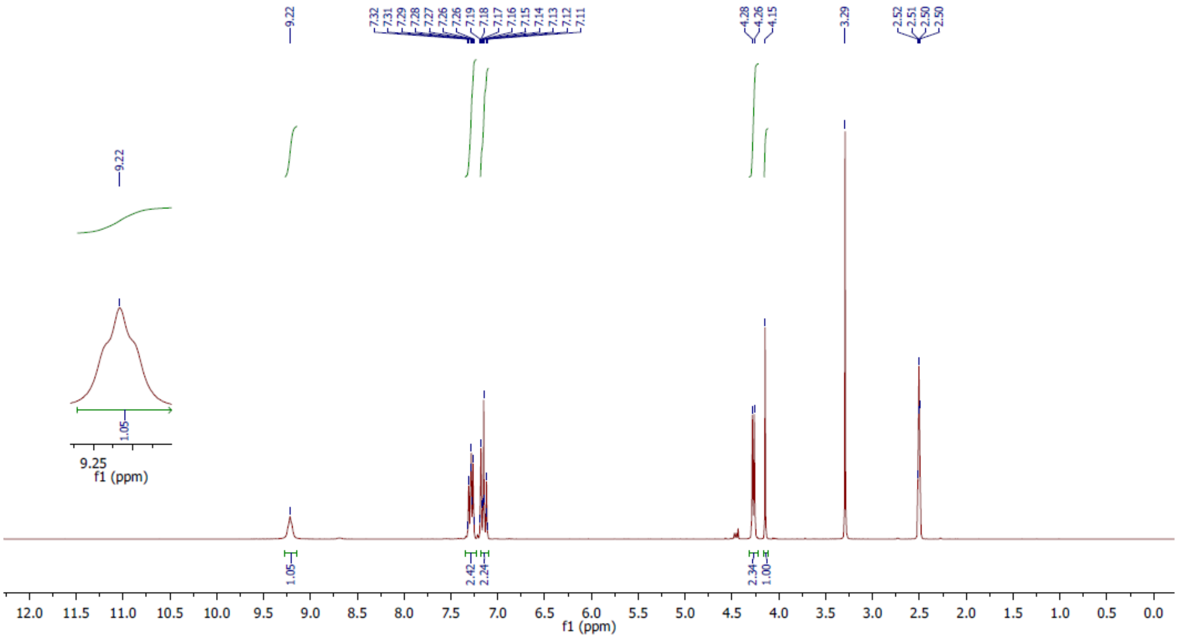

Parameter	Value
1 Title	Compound 8d
2 Solvent	DMSO
3 Experiment	13C
4 Number of Scans	2048

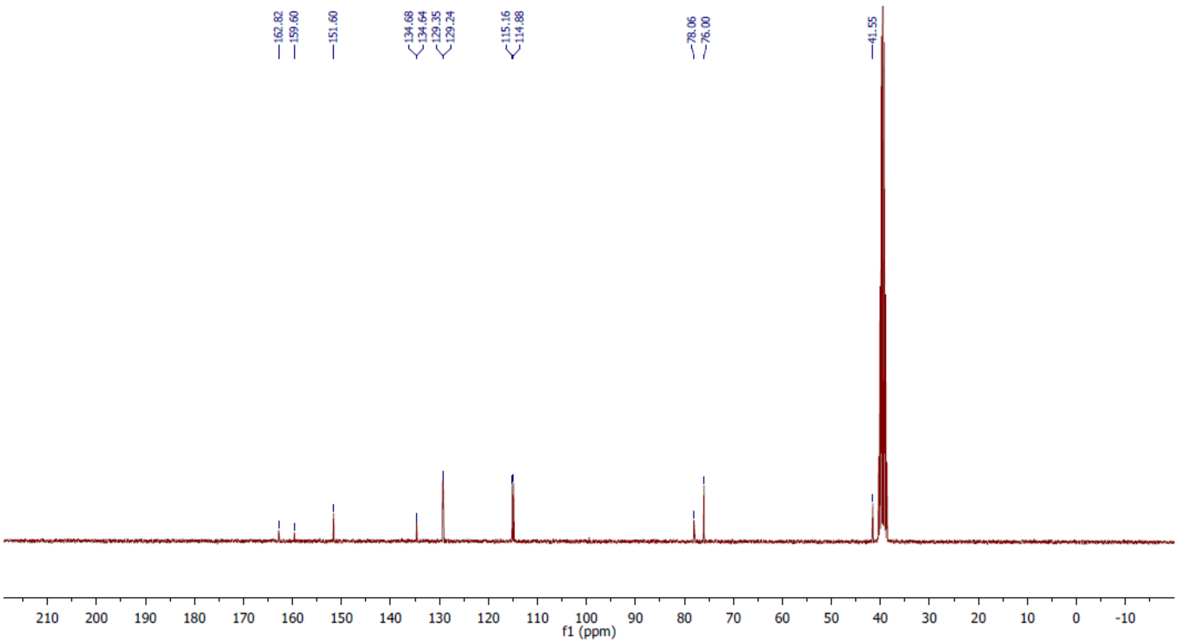

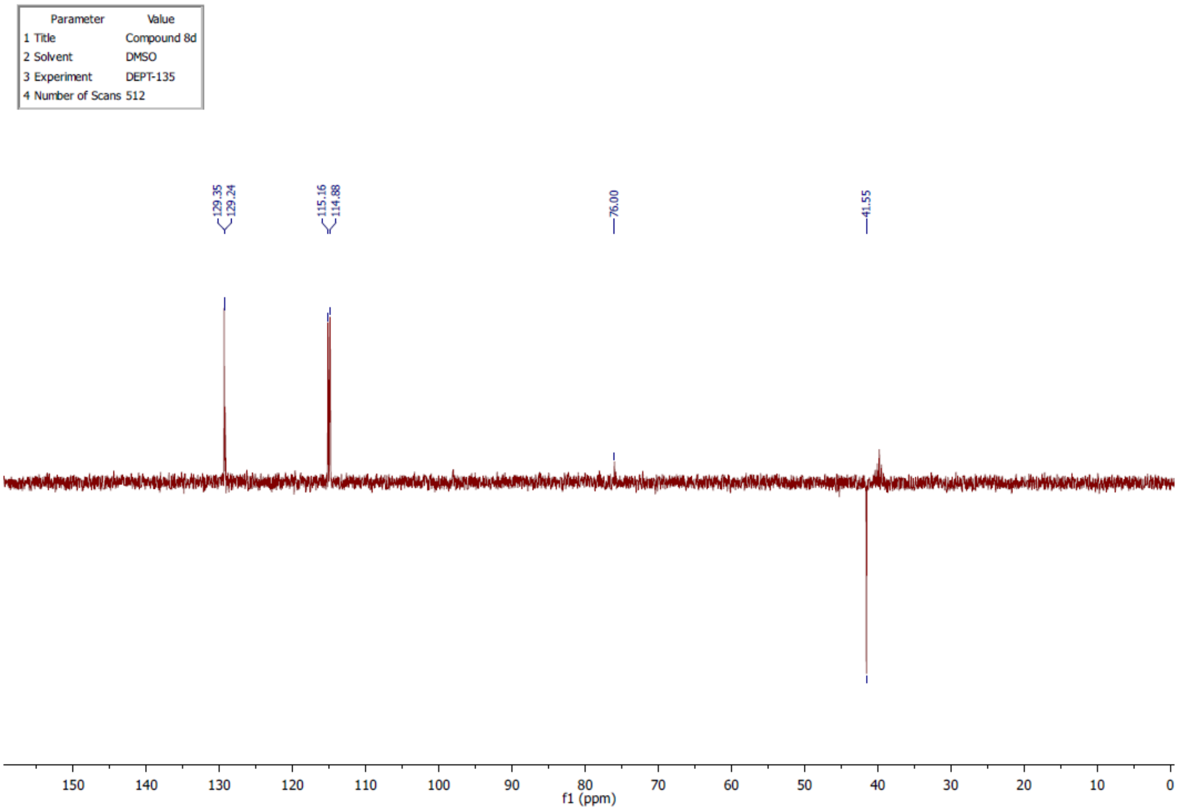
Parameter Value
1 Title Compound 8d
2 Solvent DMSO
3 Experiment DEPT-135
4 Number of Scans 512
129.35
129.24
115.16
114.88
76.00
41.55
150 140 130 120 110 100 90 80 70 60 50 40 30 20 10 0
f1 (ppm)

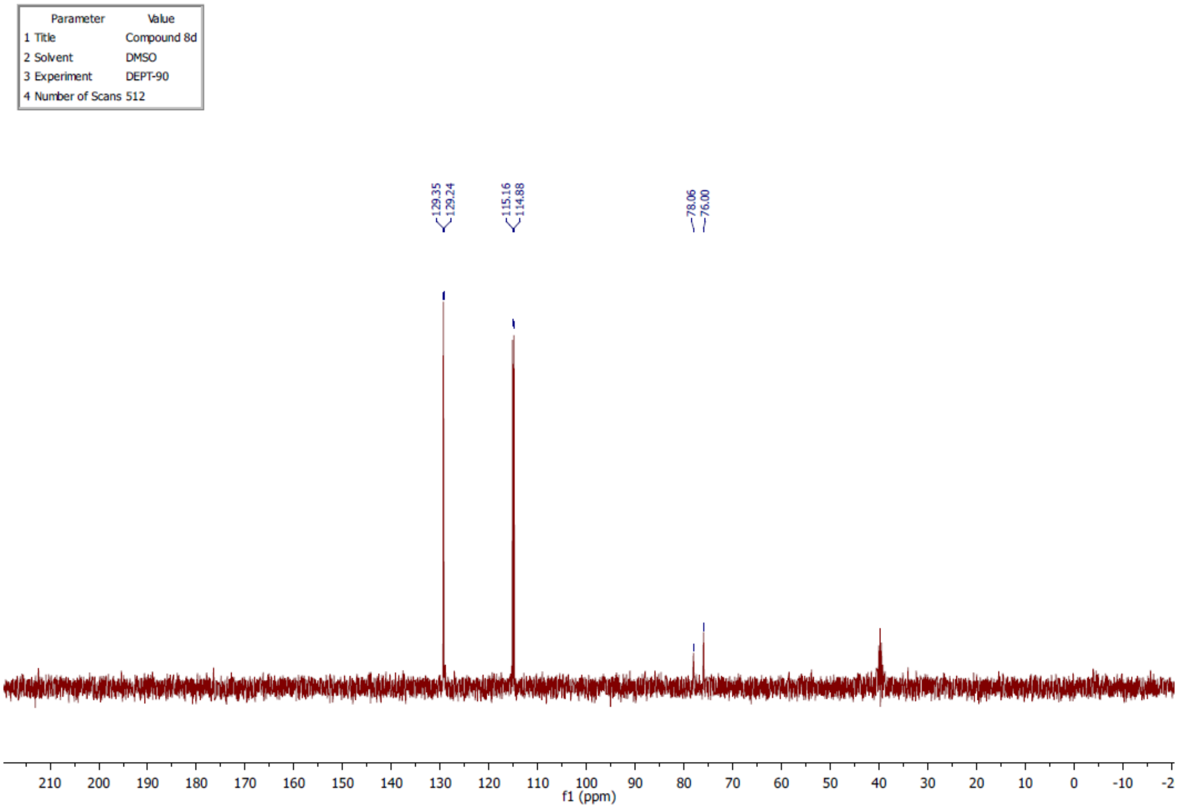
Parameter Value
1 Title Compound 8d
2 Solvent DMSO
3 Experiment DEPT-90
4 Number of Scans 512
129.35
129.24
115.16
114.88
78.06
76.00
210 200 190 180 170 160 150 140 130 120 110 100 90 80 70 60 50 40 30 20 10 0 -10 -2
f1 (ppm)

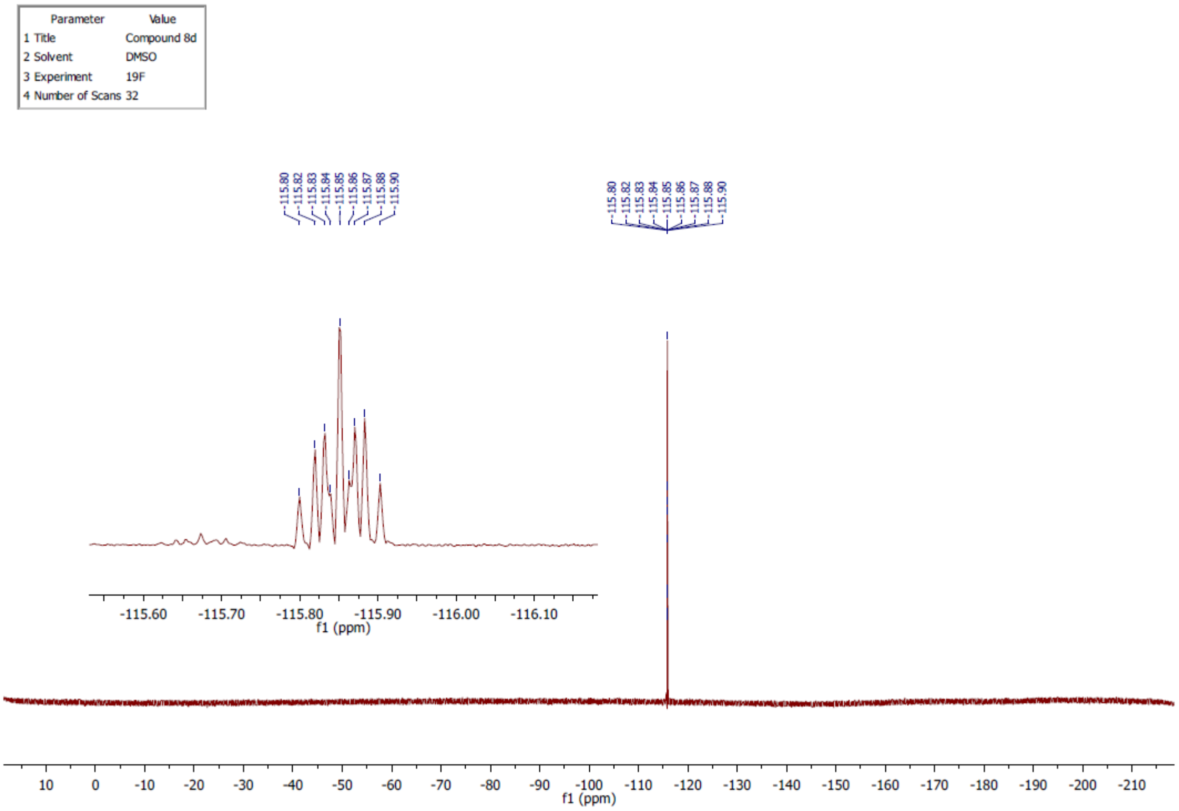

N-[(3-Methoxyphenyl)methyl]prop-2-ynamide (8e)

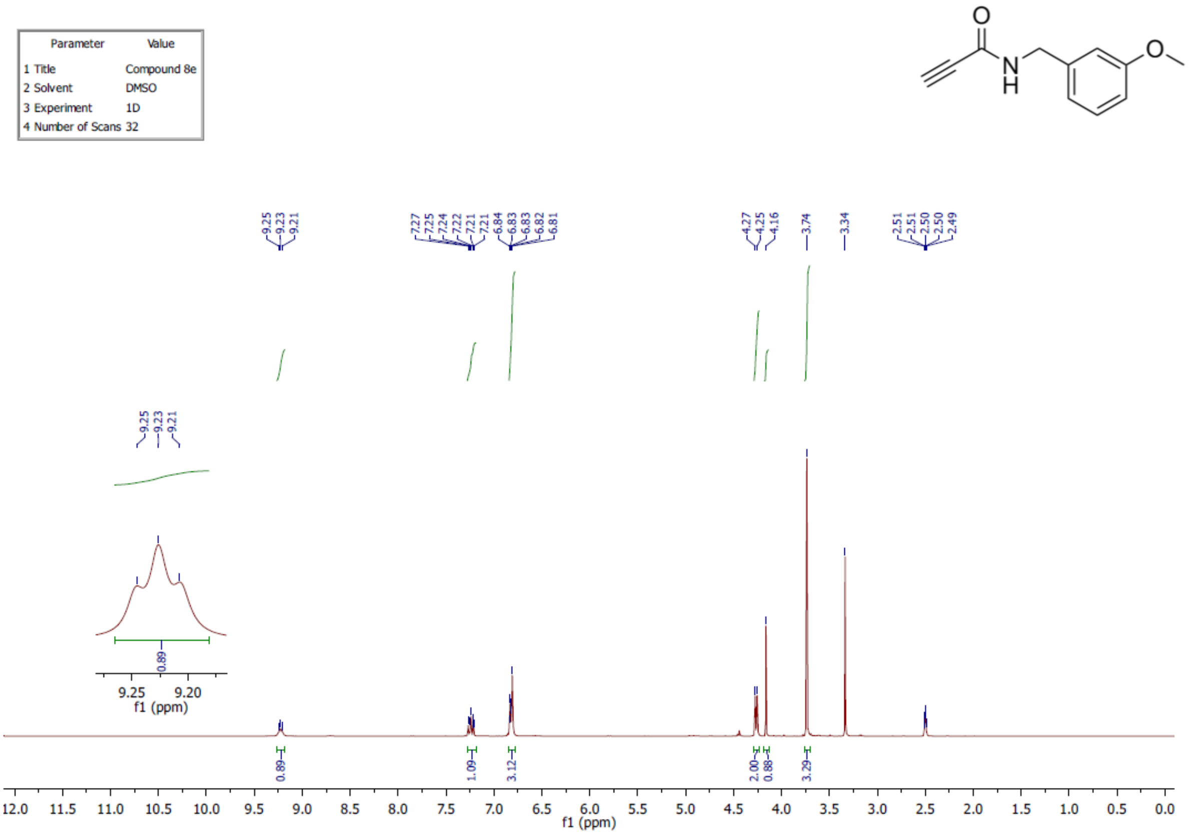

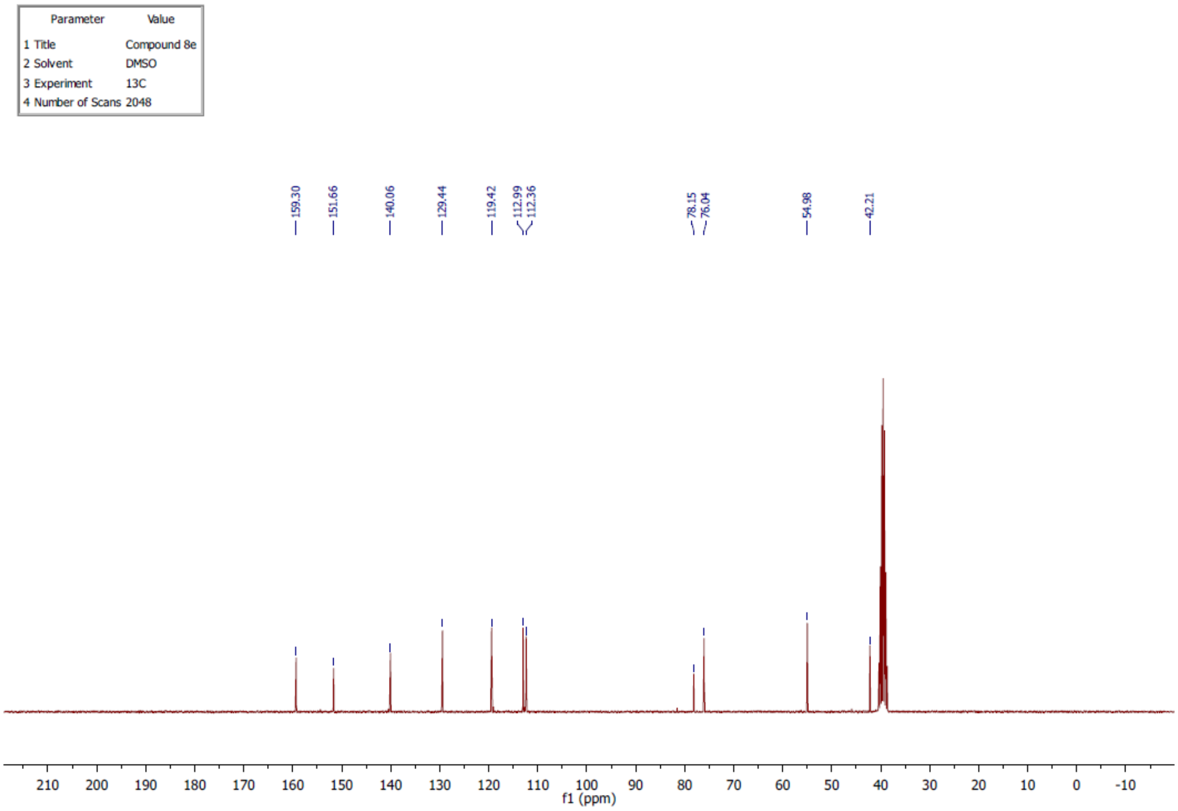
Parameter Value
1 Title Compound 8e
2 Solvent DMSO
3 Experiment 13C
4 Number of Scans 2048
159.30
151.66
140.06
129.44
119.42
112.99
112.36
78.15
76.04
54.98
42.21
210 200 190 180 170 160 150 140 130 120 110 100 90 80 70 60 50 40 30 20 10 0 -10
f1 (ppm)

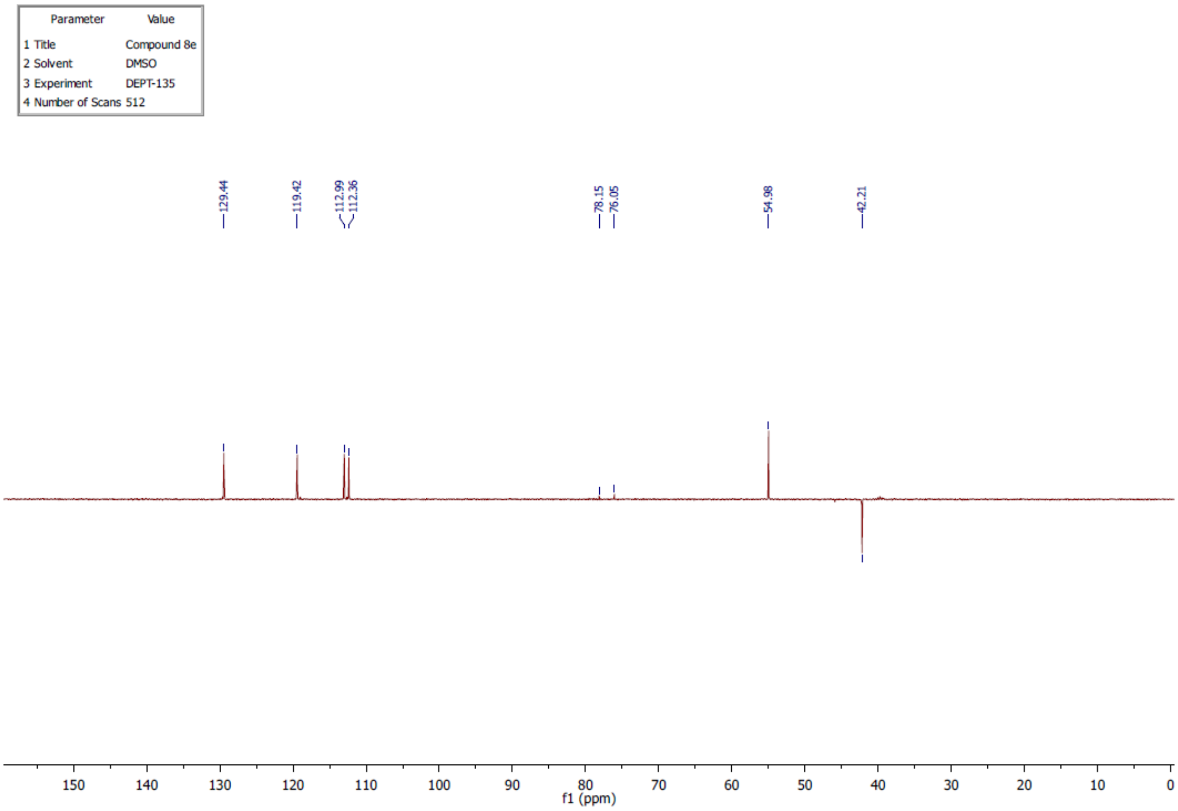
Parameter Value
1 Title Compound 8e
2 Solvent DMSO
3 Experiment DEPT-135
4 Number of Scans 512
129.44
119.42
112.99
112.36
78.15
76.05
54.98
42.21
150 140 130 120 110 100 90 80 70 60 50 40 30 20 10 0
f1 (ppm)

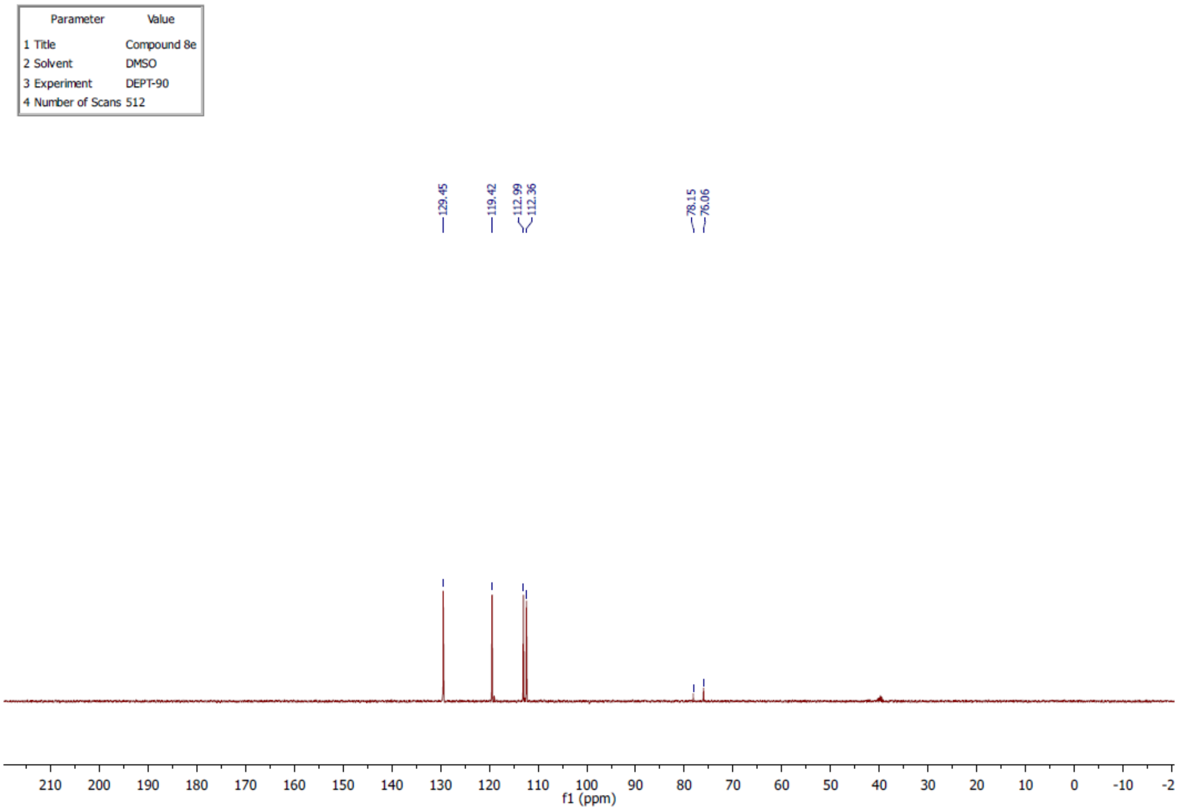

Methyl 4-[3-(prop-2-ynamidomethyl)phenoxy]butanoate (8f)

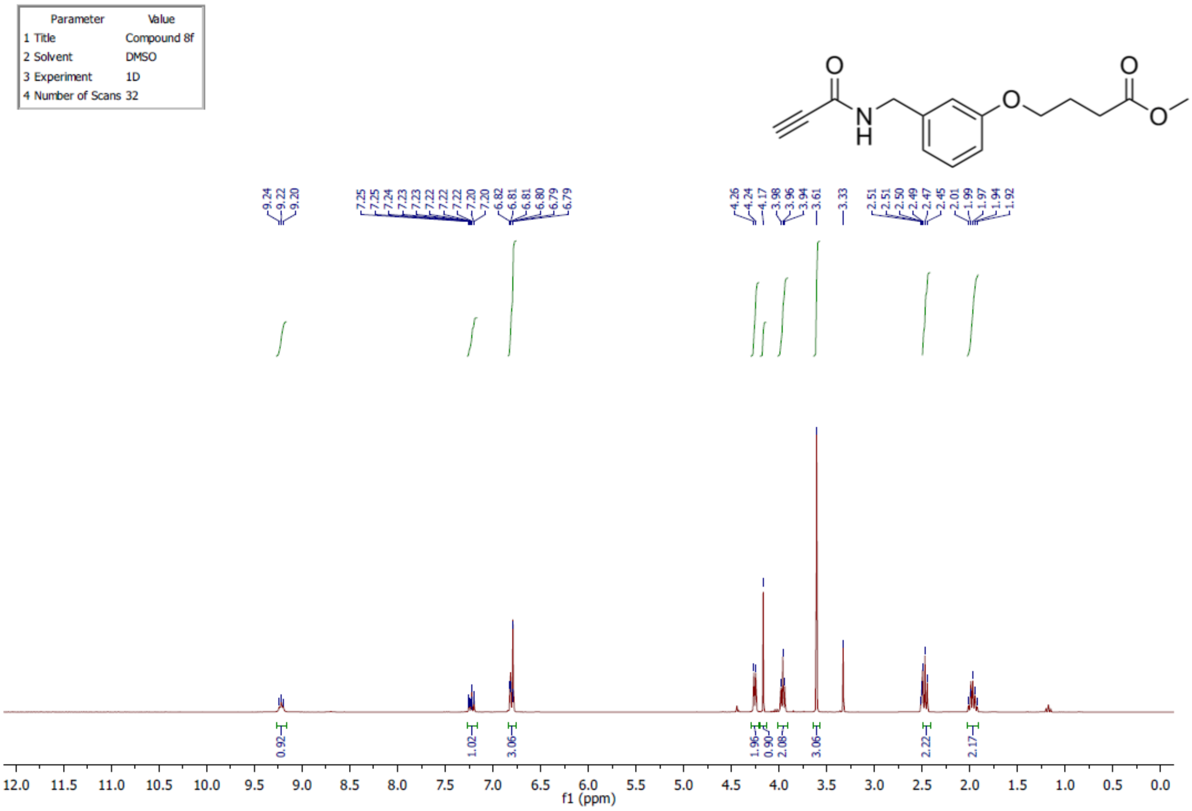

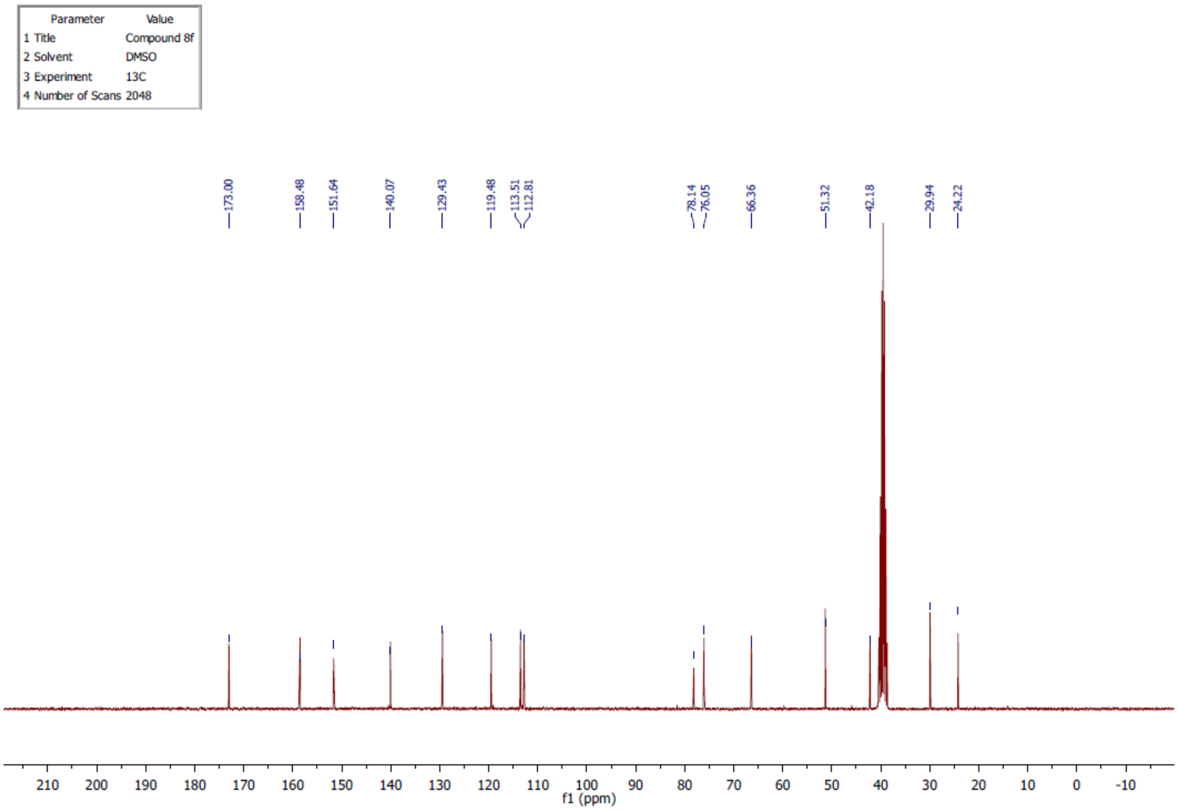
Parameter Value
1 Title Compound 8f
2 Solvent DMSO
3 Experiment 13C
4 Number of Scans 2048
173.00
158.48
151.64
140.07
129.43
119.48
113.51
112.81
78.14
76.05
66.36
51.32
42.18
29.94
24.22
210 200 190 180 170 160 150 140 130 120 110 100 90 80 70 60 50 40 30 20 10 0 -10
f1 (ppm)

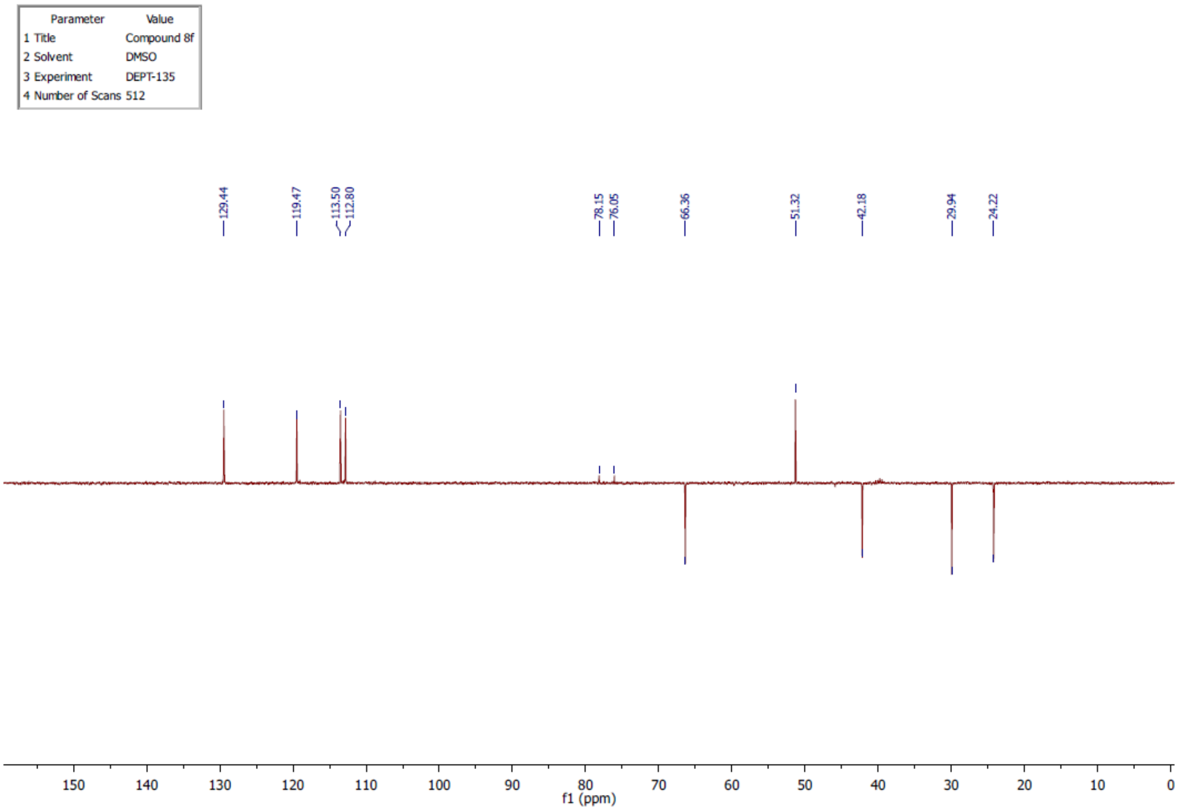
Parameter Value
1 Title Compound 8f
2 Solvent DMSO
3 Experiment DEPT-135
4 Number of Scans 512
129.44
119.47
113.50
112.80
78.15
76.05
66.36
51.32
42.18
29.94
24.22
150 140 130 120 110 100 90 80 70 60 50 40 30 20 10 0
f1 (ppm)

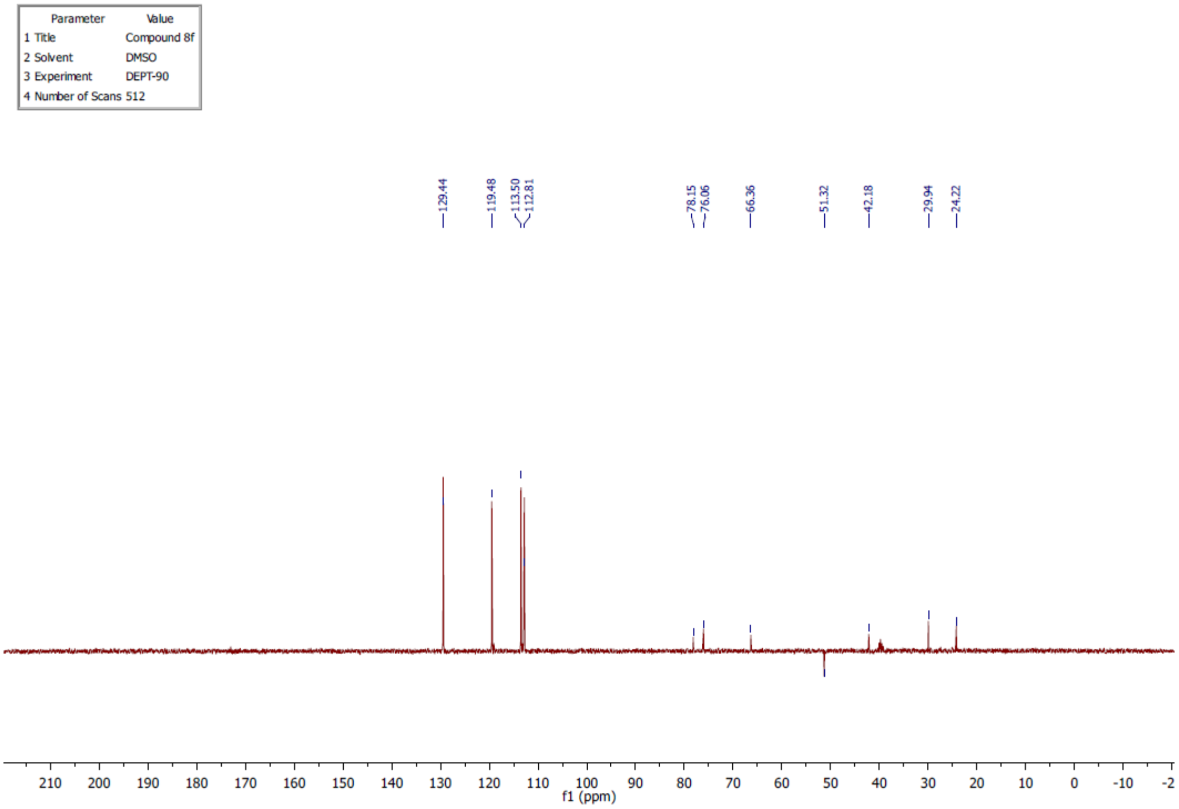

4-[3-(Prop-2-ynamidomethyl)phenoxy]butanoic acid (8g)

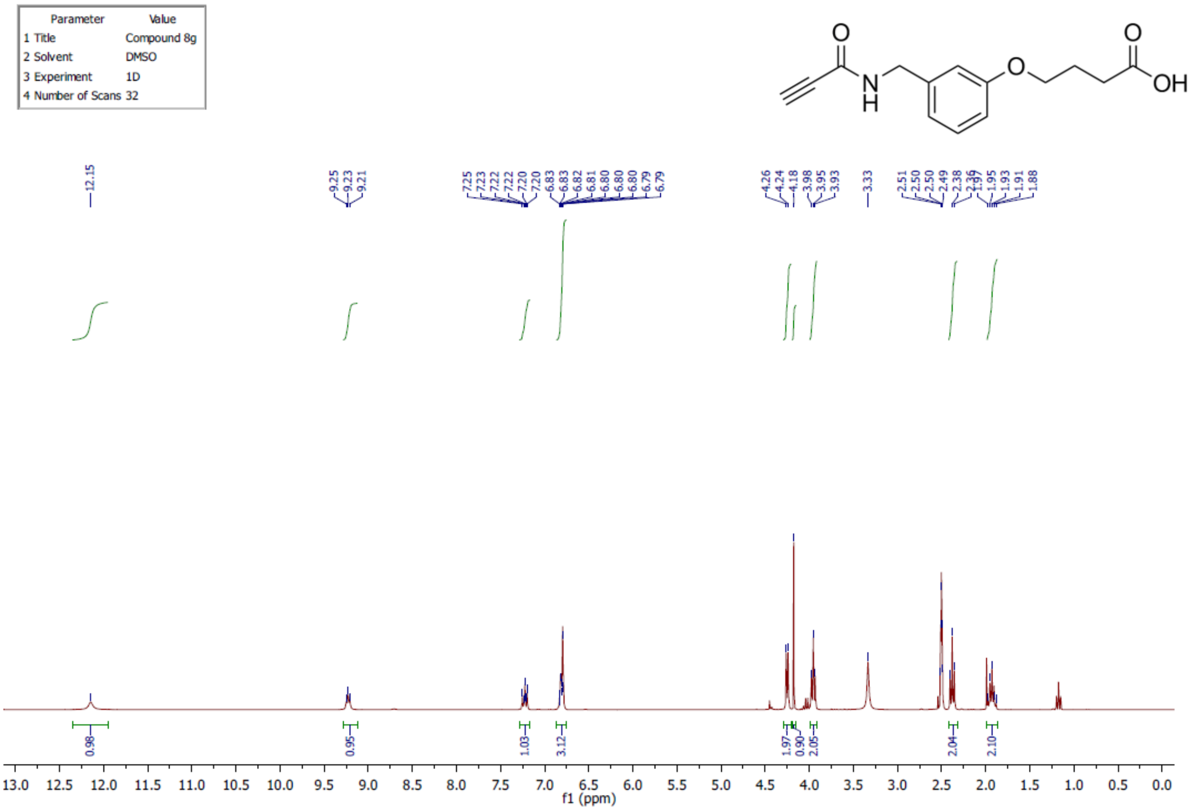

Parameter	Value
1 Title	Compound 8g
2 Solvent	DMSO
3 Experiment	13C
4 Number of Scans	2048

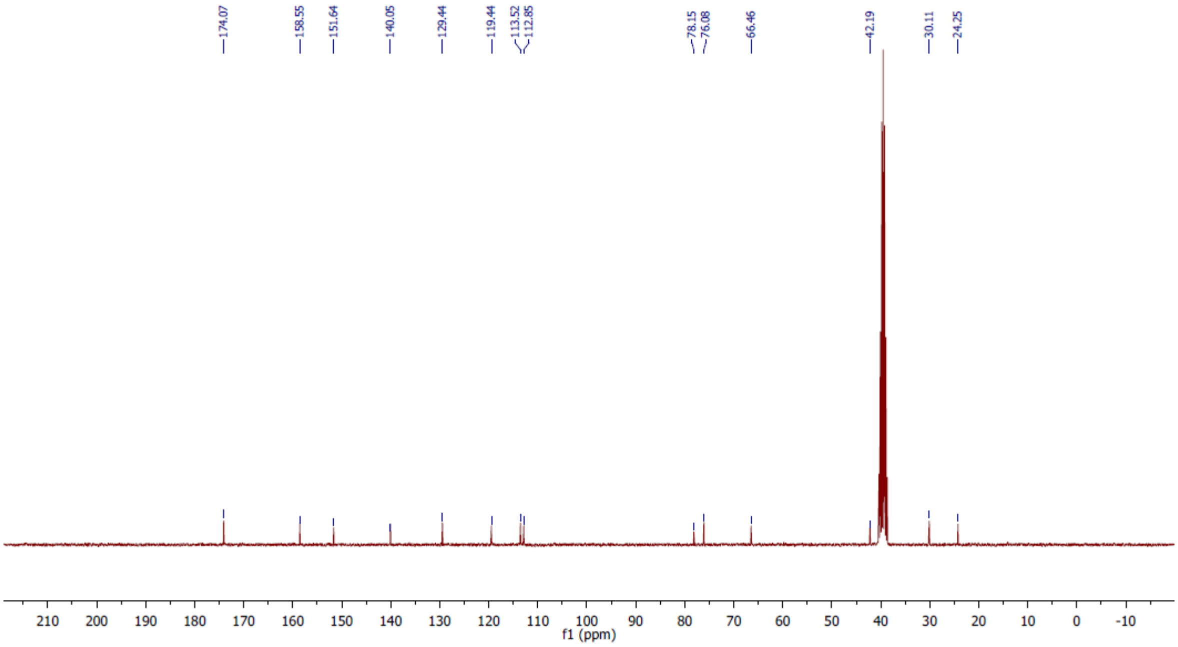

Parameter	Value
1 Title	Compound 8g
2 Solvent	DMSO
3 Experiment	DEPT-135
4 Number of Scans	512

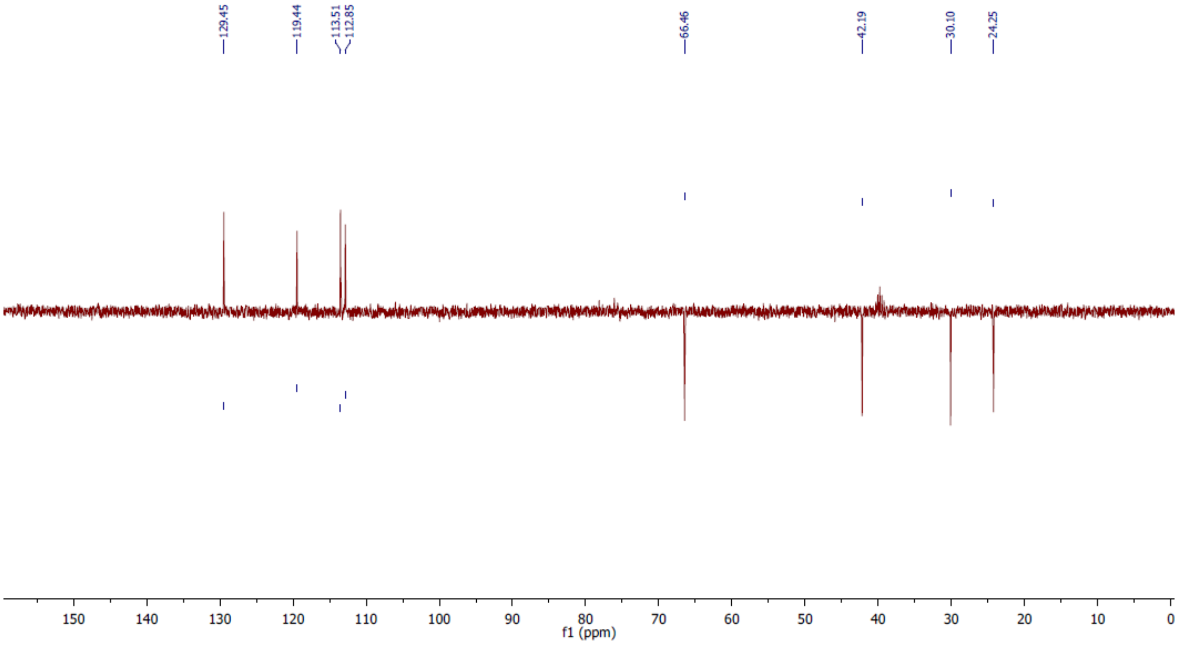

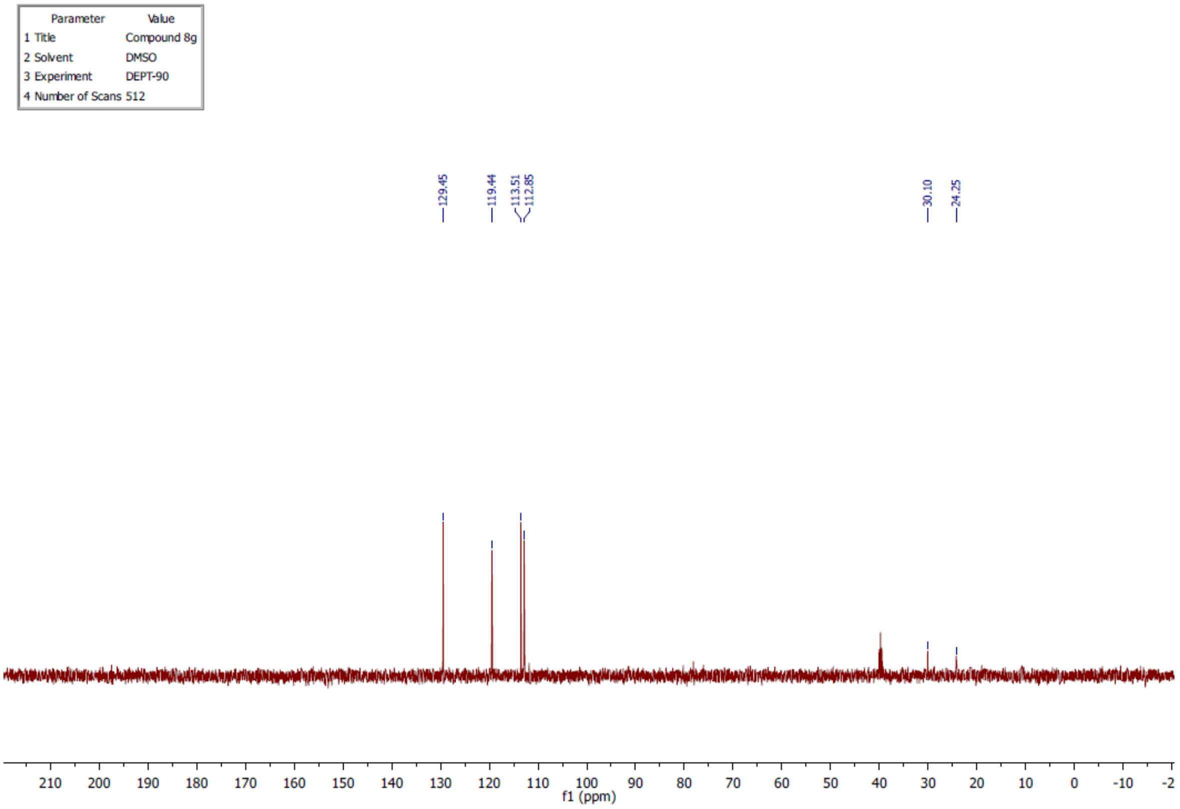

N-Benzyl-3-(1-benzyl-2,4-dioxo-1,2,3,4-tetrahydropyrimidin-5-yl)prop-2-ynamide (2)

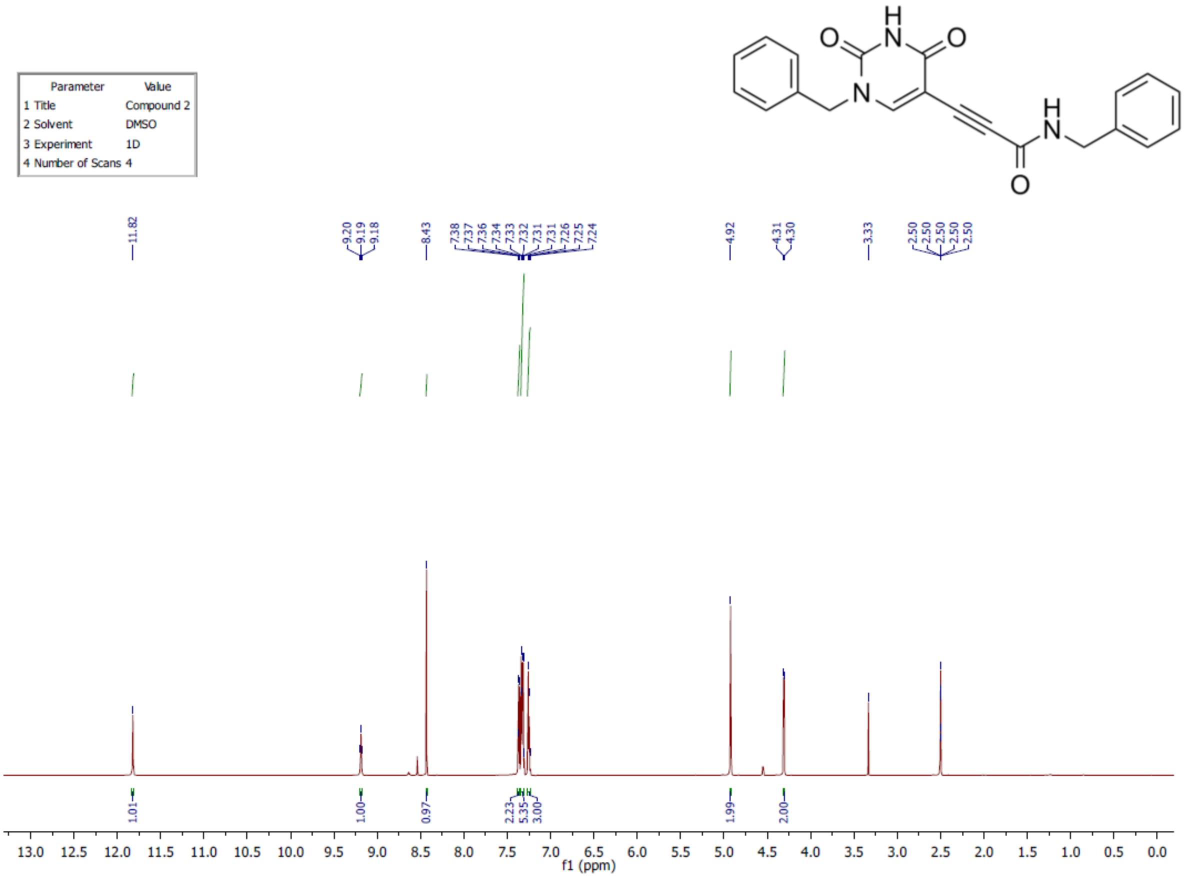

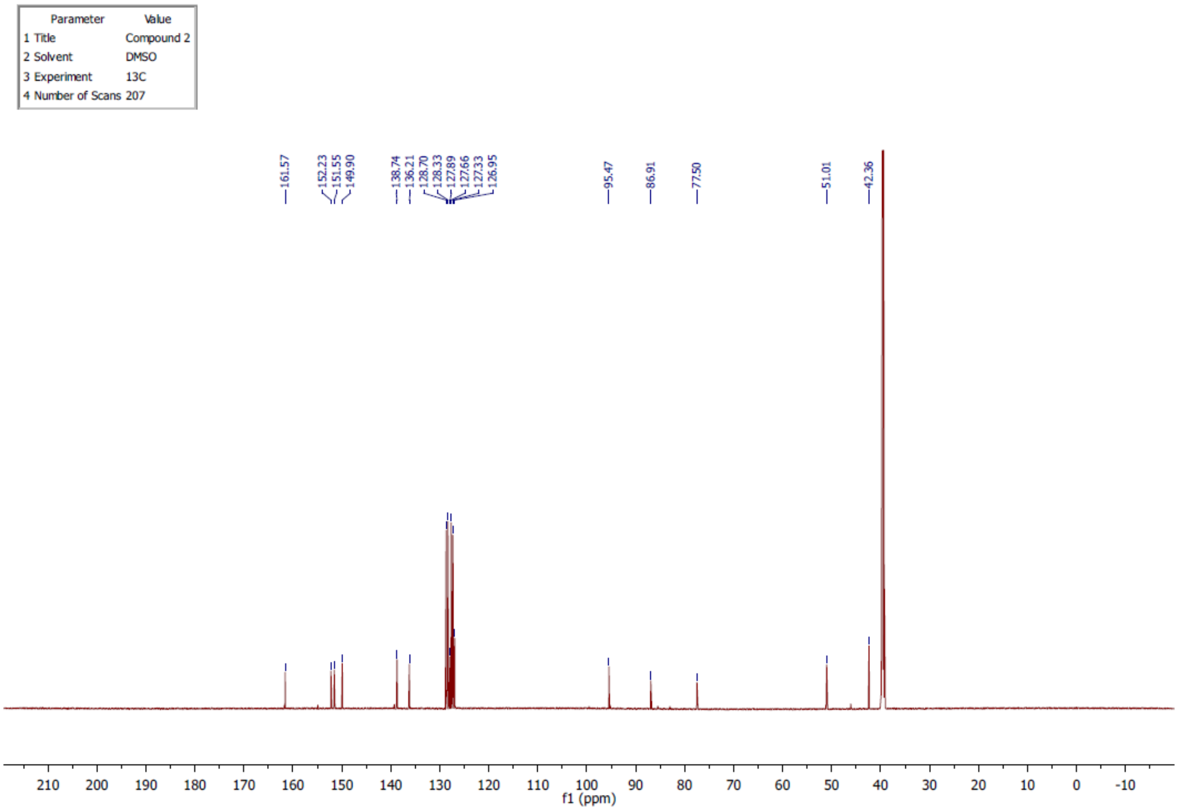

Parameter Value
1 Title Compound 2
2 Solvent DMSO
3 Experiment 13C
4 Number of Scans 207
161.57
152.23
151.55
149.90
138.74
136.21
128.70
128.33
127.89
127.66
127.33
126.95
95.47
86.91
77.50
51.01
42.36
f1 (ppm)

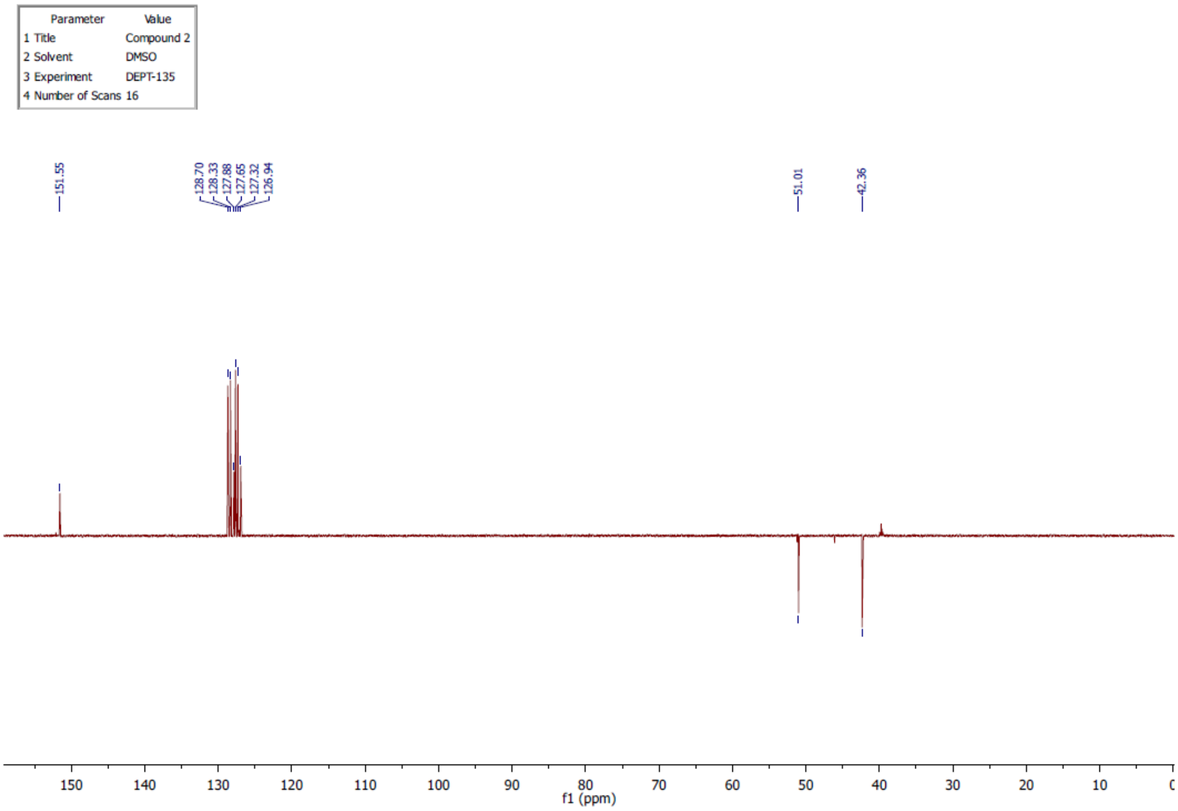

Parameter Value
1 Title Compound 2
2 Solvent DMSO
3 Experiment DEPT-135
4 Number of Scans 16
151.55
128.70
128.33
127.88
127.65
127.32
126.94
51.01
42.36
f1 (ppm)

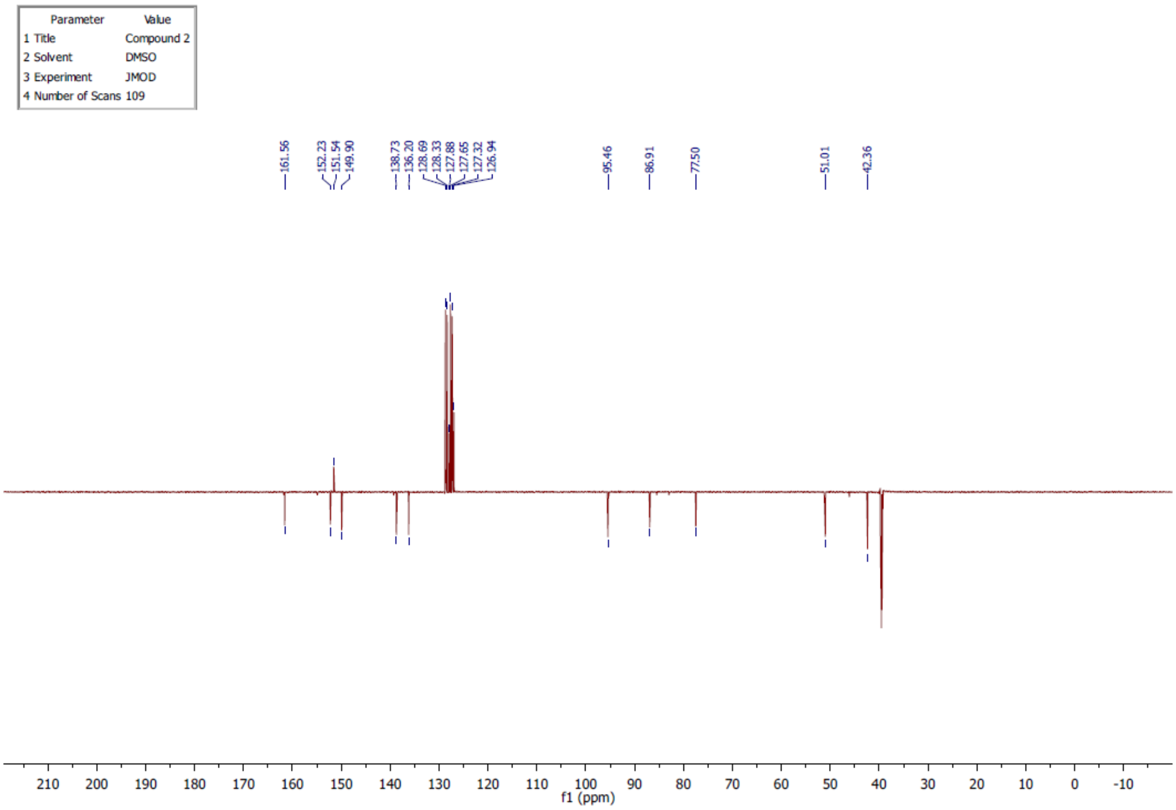

4-{[5-(2-{[(3-methoxyphenyl)methyl]carbamoyl}eth-1-yn-1-yl)-2,4-dioxo-1,2,3,4-tetrahydropyrimidin-1-yl]methyl}benzoic acid (3)

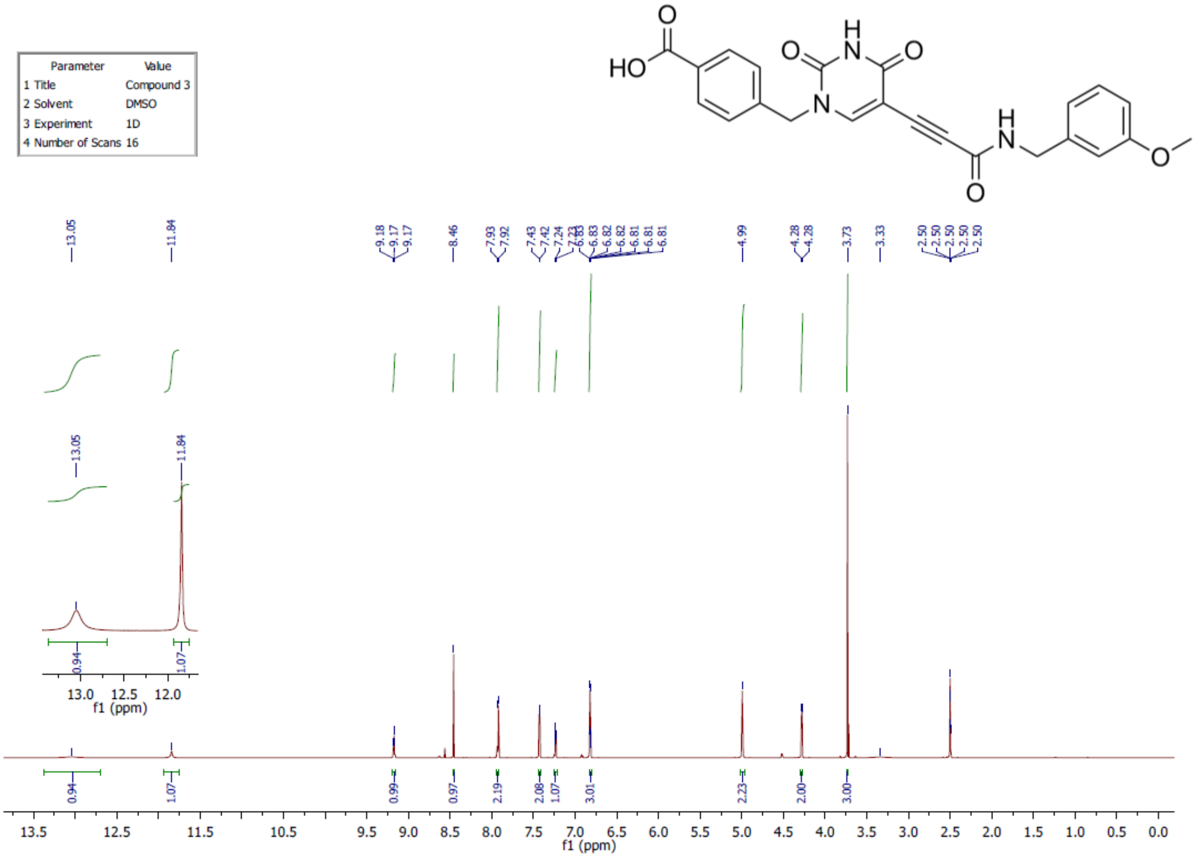

	Parameter	Value
1	Title	Compound 3
2	Solvent	DMSO
3	Experiment	13C
4	Number of Scans	1024

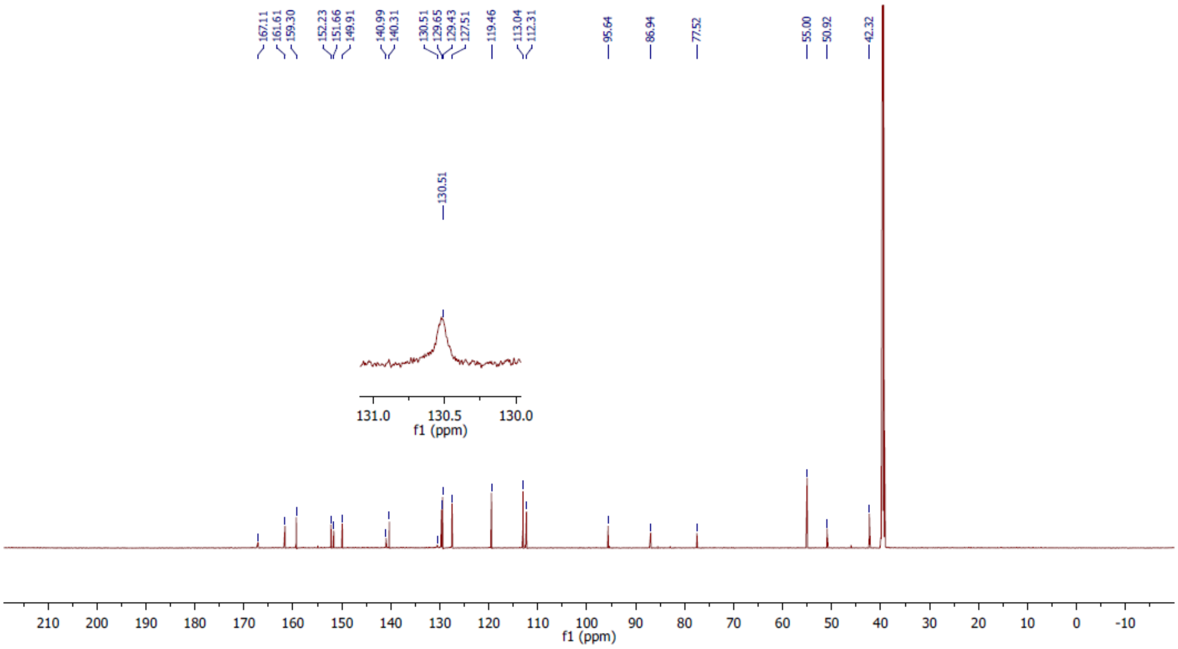

	Parameter	Value
1	Title	Compound 3
2	Solvent	DMSO
3	Experiment	DEPT-135
4	Number of Scans	16

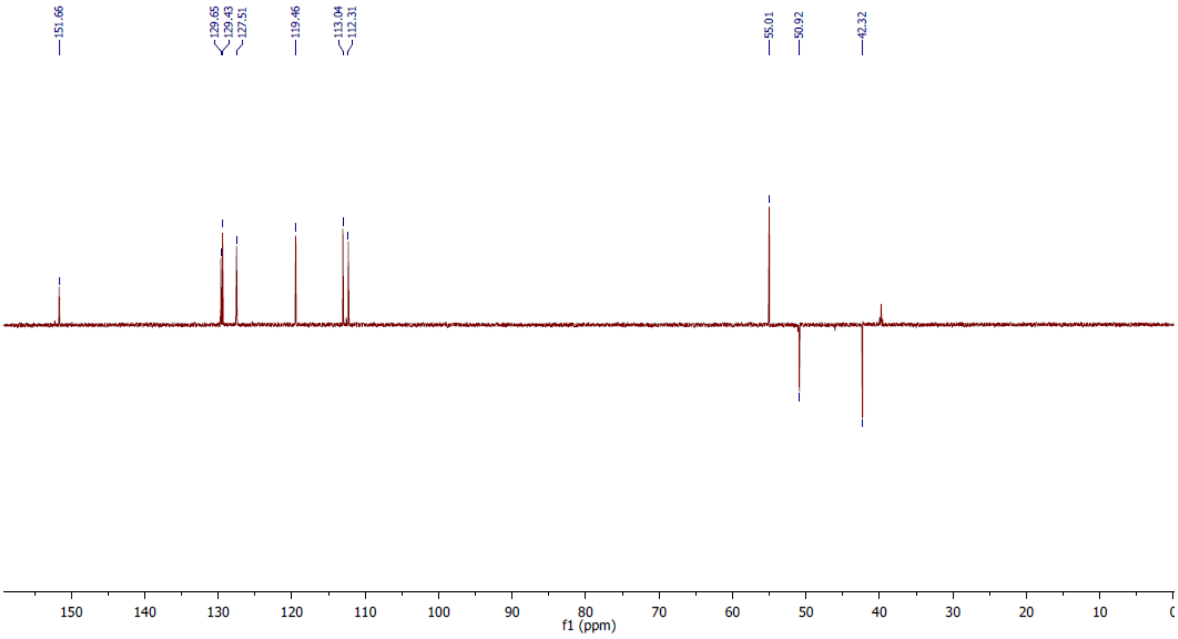

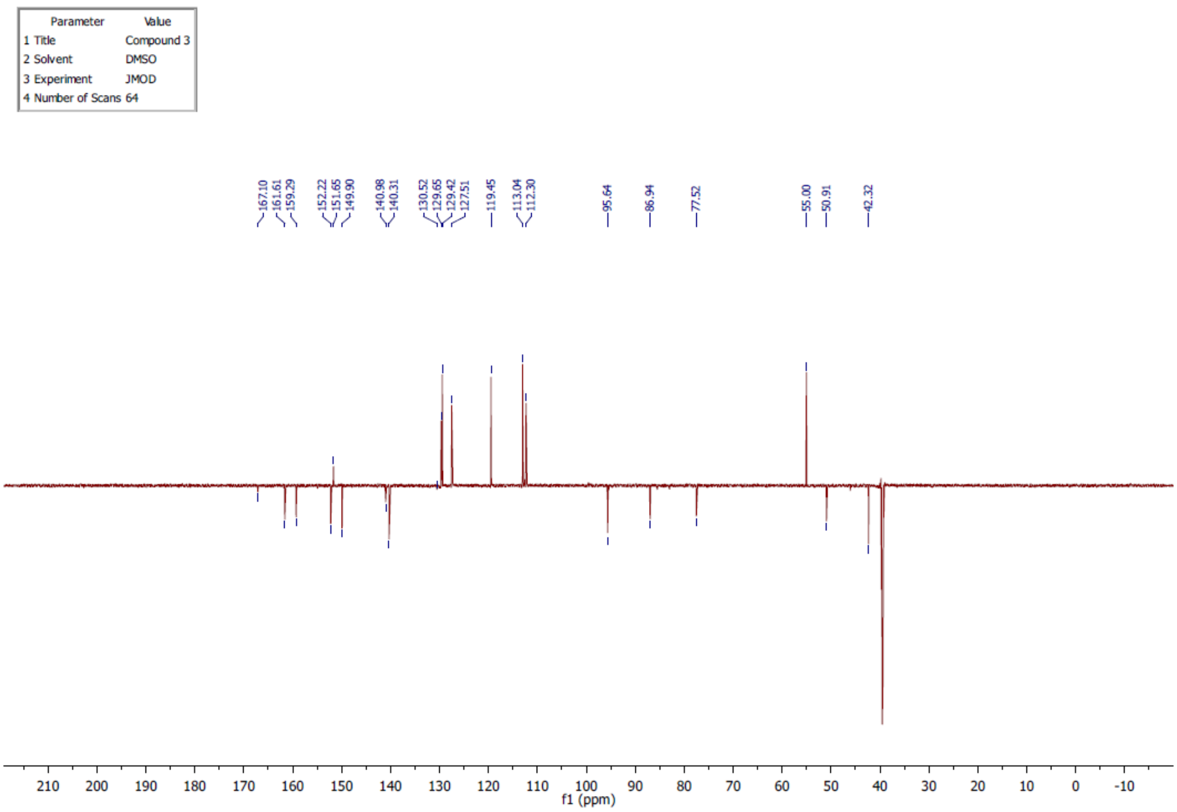

4-({5-[2-({[3-(3-Carboxypropoxy)phenyl]methyl}carbamoyl)eth-1-yn-1-yl]-2,4-dioxo-1,2,3,4-tetrahydropyrimidin-1-yl}methyl)benzoic acid (4)

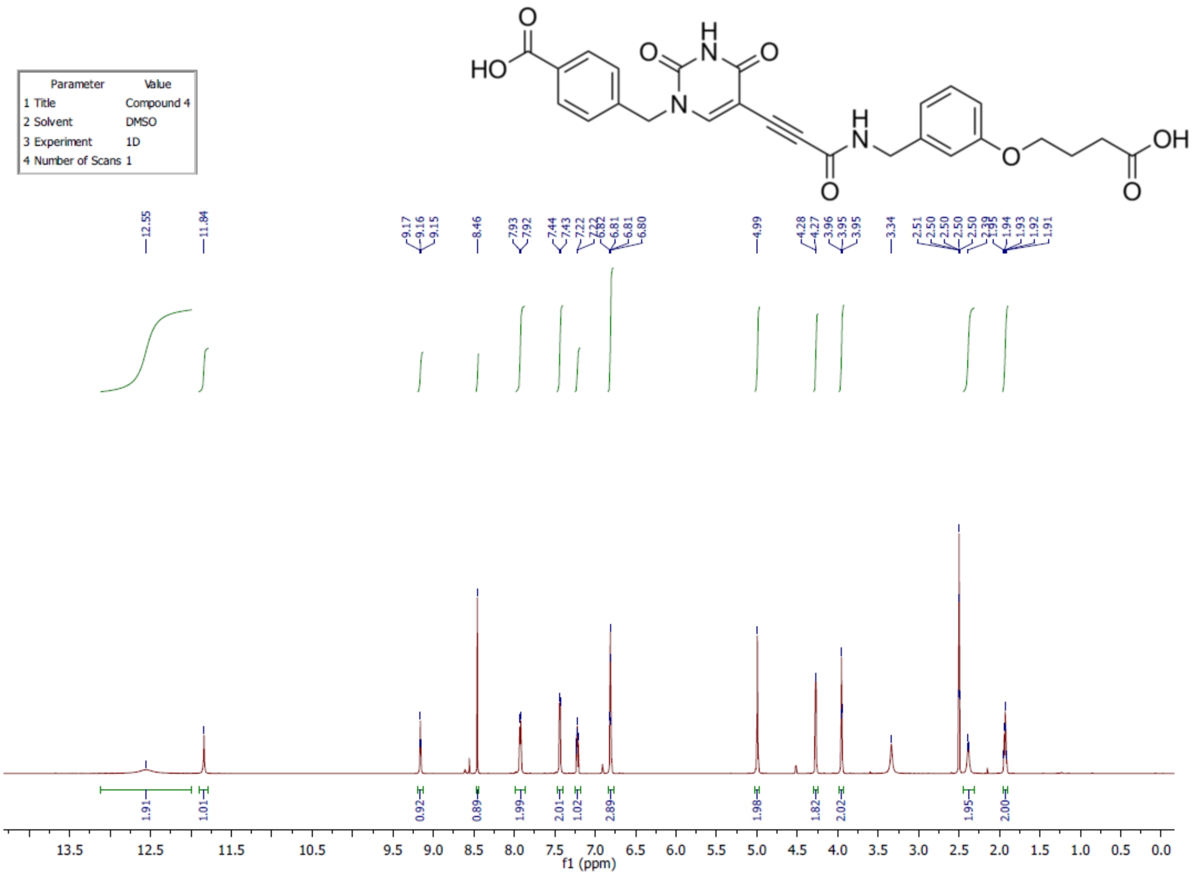

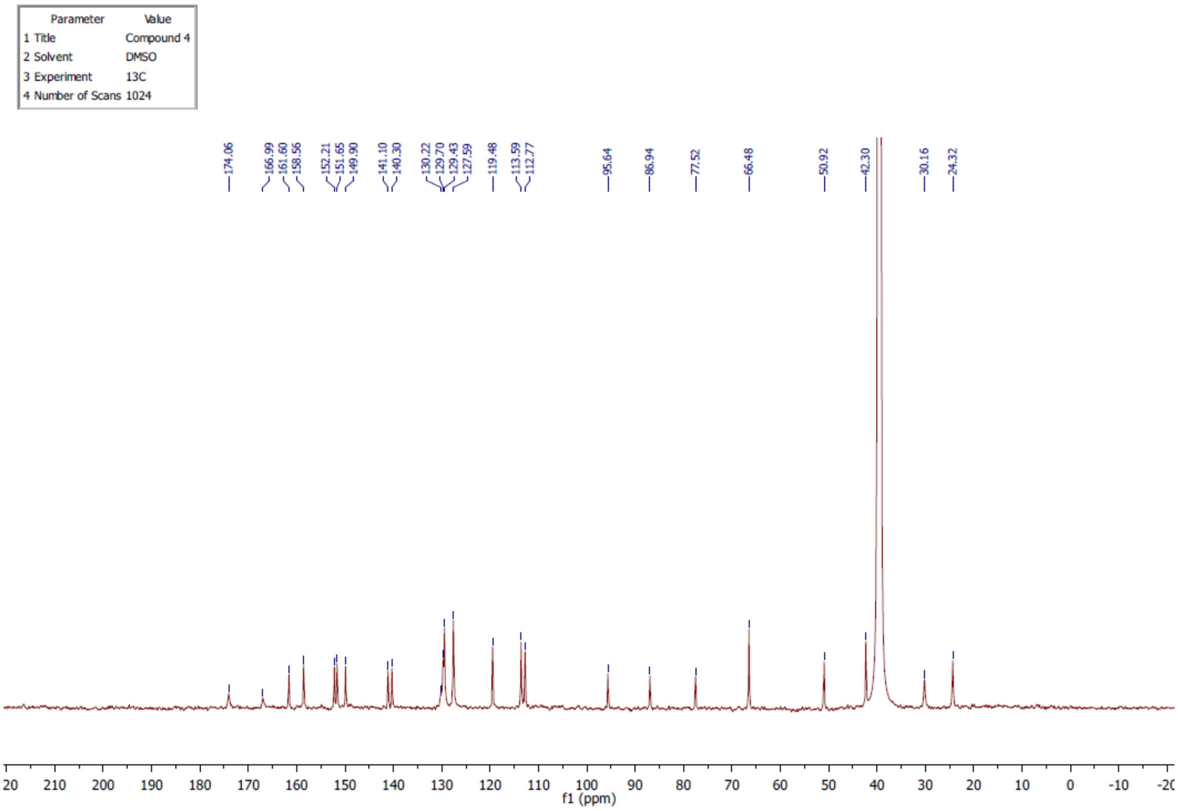
Parameter Value
1 Title Compound 4
2 Solvent DMSO
3 Experiment 13C
4 Number of Scans 1024
174.06
166.99
161.60
158.56
152.21
151.65
149.90
141.10
140.30
130.22
129.70
129.43
127.59
119.48
113.59
112.77
95.64
86.94
77.52
66.48
50.92
42.30
30.16
24.32
f1 (ppm)

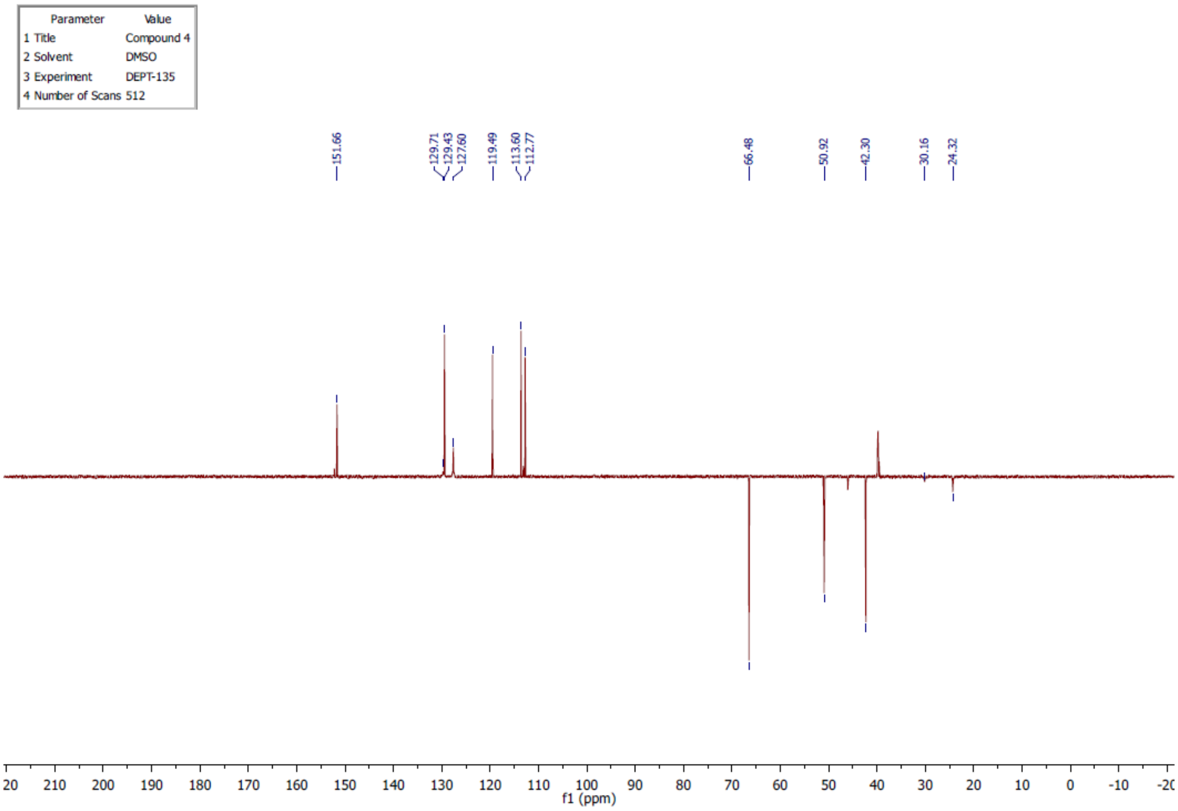
Parameter Value
1 Title Compound 4
2 Solvent DMSO
3 Experiment DEPT-135
4 Number of Scans 512
151.66
129.71
129.43
127.60
119.49
113.60
112.77
66.48
50.92
42.30
30.16
24.32
f1 (ppm)

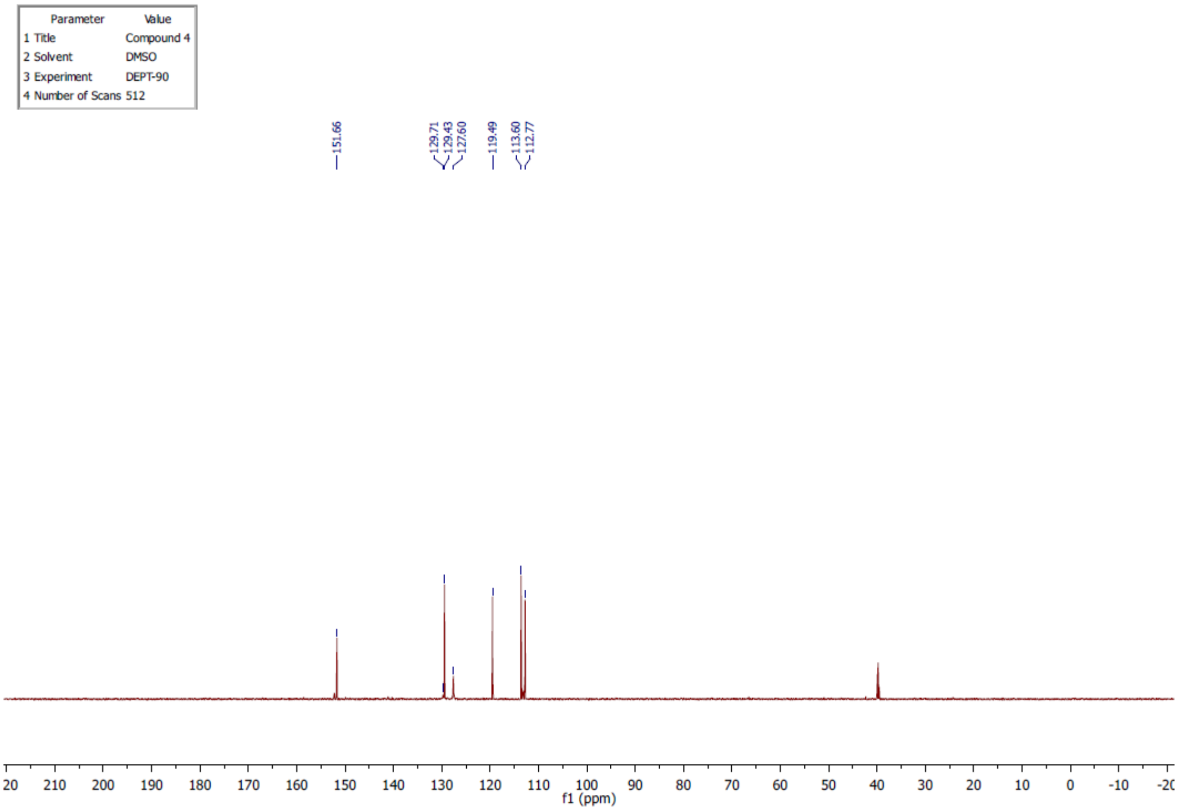

Parameter Value
1 Title Compound 4
2 Solvent DMSO
3 Experiment DEPT-90
4 Number of Scans 512
151.66
129.71
129.43
127.60
119.49
113.60
112.77
f1 (ppm)

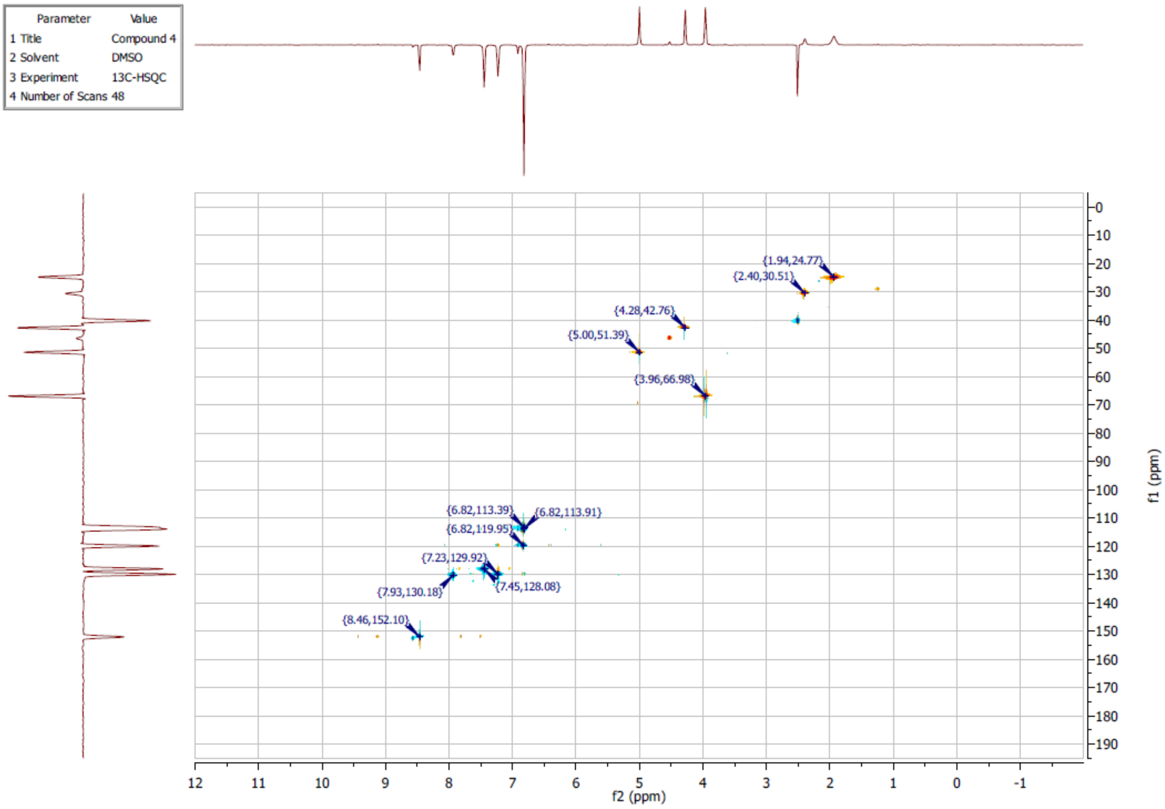

Parameter Value
1 Title Compound 4
2 Solvent DMSO
3 Experiment 13C-HSQC
4 Number of Scans 48
{1.94,24.77}
{2.40,30.51}
{4.28,42.76}
{5.00,51.39}
{3.96,66.98}
{6.82,113.39}
{6.82,113.91}
{6.82,119.95}
{7.23,129.92}
{7.93,130.18}
{7.45,128.08}
{8.46,152.10}
f2 (ppm)
f1 (ppm)

3-(1-Benzyl-2,4-dioxo-1,2,3,4-tetrahydropyrimidin-5-yl)-N-[(2-fluorophenyl)methyl]prop-2-ynamide (9a)

Parameter	Value
1 Title	Compound 9a
2 Solvent	DMSO
3 Experiment	1D
4 Number of Scans	32

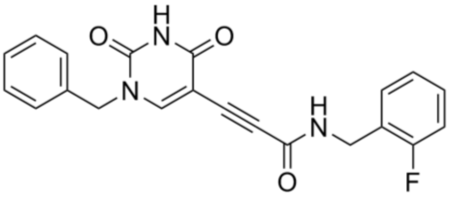

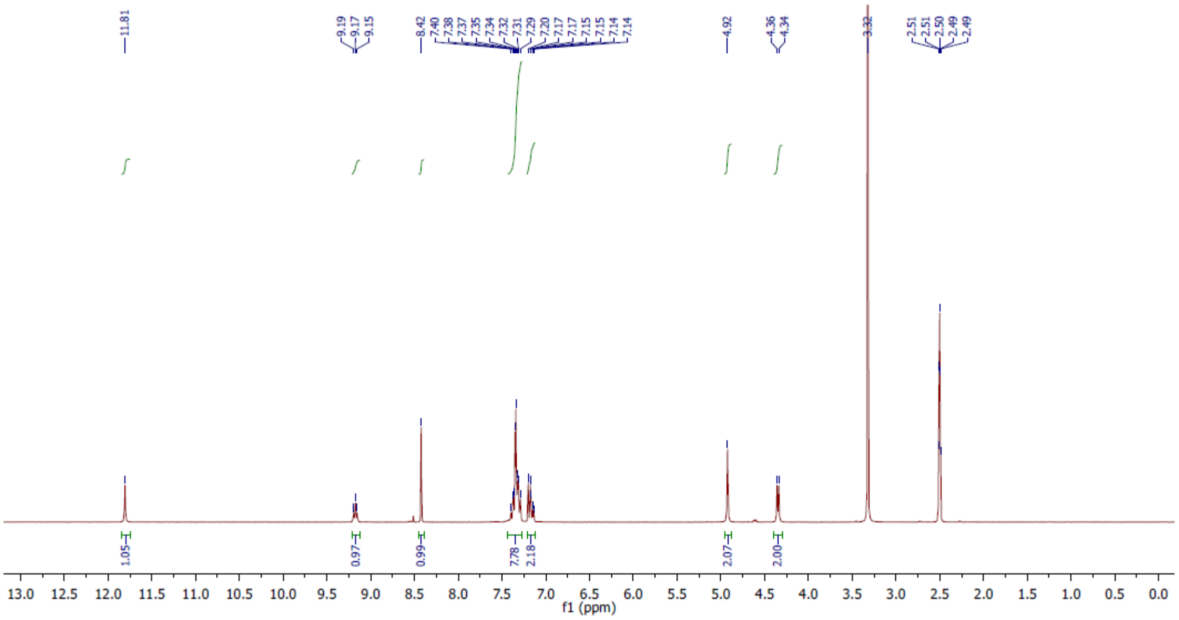

Parameter	Value
1 Title	Compound 9a
2 Solvent	DMSO
3 Experiment	13C
4 Number of Scans	1024

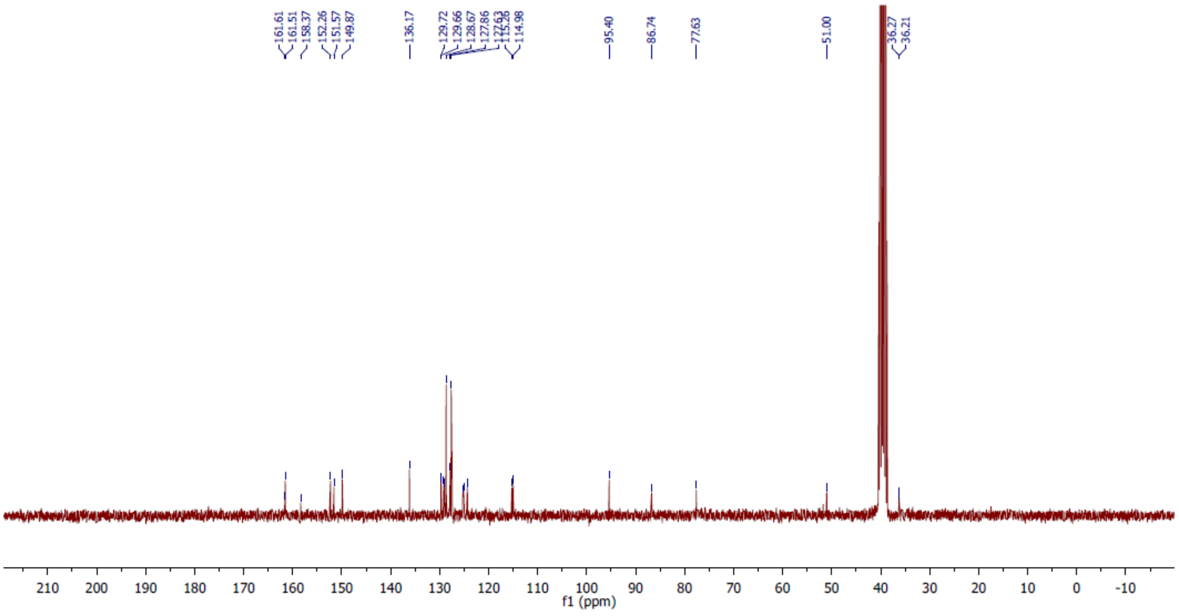

Parameter	Value
1 Title	Compound 9a
2 Solvent	DMSO
3 Experiment	DEPT-135
4 Number of Scans	256

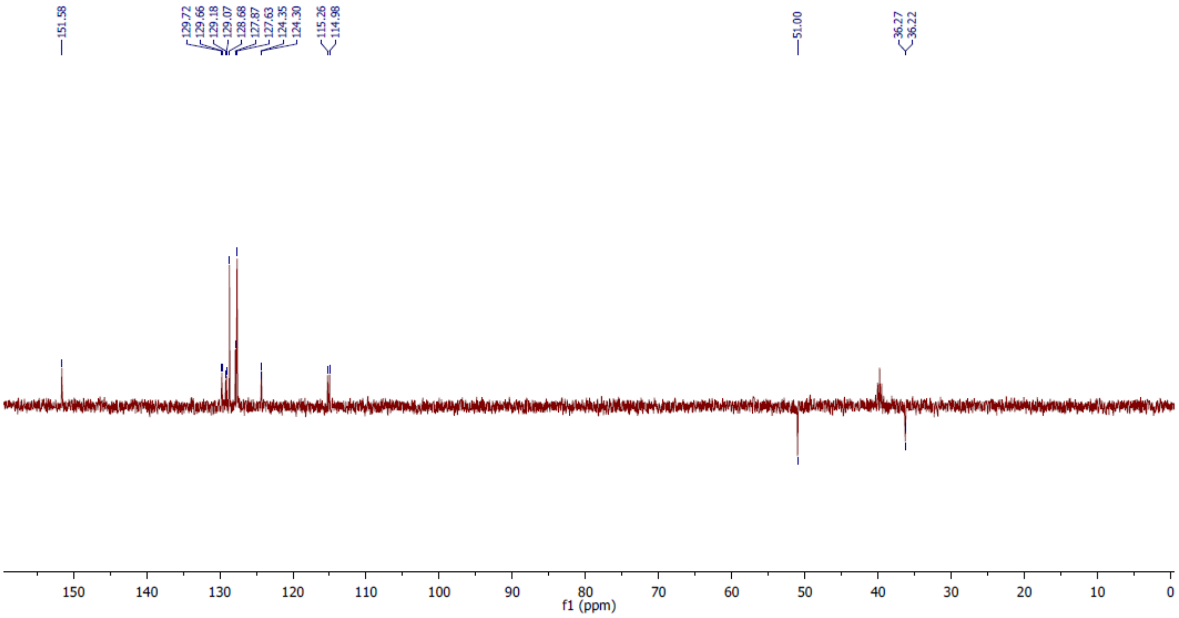

Parameter	Value
1 Title	Compound 9a
2 Solvent	DMSO
3 Experiment	DEPT-90
4 Number of Scans	256

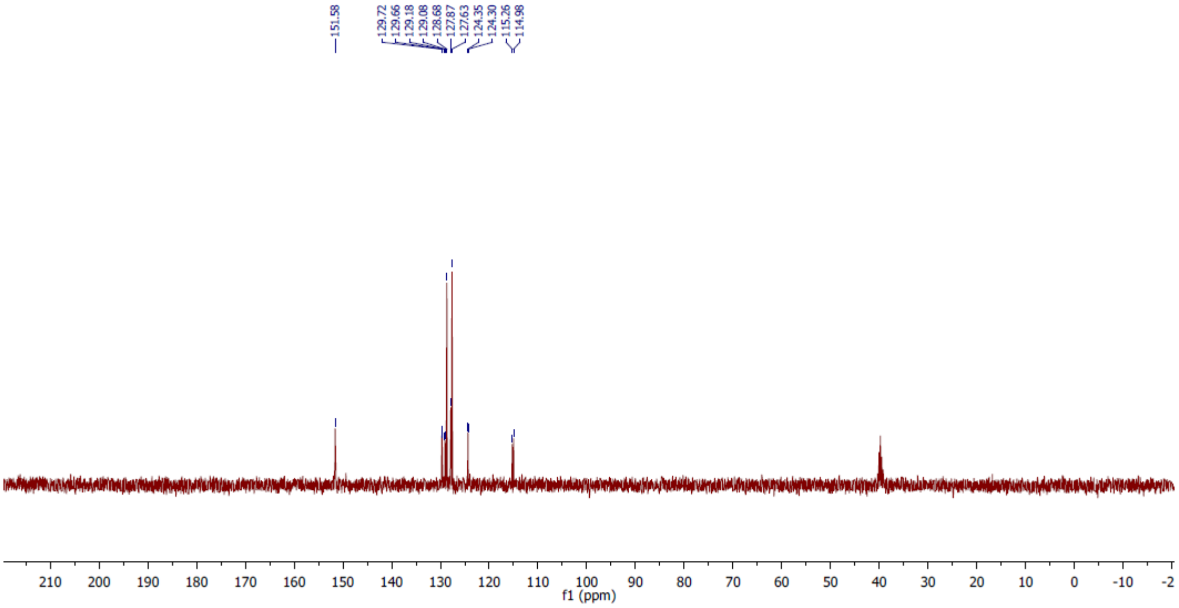

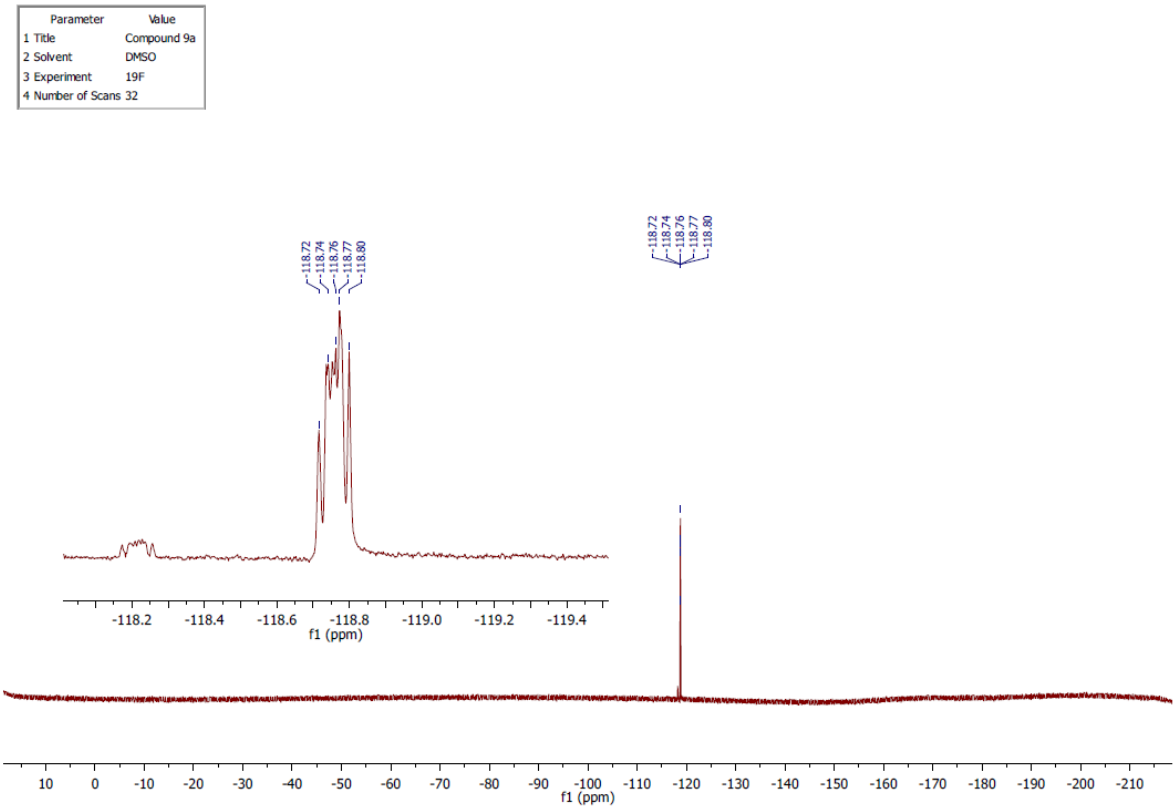

3-(1-Benzyl-2,4-dioxo-1,2,3,4-tetrahydropyrimidin-5-yl)-N-[(3-fluorophenyl)methyl]prop-2-ynamide (9b)

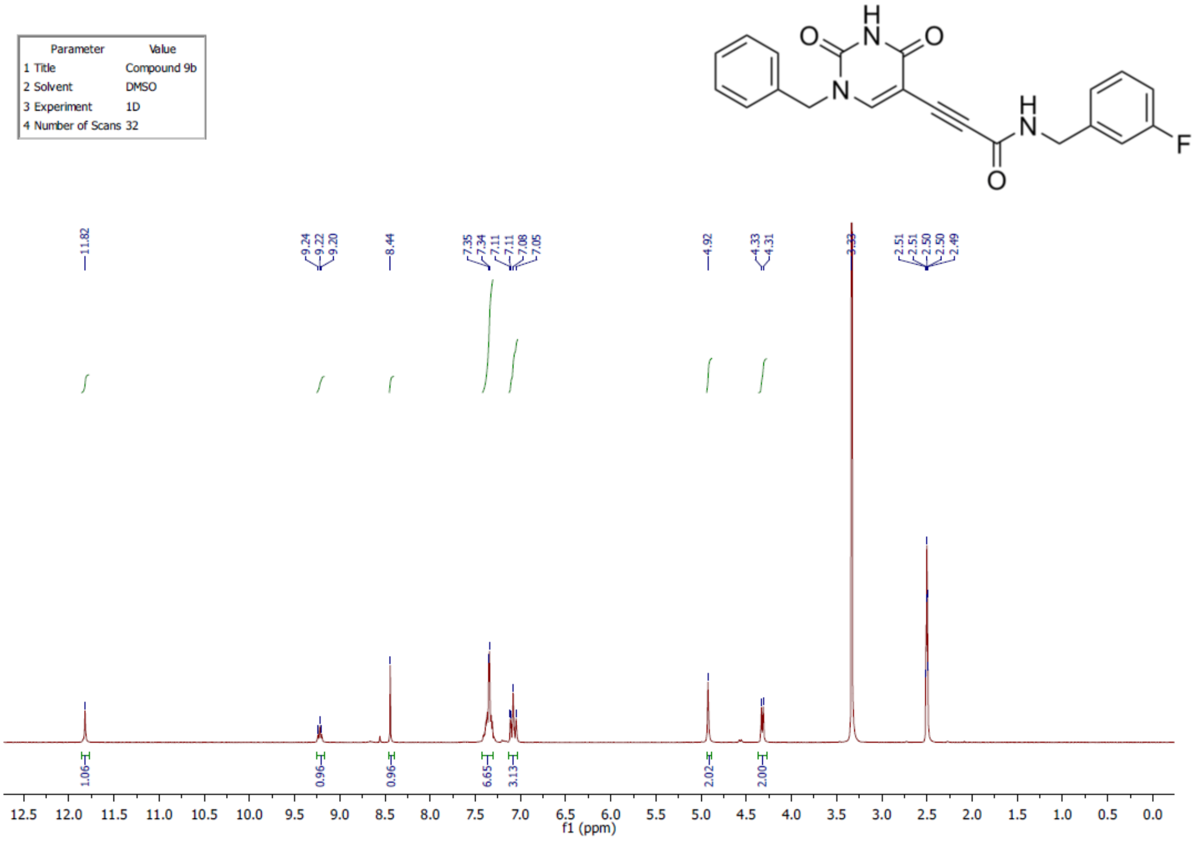

Parameter	Value
1 Title	Compound 9b
2 Solvent	DMSO
3 Experiment	13C
4 Number of Scans	1024

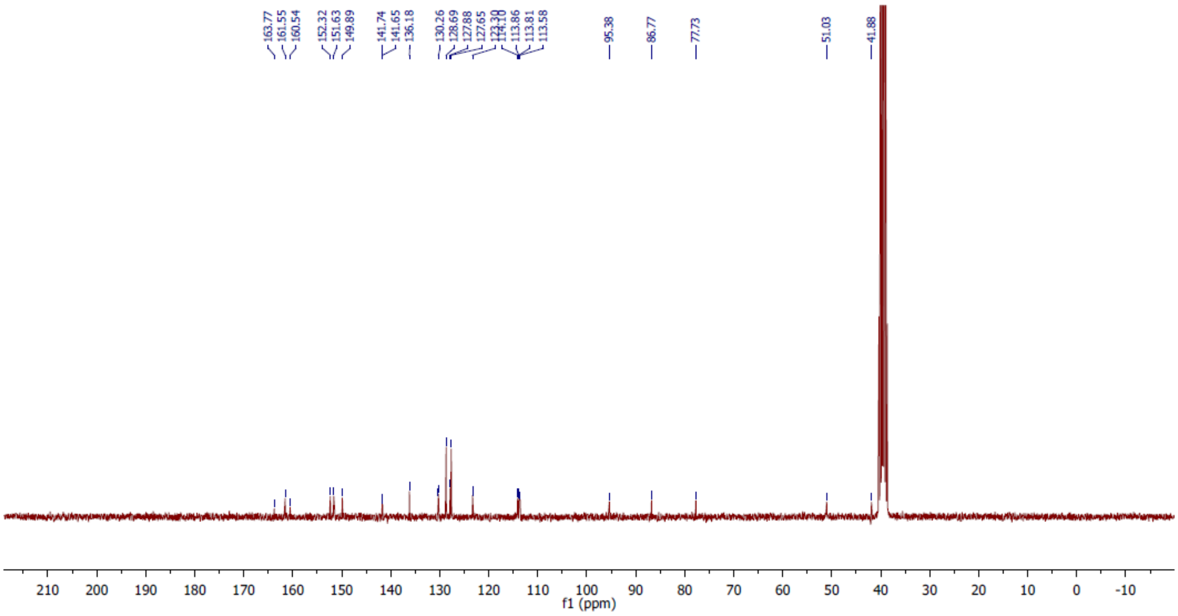

Parameter	Value
1 Title	Compound 9b
2 Solvent	DMSO
3 Experiment	DEPT-135
4 Number of Scans	256

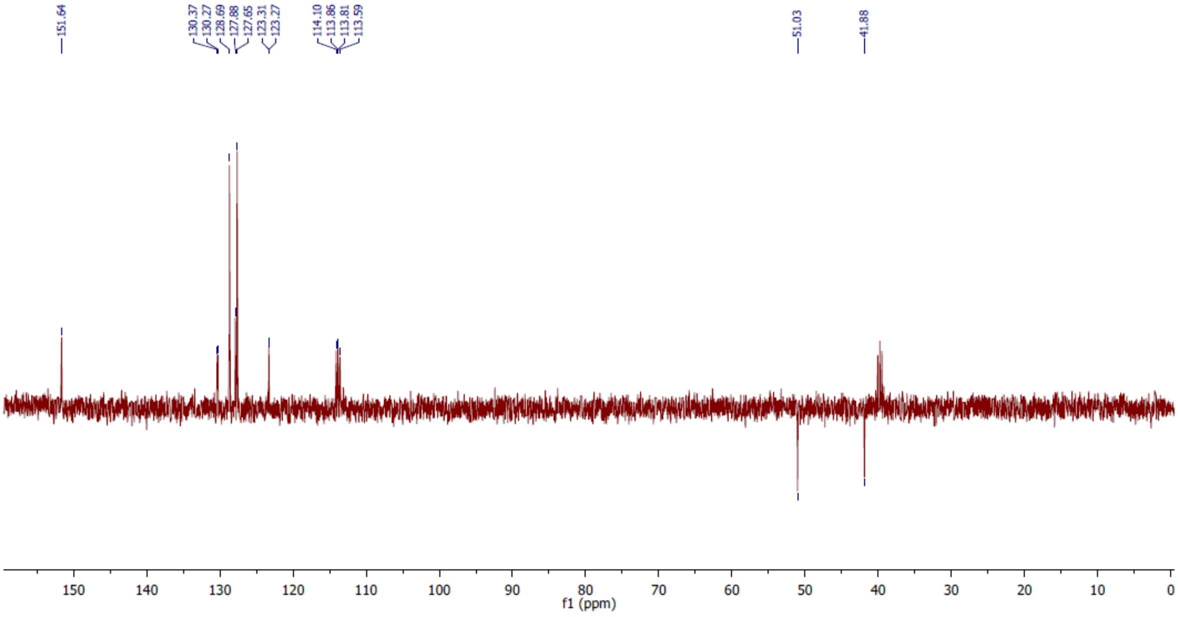

Parameter	Value
1 Title	Compound 9b
2 Solvent	DMSO
3 Experiment	DEPT-90
4 Number of Scans	256

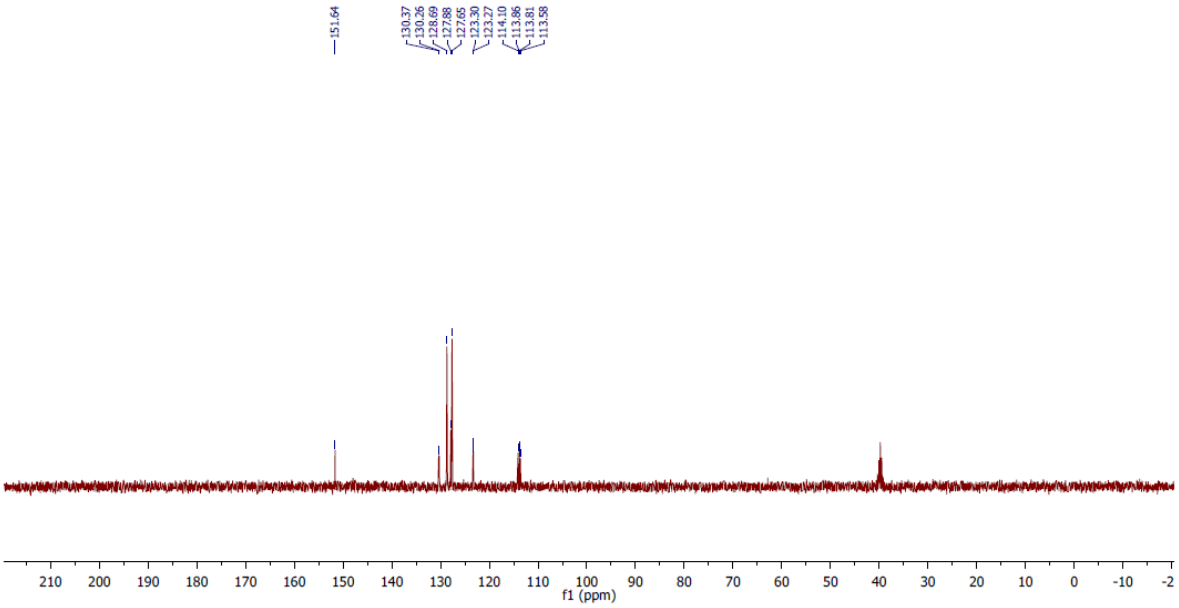

Parameter	Value
1 Title	Compound 9b
2 Solvent	DMSO
3 Experiment	19F
4 Number of Scans	32

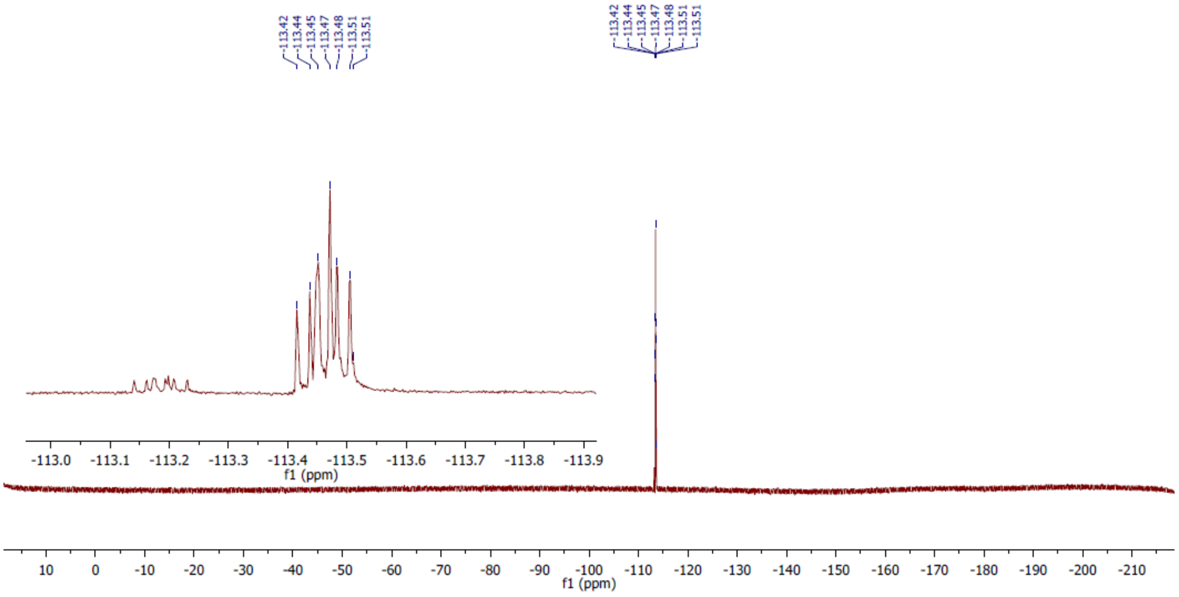

3-(1-Benzyl-2,4-dioxo-1,2,3,4-tetrahydropyrimidin-5-yl)-N-[(4-fluorophenyl)methyl]prop-2-ynamide (9c)

Parameter	Value
1 Title	Compound 9c
2 Solvent	DMSO
3 Experiment	1D
4 Number of Scans	32

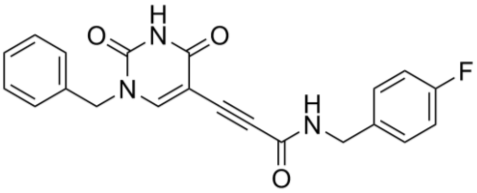

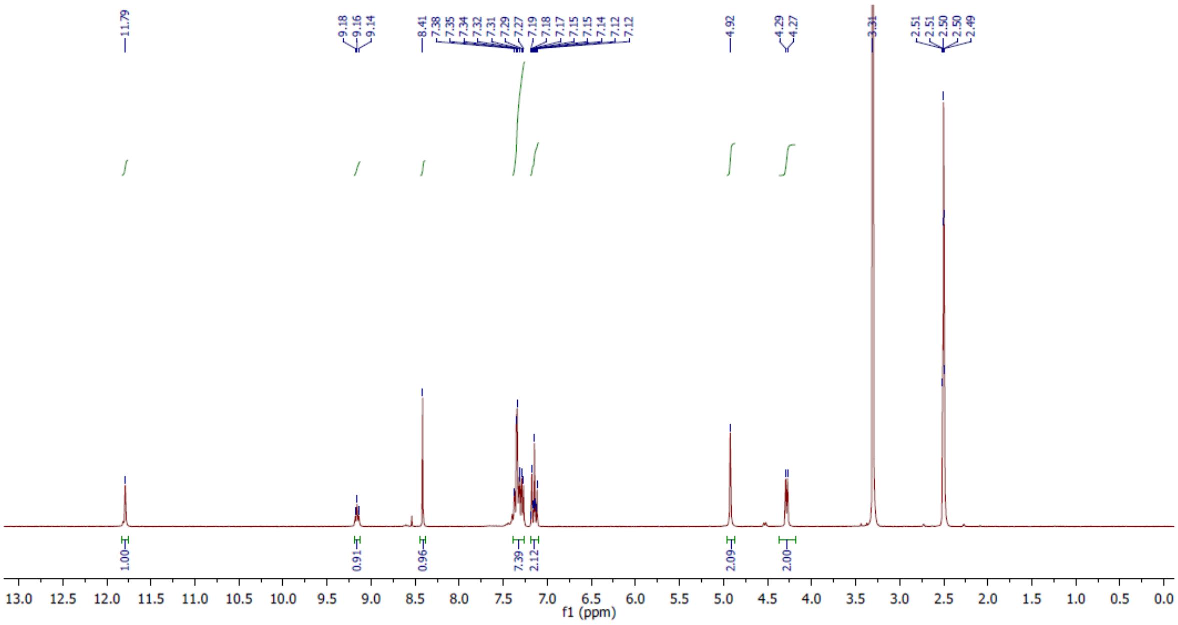

Parameter	Value
1 Title	Compound 9c
2 Solvent	DMSO
3 Experiment	13C
4 Number of Scans	1024

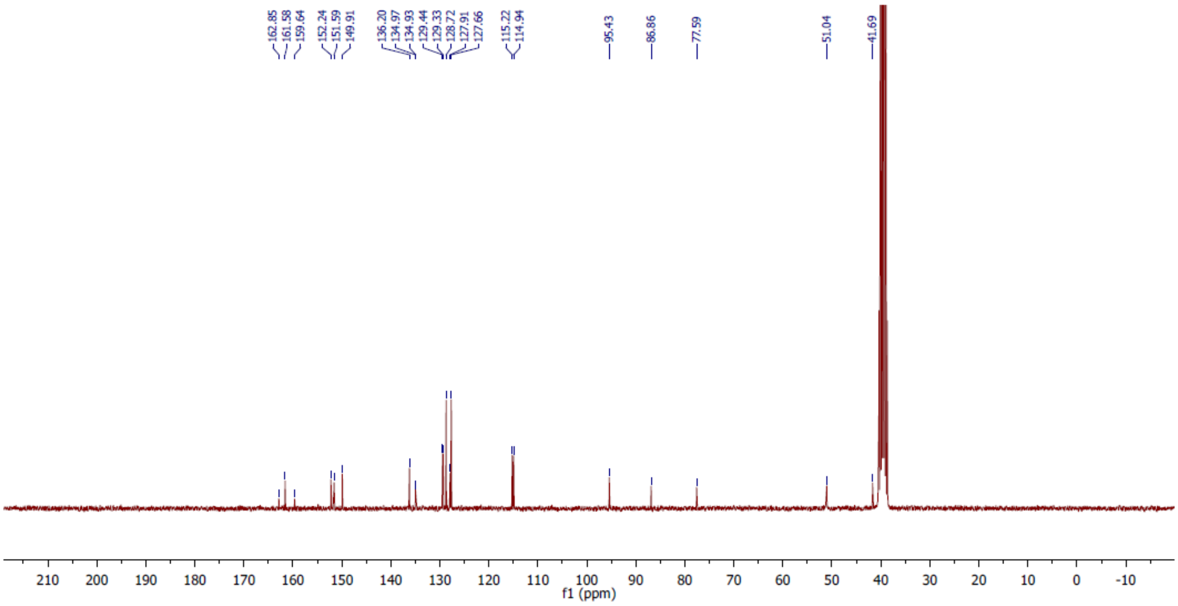

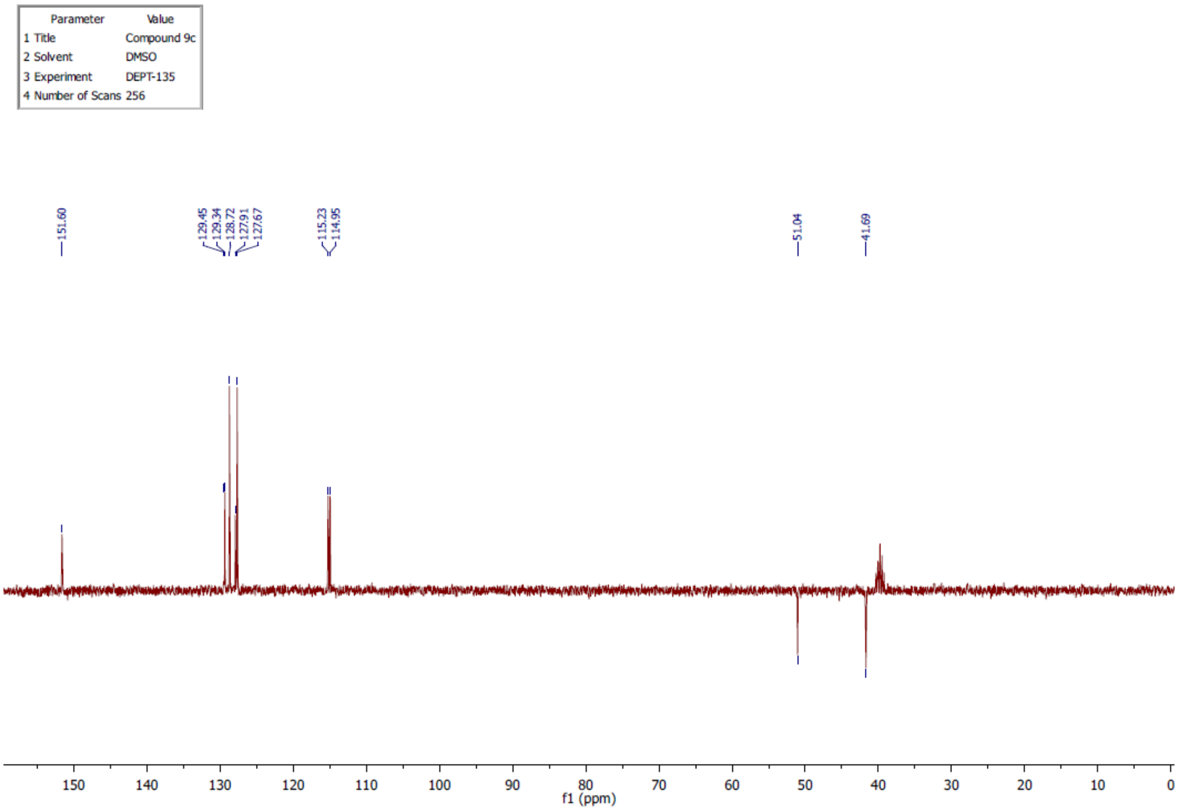
Parameter Value
1 Title Compound 9c
2 Solvent DMSO
3 Experiment DEPT-135
4 Number of Scans 256
151.60
129.45
129.34
128.72
127.91
127.67
115.23
114.95
51.04
41.69
f1 (ppm)

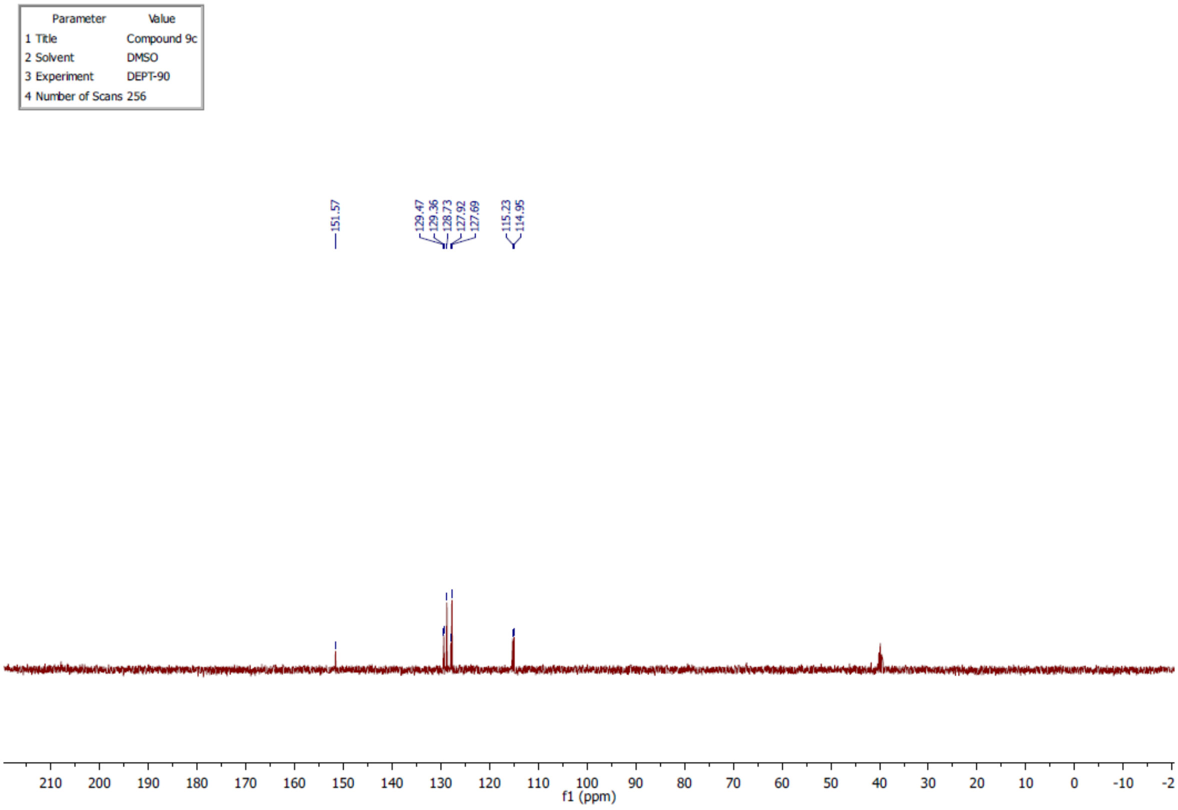
Parameter Value
1 Title Compound 9c
2 Solvent DMSO
3 Experiment DEPT-90
4 Number of Scans 256
151.57
129.47
129.36
128.73
127.92
127.69
115.23
114.95
f1 (ppm)

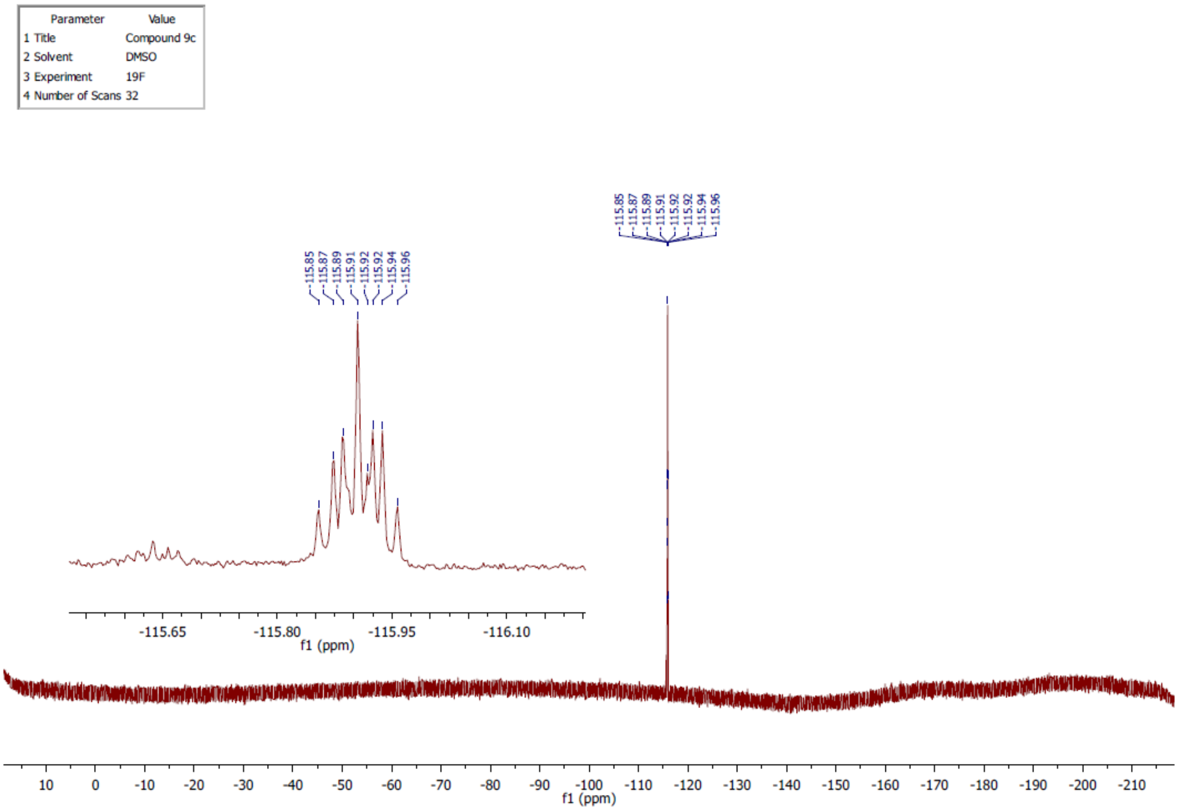

Parameter Value
1 Title Compound 9c
2 Solvent DMSO
3 Experiment 19F
4 Number of Scans 32
-115.85
-115.87
-115.89
-115.91
-115.92
-115.92
-115.94
-115.96
-115.65
-115.80
-115.95
-116.10
f1 (ppm)
10
0
-10
-20
-30
-40
-50
-60
-70
-80
-90
-100
-110
-120
-130
-140
-150
-160
-170
-180
-190
-200
-210
f1 (ppm)

ESI-TOF-HRMS

1-Benzyl-5-iodo-1,2,3,4-tetrahydropyrimidine-2,4-dione (5a)

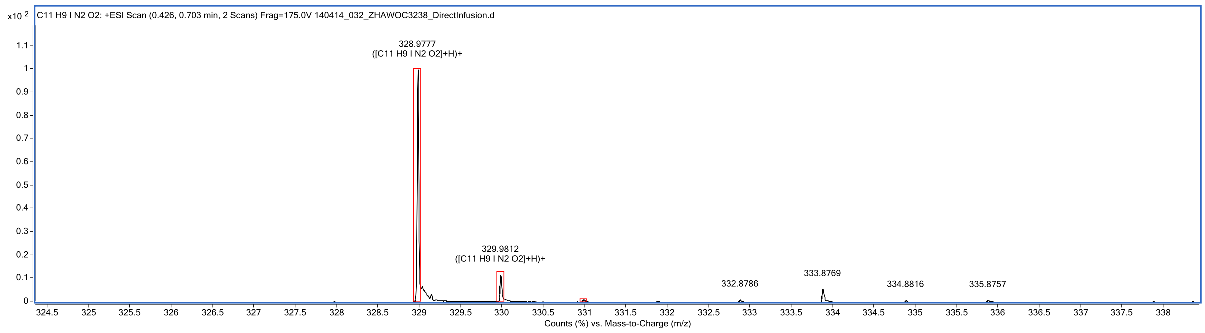

Best	ID Source	Formula	m/z	Species	Mass	Mass (MFG)	Score	Diff (ppm)
TRUE	MFG	C11 H9 I N2 O2	328.9777	(M+H)+	327.9705	327.9709	98.84	1.1

4-[(5-Iodo-2,4-dioxo-1,2,3,4-tetrahydropyrimidin-1-yl)methyl]benzoic acid (5b)

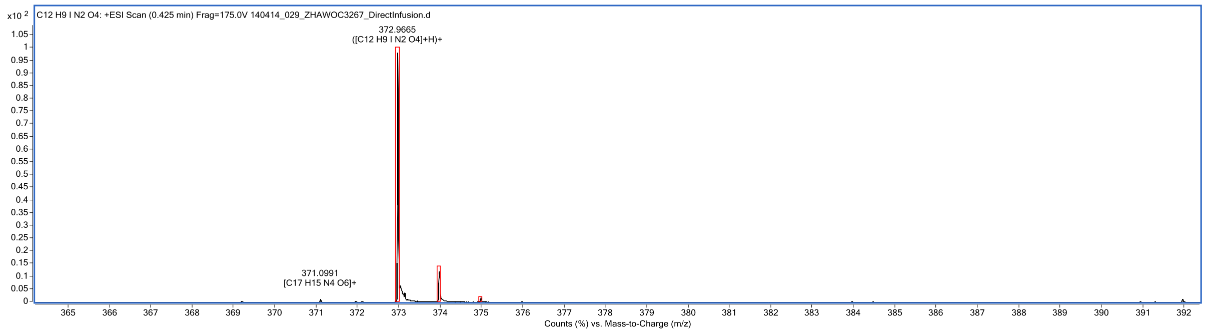

Best	ID Source	Formula	m/z	Species	Mass	Mass (MFG)	Score	Diff (ppm)
TRUE	MFG	C12 H9 I N2 O4	372.9665	(M+H)+	371.9593	371.9607	93.31	3.7

Methyl 4-[3-(aminomethyl)phenoxy]butanoate (7f)

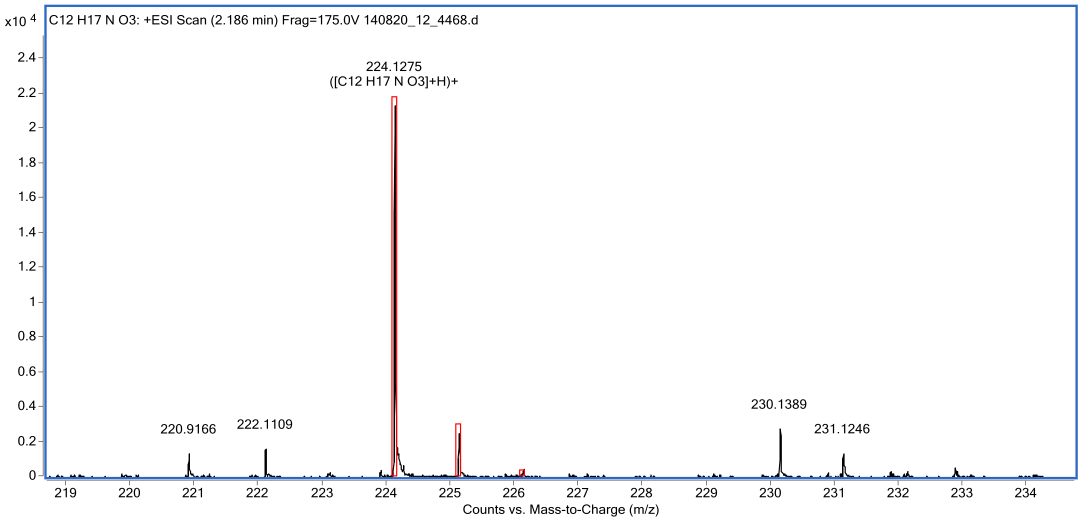

Best	ID Source	Formula	m/z	Species	Mass	Mass (MFG)	Score	Diff (ppm)
TRUE	MFG	C12 H17 N O3	224.1275	(M+H)+	223.1203	223.1208	96.25	2.65

N-Benzylprop-2-ynamide (8a)

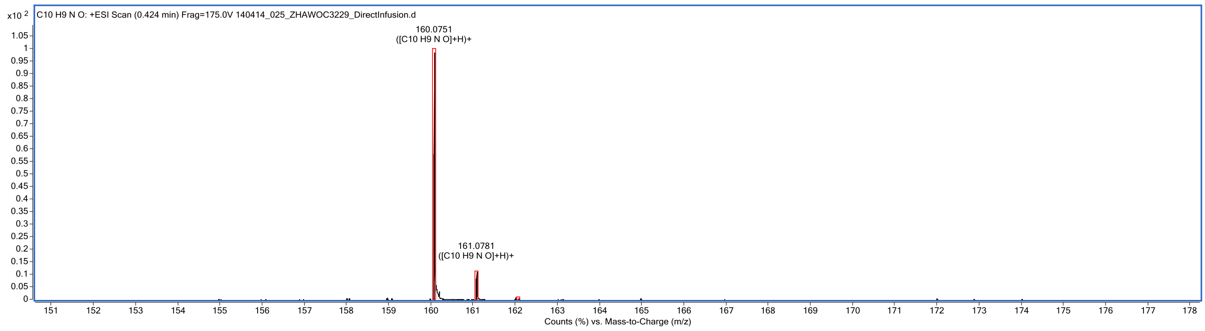

Best	ID Source	Formula	m/z	Species	Mass	Mass (MFG)	Score	Diff (ppm)
TRUE	MFG	C10 H9 N O	160.0751	(M+H)+	159.0678	159.0684	97.82	3.82

N-[(2-Fluorophenyl)methyl]prop-2-ynamide (8)

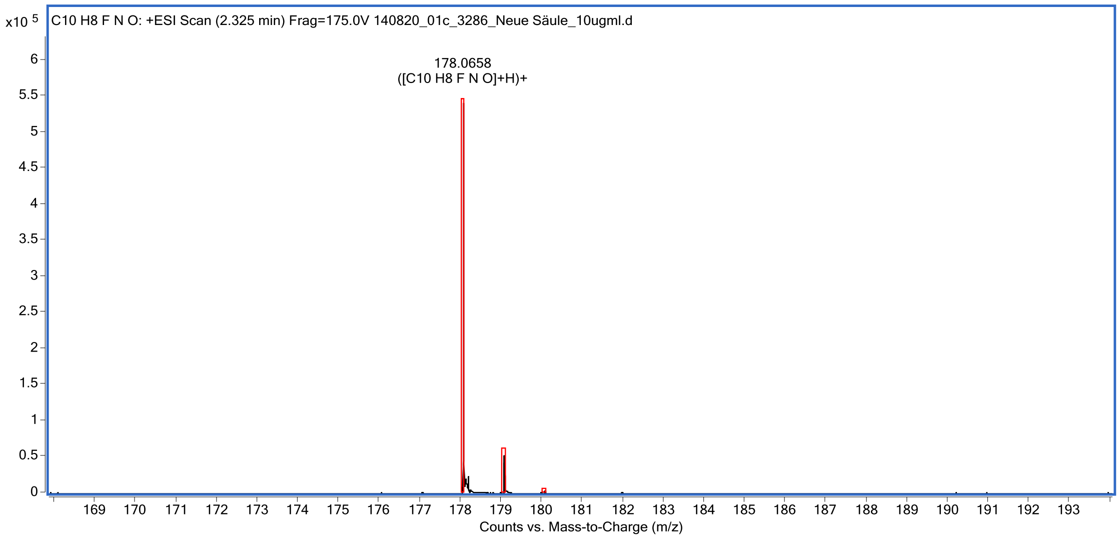

Best	ID Source	Formula	m/z	Species	Mass	Mass (MFG)	Score	Diff (ppm)
TRUE	MFG	C10 H8 F N O	178.0658	(M+H)+	177.0586	177.0590	98.27	2.43

N-[(3-Fluorophenyl)methyl]prop-2-ynamide (8c)

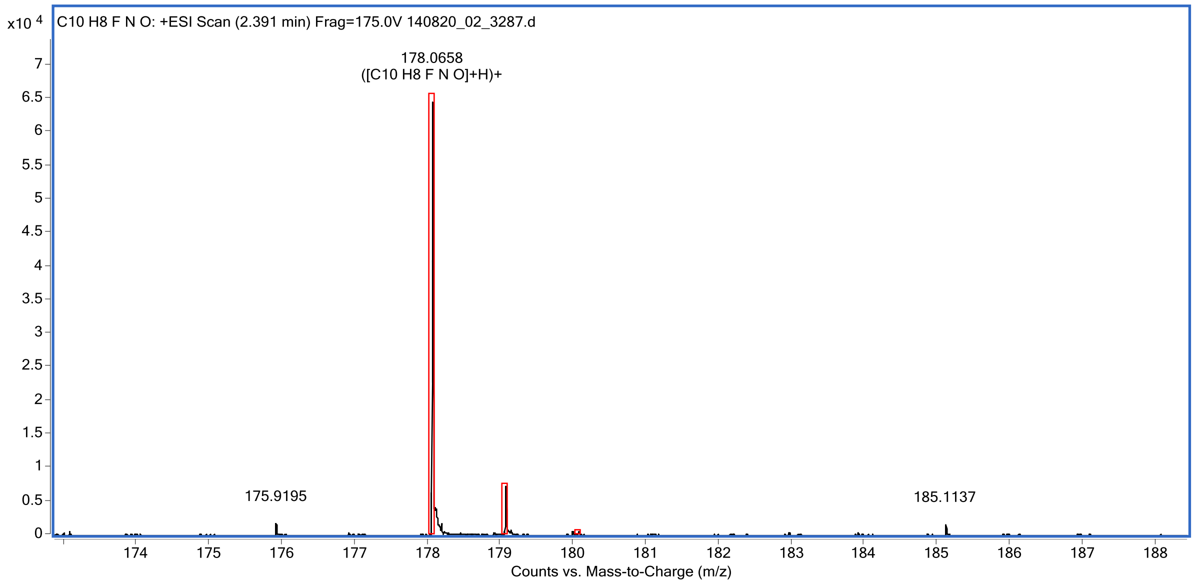

Best	ID Source	Formula	m/z	Species	Mass	Mass (MFG)	Score	Diff (ppm)
TRUE	MFG	C10 H8 F N O	178.0658	(M+H)+	177.0586	177.0590	97.07	2.11

N-[(4-Fluorophenyl)methyl]prop-2-ynamide (8d)

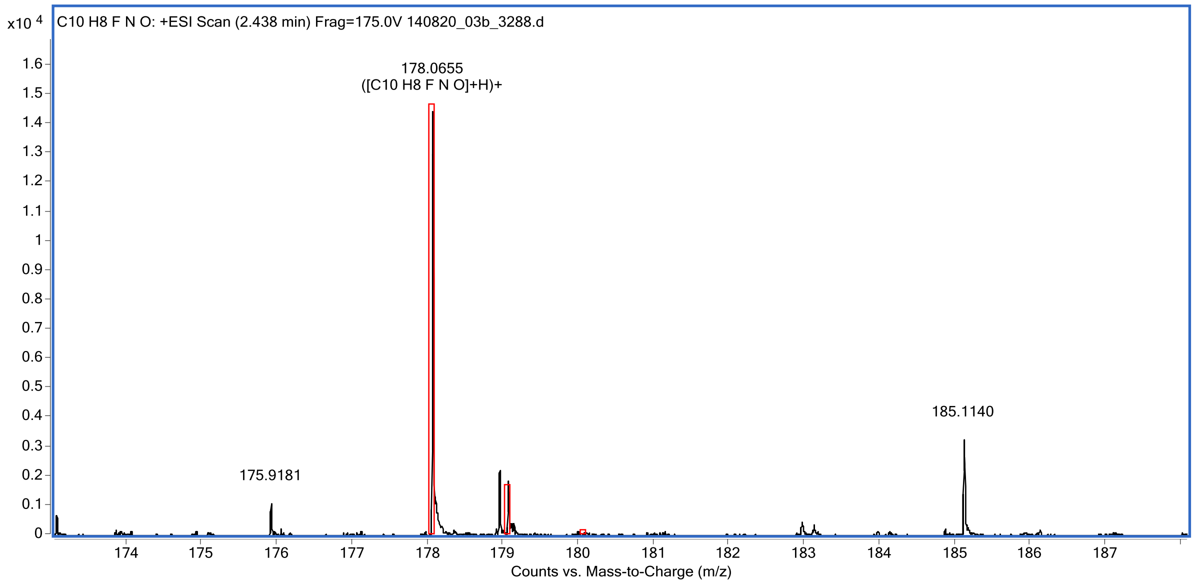

Best	ID Source	Formula	m/z	Species	Mass	Mass (MFG)	Score	Diff (ppm)
TRUE	MFG	C10 H8 F N O	178.0655	(M+H)+	177.0583	177.0590	96.29	3.93

N-[(3-Methoxyphenyl)methyl]prop-2-ynamide (8e)

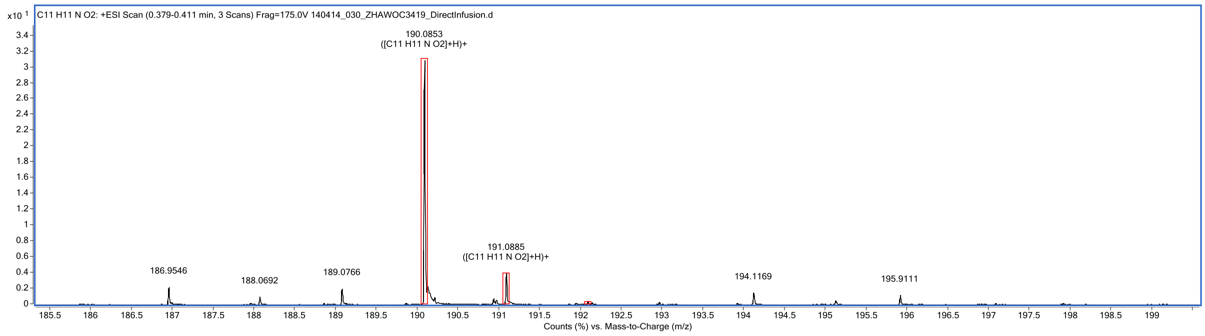

Best	ID Source	Formula	m/z	Species	Mass	Mass (MFG)	Score	Diff (ppm)
TRUE	MFG	C11 H11 N O2	190.0853	(M+H)+	189.0780	189.0790	95.56	5.02

Methyl 4-[3-(prop-2-ynamidomethyl)phenoxy]butanoate (8f)

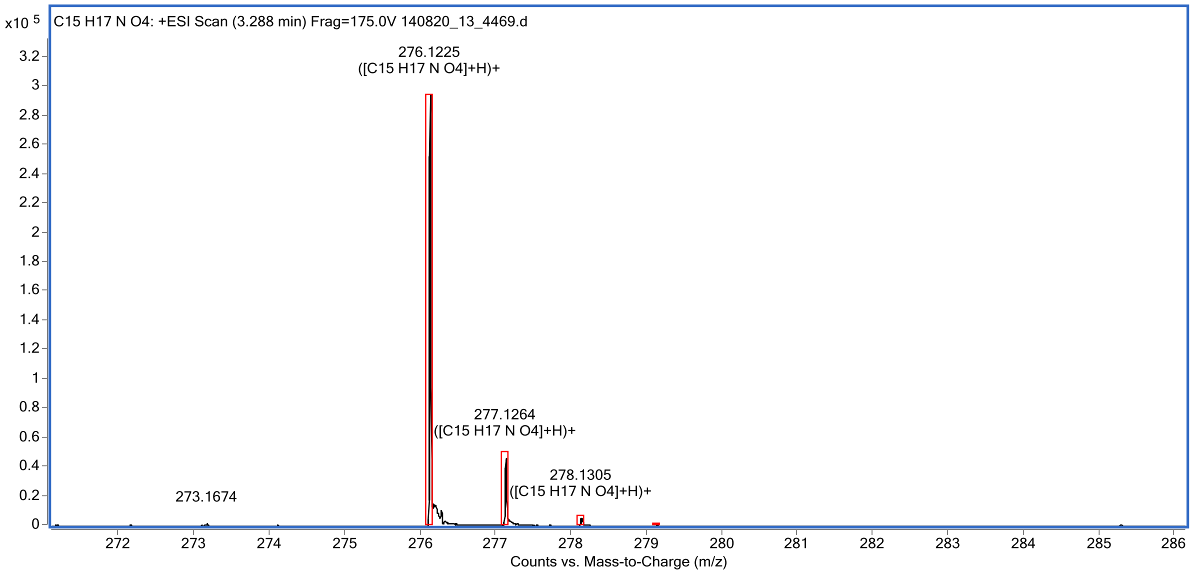

Best	ID Source	Formula	m/z	Species	Mass	Mass (MFG)	Score	Diff (ppm)
TRUE	MFG	C15 H17 N O4	276.1225	(M+H)+	275.1153	275.1158	98.18	1.59

4-[3-(Prop-2-ynamidomethyl)phenoxy]butanoic acid (8f)

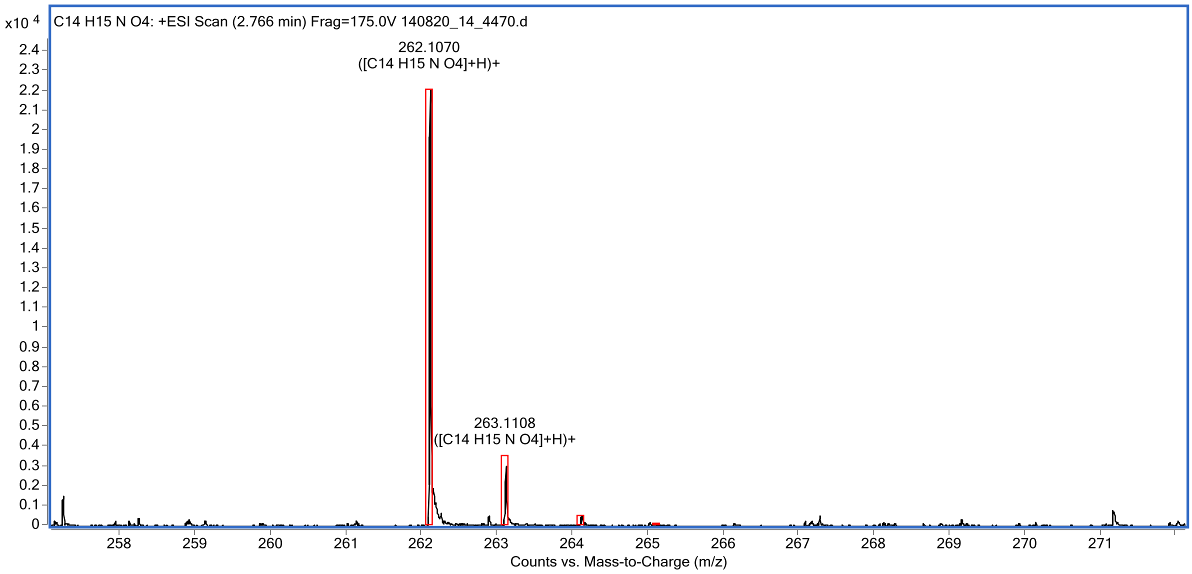

Best	ID Source	Formula	m/z	Species	Mass	Mass (MFG)	Score	Diff (ppm)
TRUE	MFG	C14 H15 N O4	262.1070	(M+H)+	261.0998	261.1001	97.48	1.09

N-Benzyl-3-(1-benzyl-2,4-dioxo-1,2,3,4-tetrahydropyrimidin-5-yl)prop-2-ynamide (2)

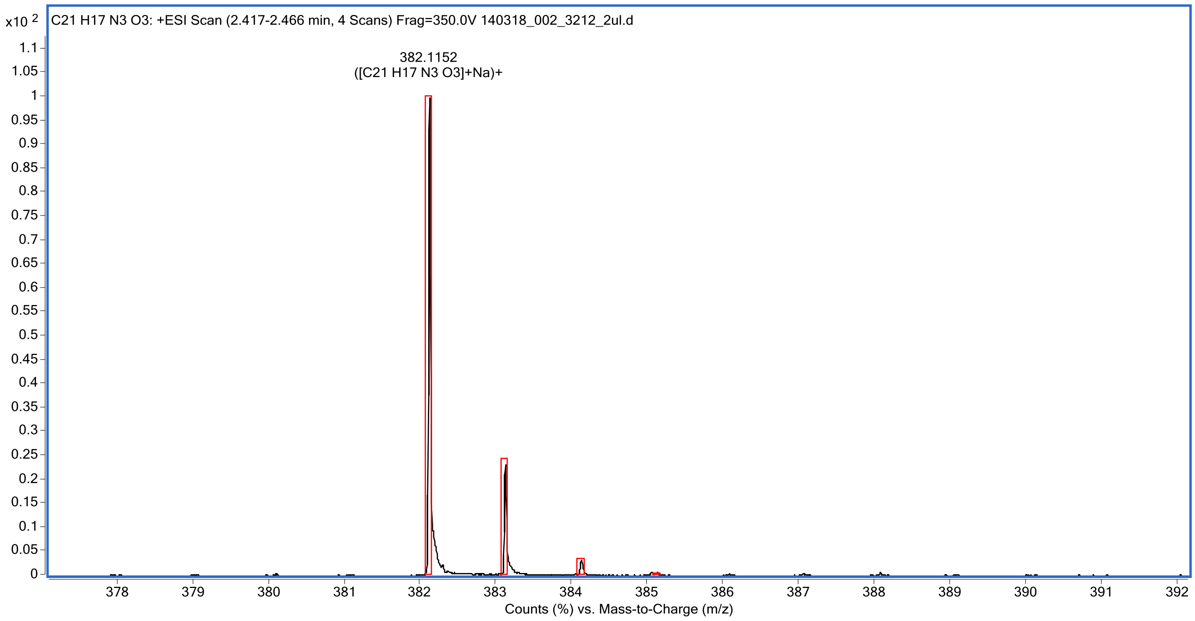

Best	ID Source	Formula	m/z	Species	Mass	Mass (MFG)	Score	Diff (ppm)
TRUE	MFG	C21 H17 N3 O3	382.1152	(M+Na)+	359.1259	359.1270	96.22	3.13

4-{[5-(2-{[(3-methoxyphenyl)methyl]carbamoyl}eth-1-yn-1-yl)-2,4-dioxo-1,2,3,4-tetrahydropyrimidin-1-yl]methyl}benzoic acid (3)

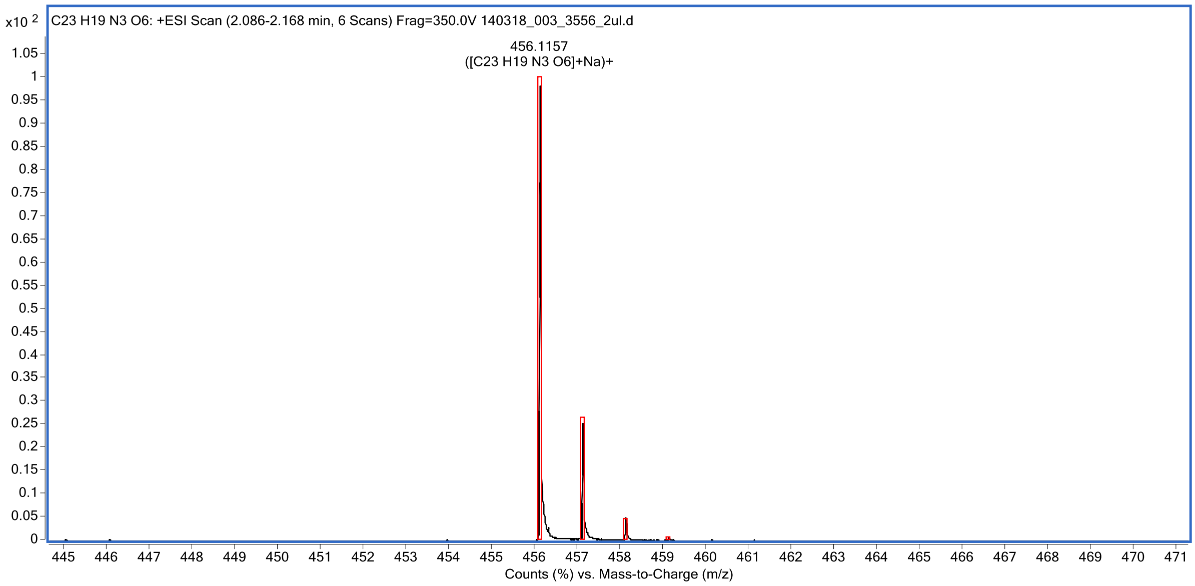

Best	ID Source	Formula	m/z	Species	Mass	Mass (MFG)	Score	Diff (ppm)
TRUE	MFG	C23 H19 N3 O6	456.1157	(M+Na)+	433.1264	433.1274	97.53	2.23

4-({5-[2-({[3-(3-Carboxypropoxy)phenyl]methyl}carbamoyl)eth-1-yn-1-yl]-2,4-dioxo-1,2,3,4-tetrahydropyrimidin-1-yl}methyl)benzoic acid (4)

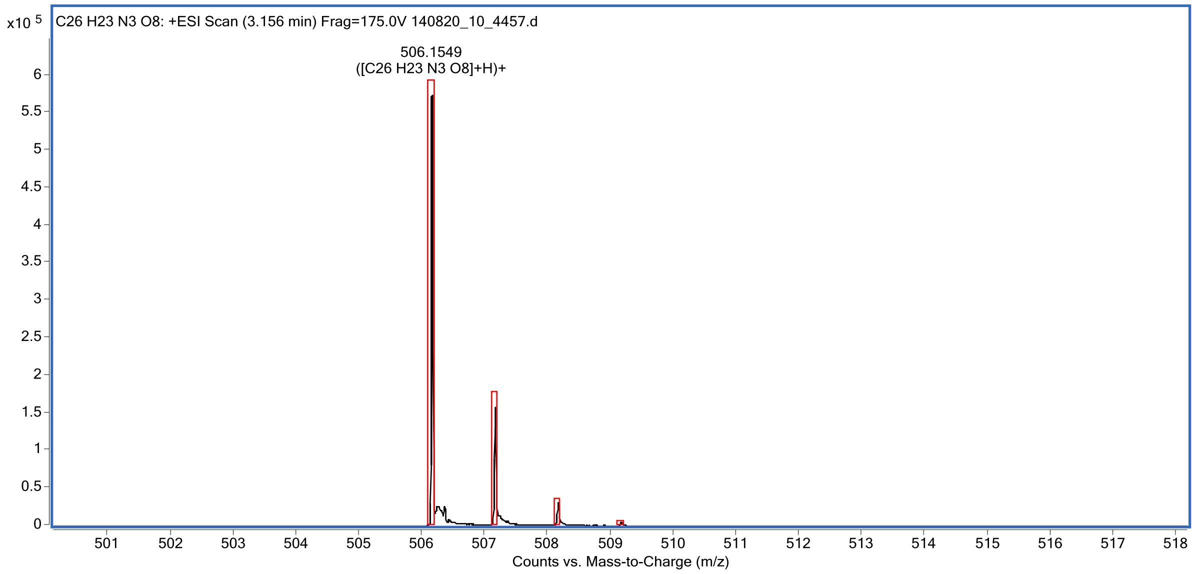

Best	ID Source	Formula	m/z	Species	Mass	Mass (MFG)	Score	Diff (ppm)
TRUE	MFG	C26 H23 N3 O8	506.1549	(M+H)+	505.1475	505.1485	96.51	1.95

3-(1-Benzyl-2,4-dioxo-1,2,3,4-tetrahydropyrimidin-5-yl)-N-[(2-fluorophenyl)methyl]prop-2-ynamide (9a)

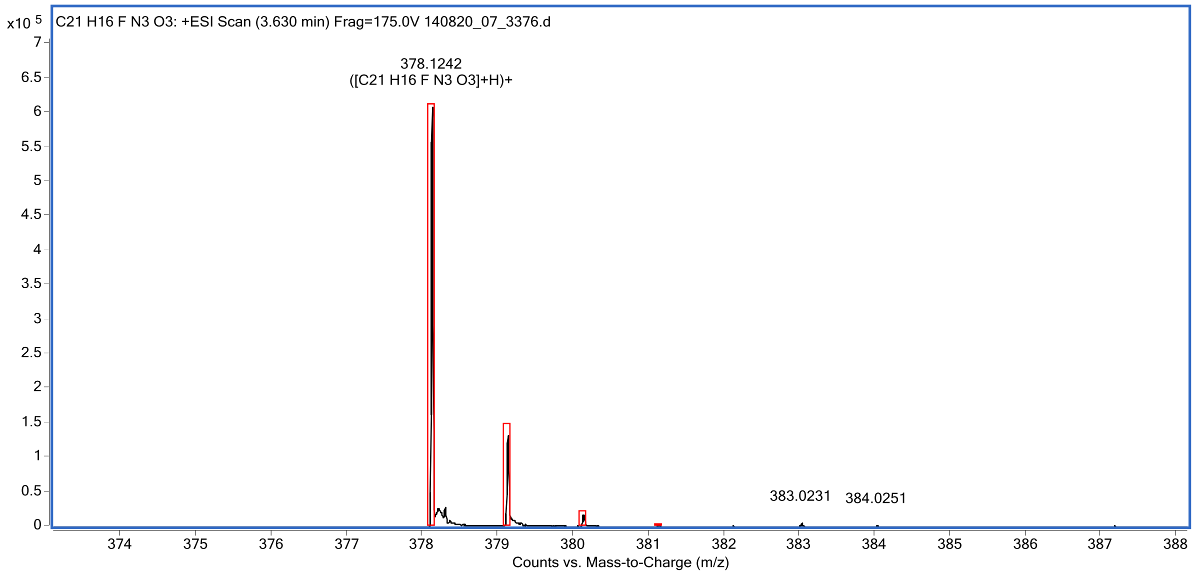

Best	ID Source	Formula	m/z	Species	Mass	Mass (MFG)	Score	Diff (ppm)
TRUE	MFG	C21 H16 F N3 O3	378.1242	(M+H)+	377.1168	377.1176	97.39	1.92

3-(1-Benzyl-2,4-dioxo-1,2,3,4-tetrahydropyrimidin-5-yl)-N-[(3-fluorophenyl)methyl]prop-2-ynamide (9b)

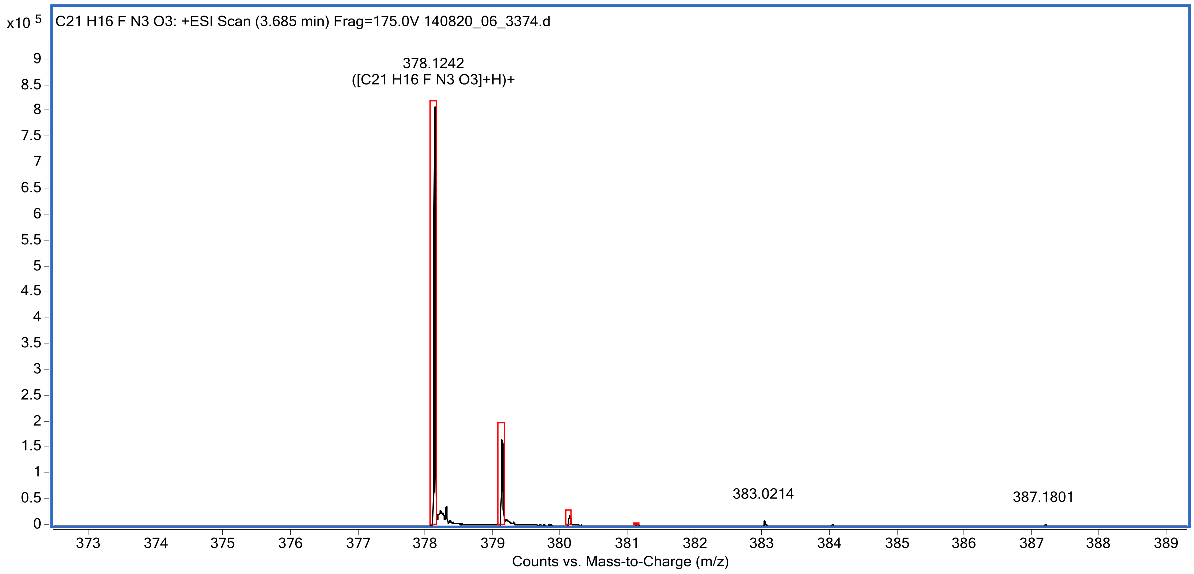

Best	ID Source	Formula	m/z	Species	Mass	Mass (MFG)	Score	Diff (ppm)
TRUE	MFG	C21 H16 F N3 O3	378.1242	(M+H)+	377.1168	377.1176	96.18	1.92

3-(1-Benzyl-2,4-dioxo-1,2,3,4-tetrahydropyrimidin-5-yl)-N-[(4-fluorophenyl)methyl]prop-2-ynamide (9c)

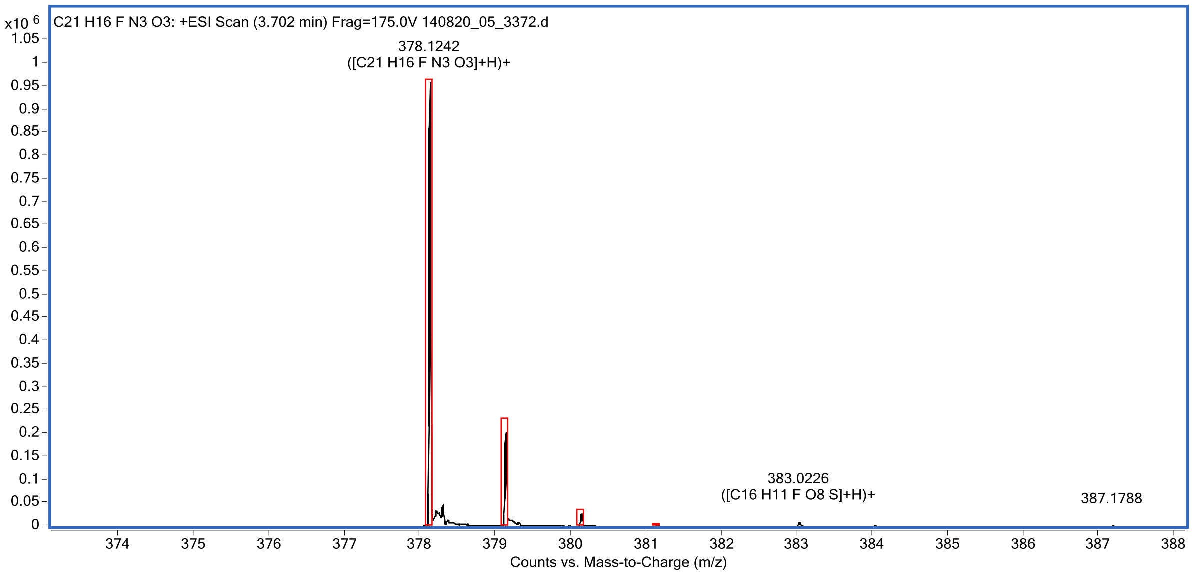

Best	ID Source	Formula	m/z	Species	Mass	Mass (MFG)	Score	Diff (ppm)
TRUE	MFG	C21 H16 F N3 O3	378.1242	(M+H)+	377.1168	377.1176	96.45	1.95

IR spectra

1-Benzyl-5-iodo-1,2,3,4-tetrahydropyrimidine-2,4-dione (5a)

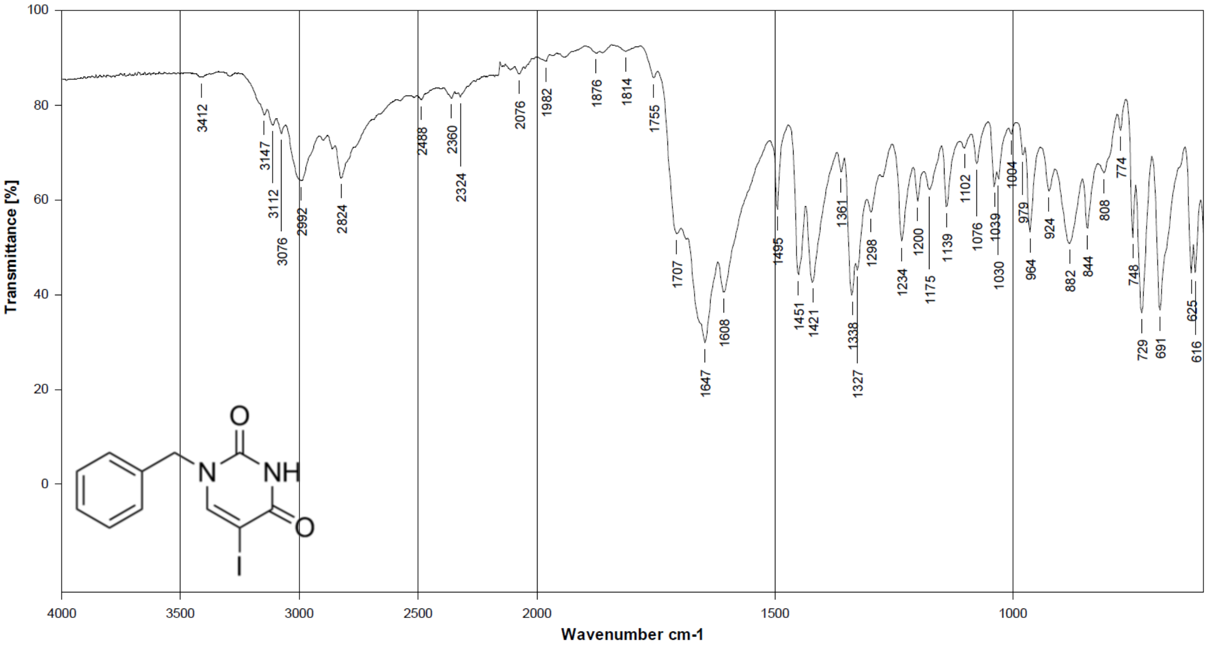

4-[(5-Iodo-2,4-dioxo-1,2,3,4-tetrahydropyrimidin-1-yl)methyl]benzoic acid (5b)

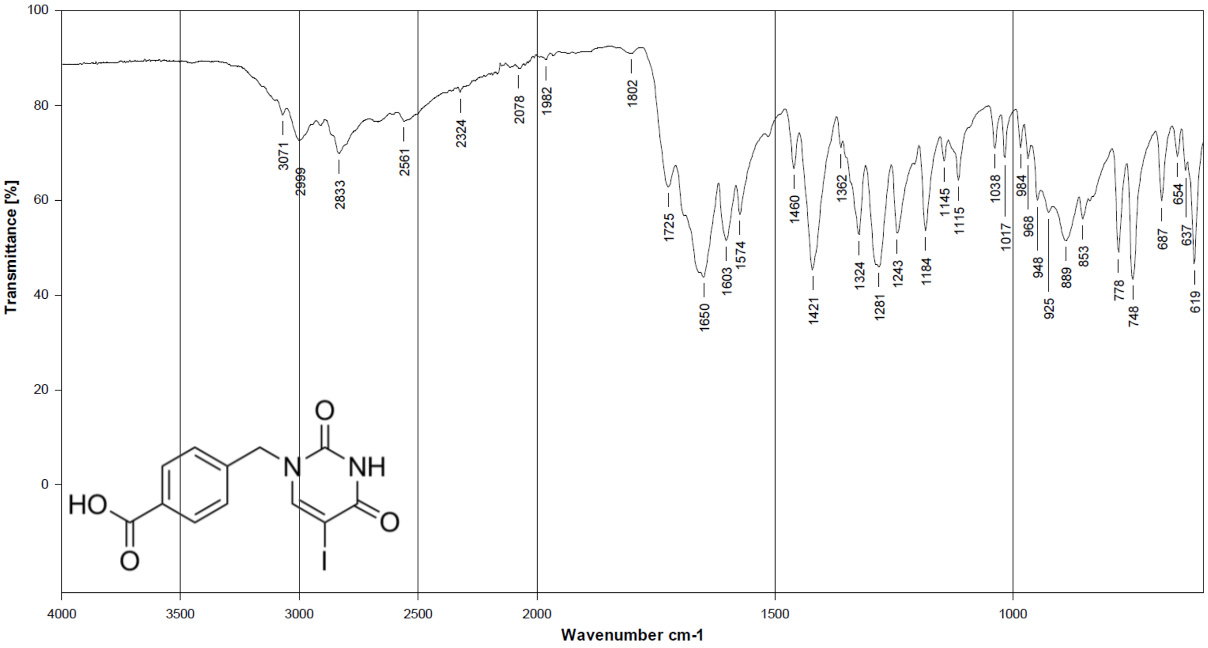

Methyl 4-(3-cyanophenoxy)butanoate (6)

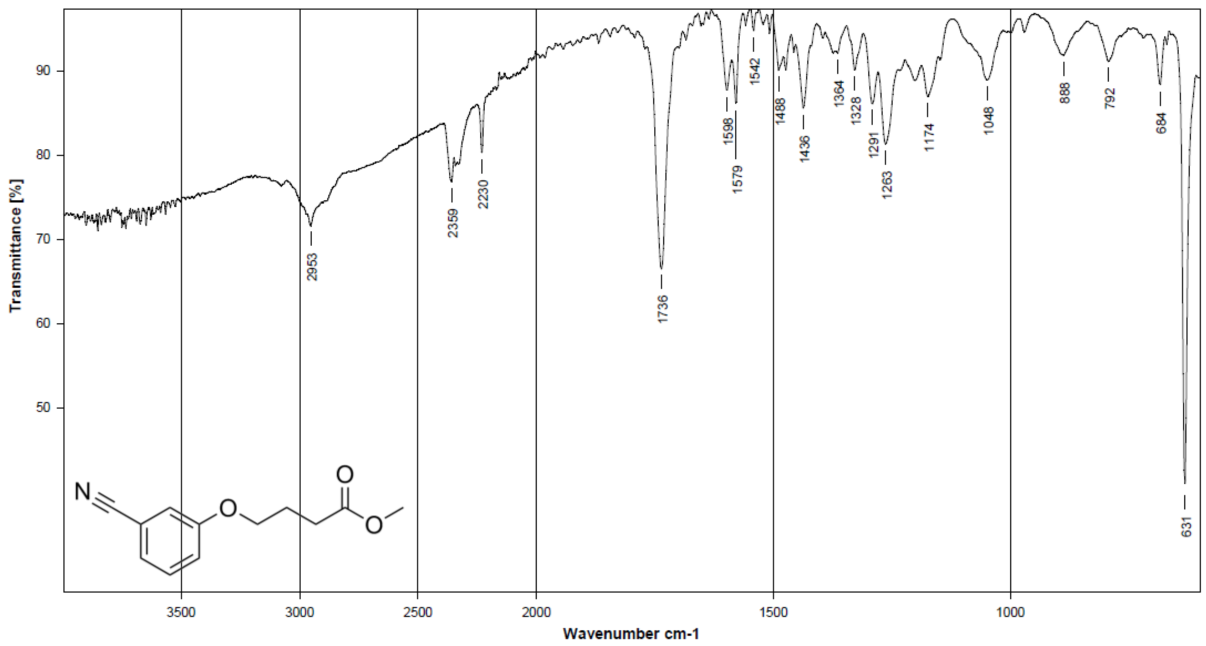

N-Benzylprop-2-ynamide (8a)

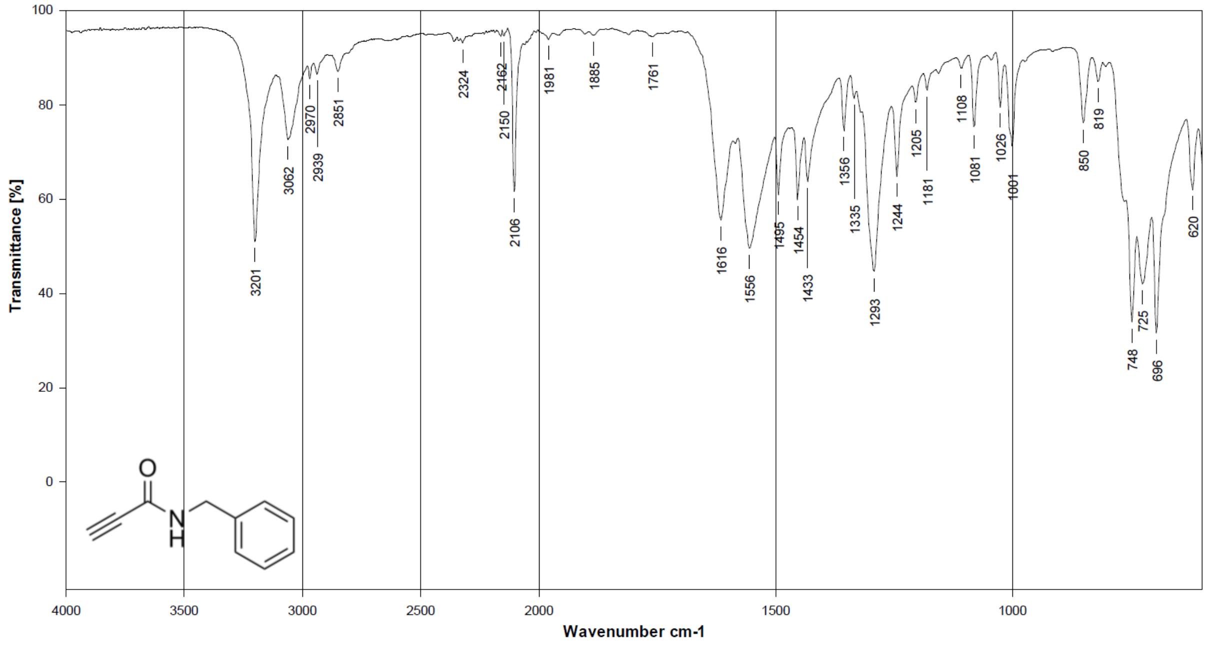

N-[(2-Fluorophenyl)methyl]prop-2-ynamide (8b)

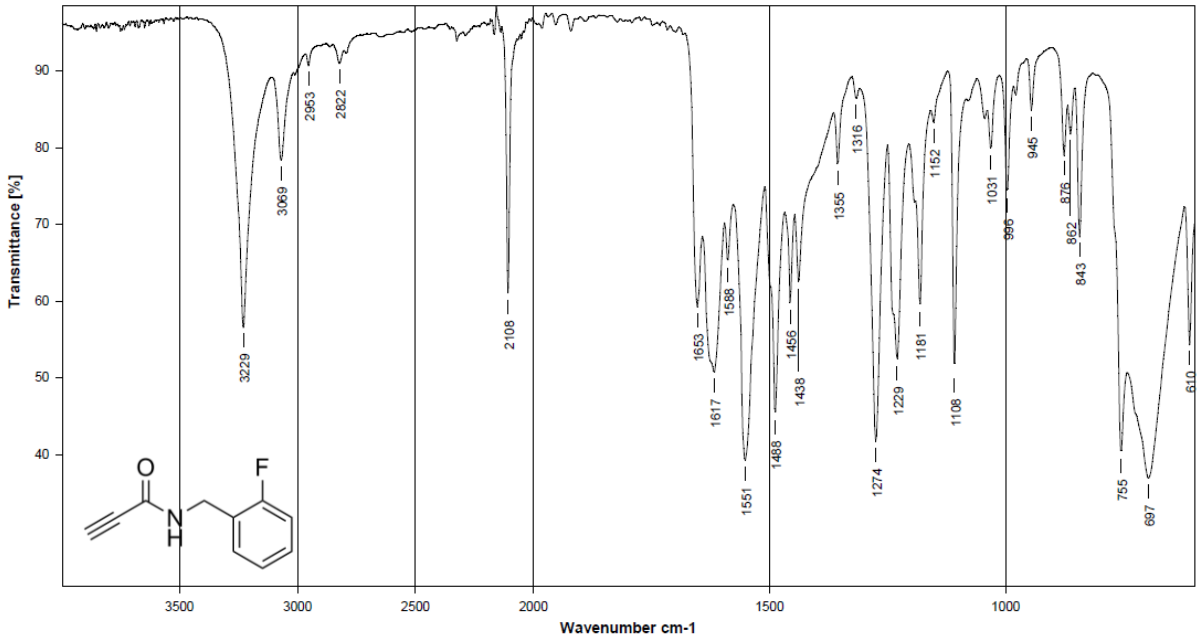

N-[(3-Fluorophenyl)methyl]prop-2-ynamide (8c)

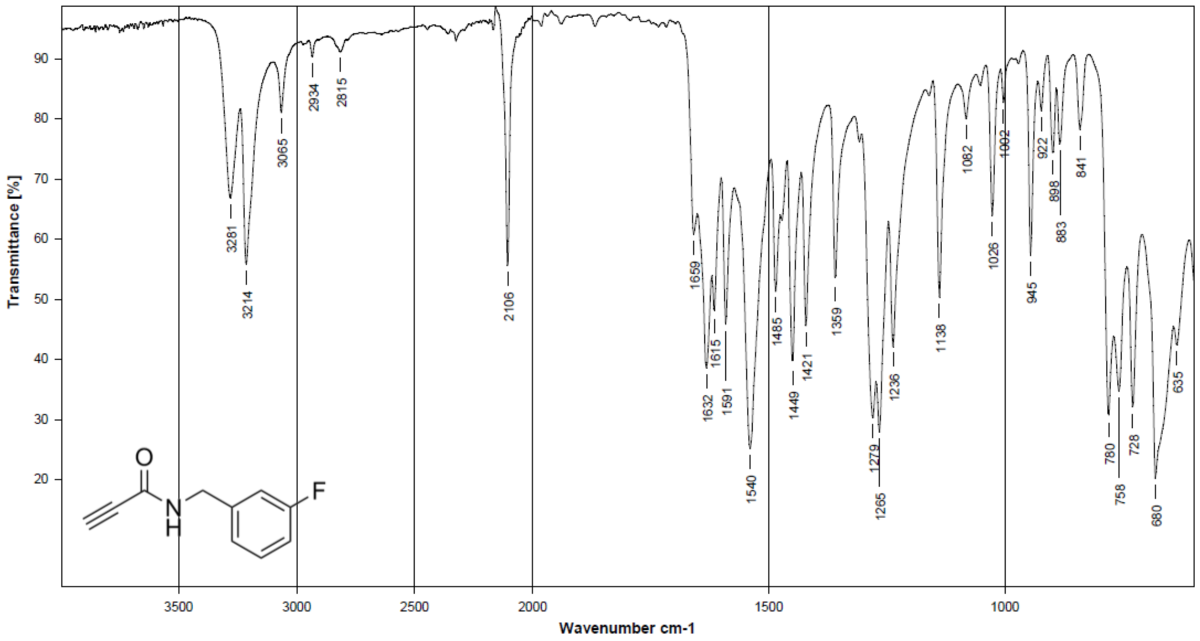

***N*-[(4-Fluorophenyl)methyl]prop-2-ynamide (8d)**

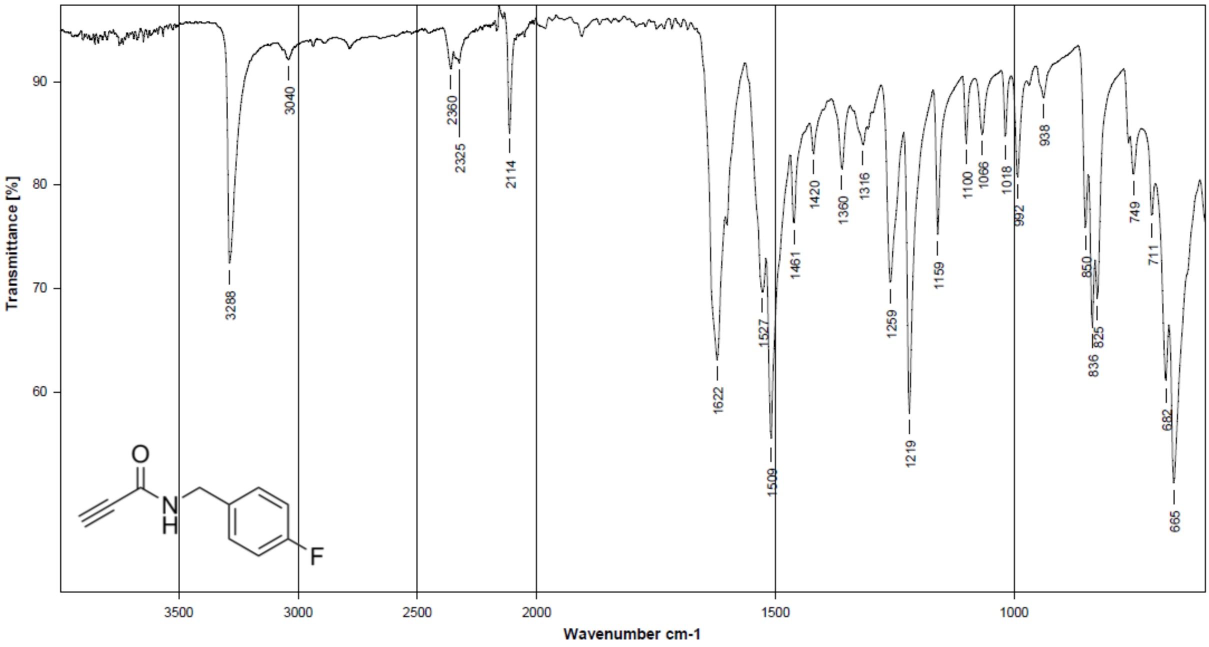

***N*-[(3-Methoxyphenyl)methyl]prop-2-ynamide (8e)**

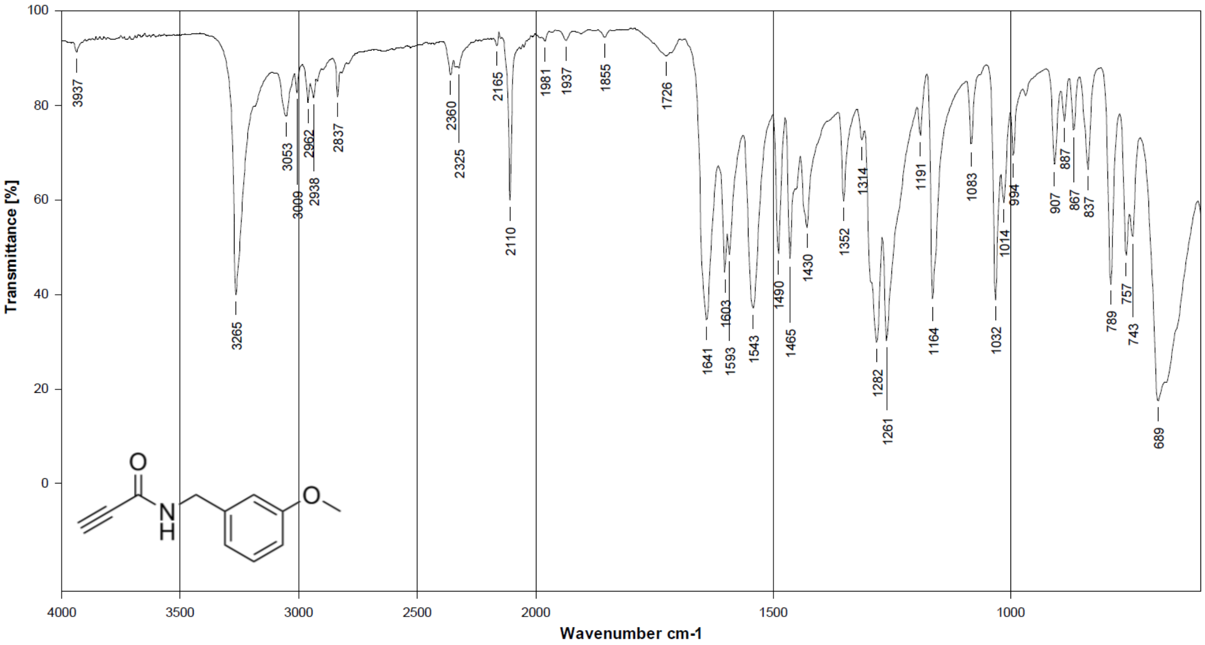

Methyl 4-[3-(prop-2-ynamidomethyl)phenoxy]butanoate (8f)

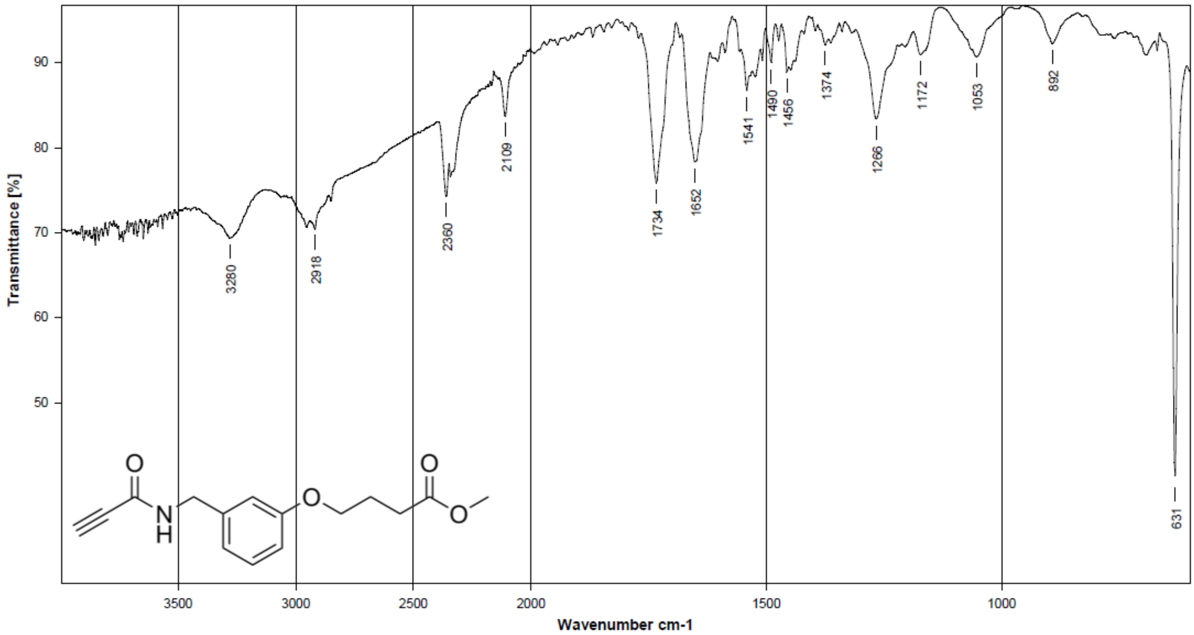

4-[3-(Prop-2-ynamidomethyl)phenoxy]butanoic acid (8g)

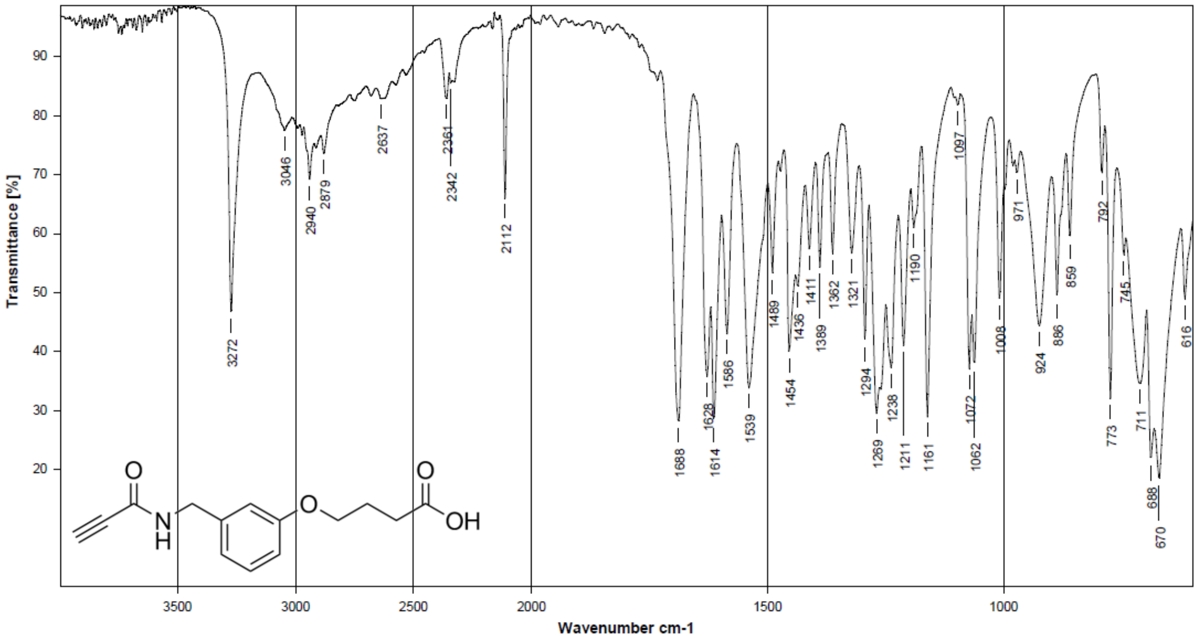

N-Benzyl-3-(1-benzyl-2,4-dioxo-1,2,3,4-tetrahydropyrimidin-5-yl)prop-2-ynamide (2)

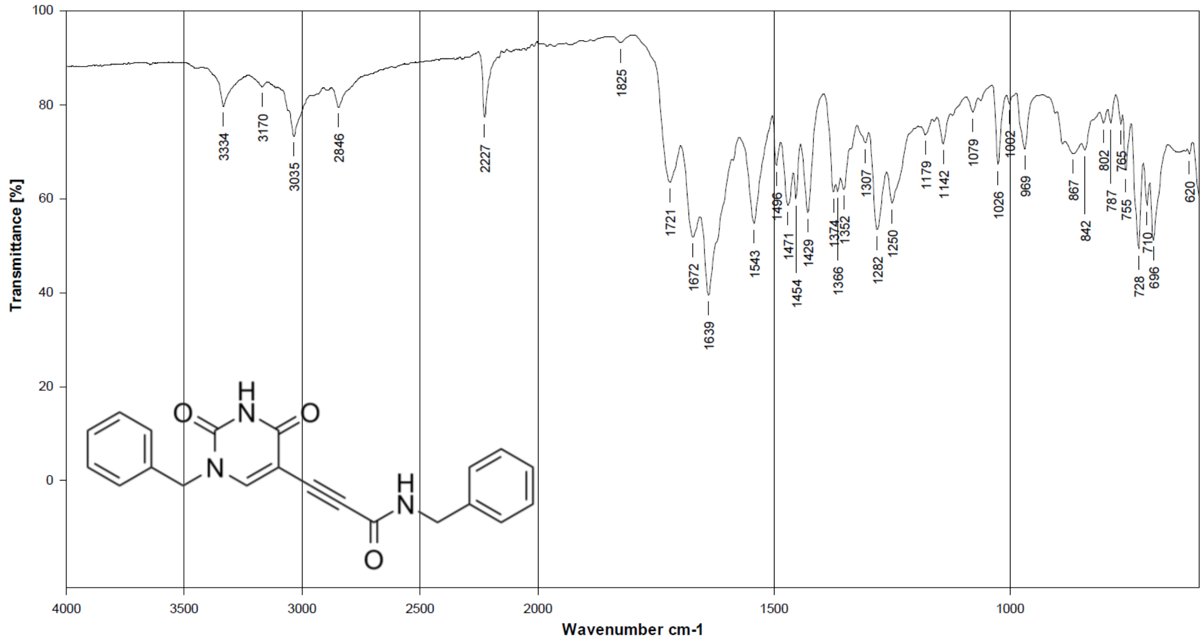

4-{[5-(2-{[(3-methoxyphenyl)methyl]carbamoyl}eth-1-yn-1-yl)-2,4-dioxo-1,2,3,4-tetrahydropyrimidin-1-yl]methyl}benzoic acid (3)

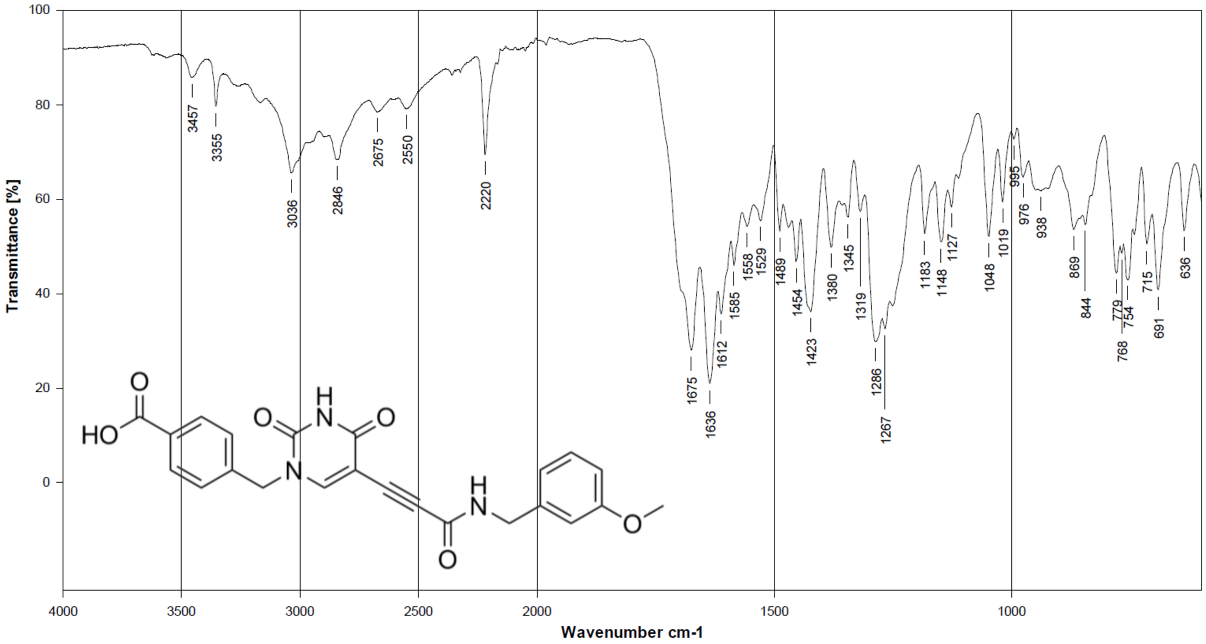

4-({5-[2-({[3-(3-Carboxypropoxy)phenyl]methyl}carbamoyl)eth-1-yn-1-yl]-2,4-dioxo-1,2,3,4-tetrahydropyrimidin-1-yl}methyl)benzoic acid (4)

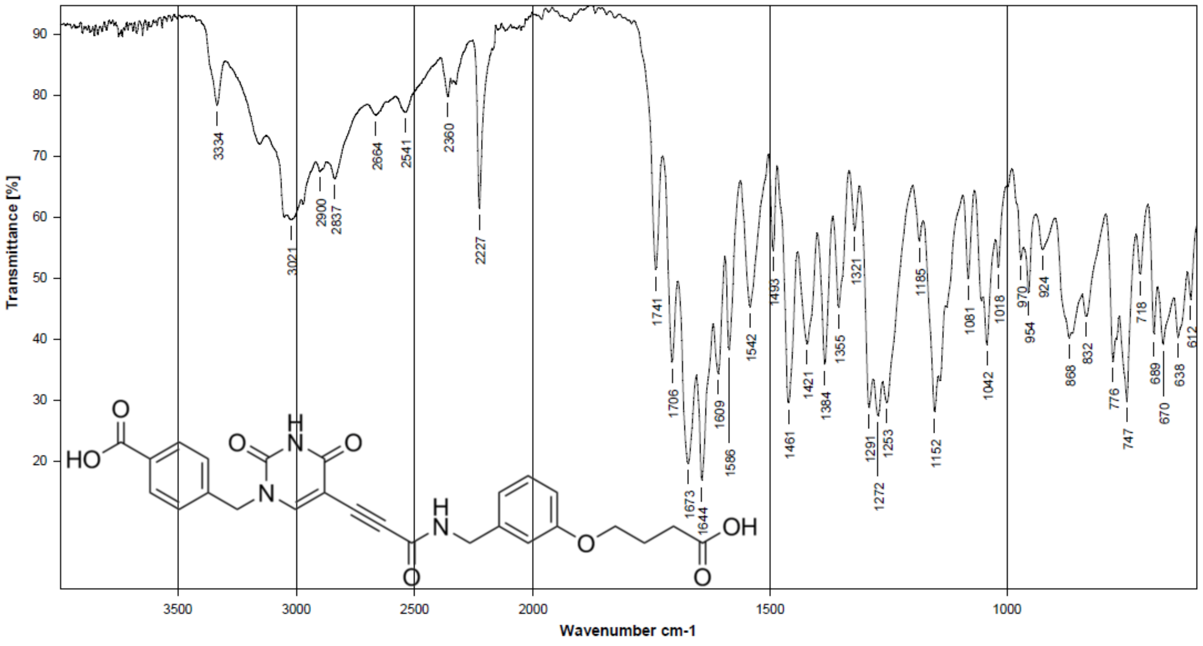

3-(1-Benzyl-2,4-dioxo-1,2,3,4-tetrahydropyrimidin-5-yl)-N-[(2-fluorophenyl)methyl]prop-2-ynamide (9a)

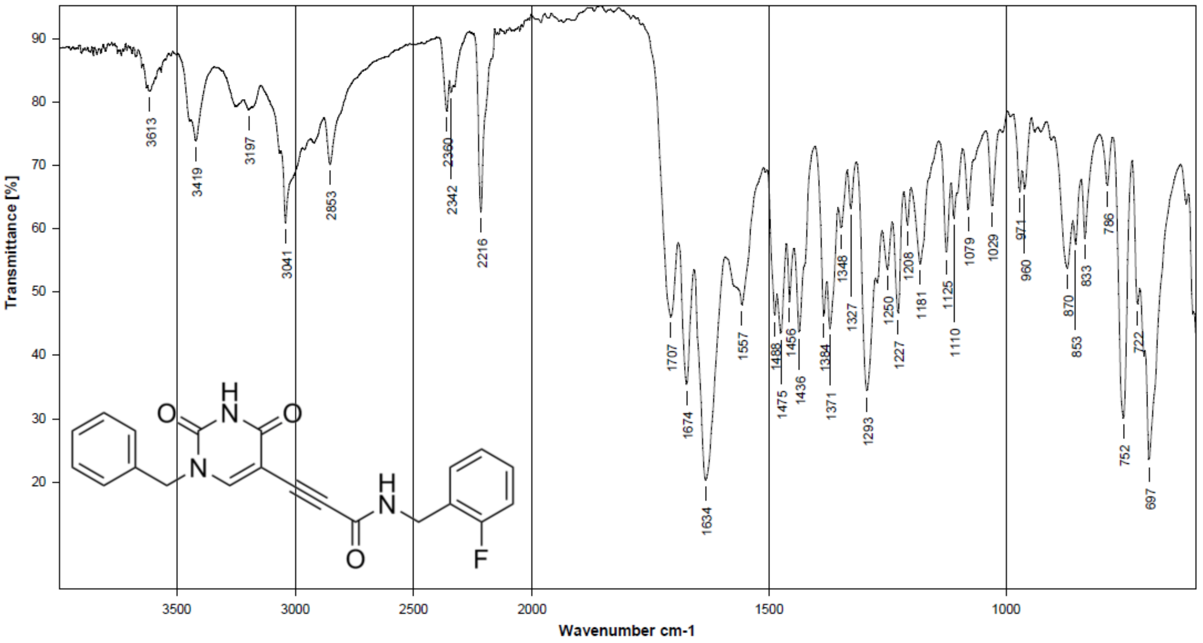

3-(1-Benzyl-2,4-dioxo-1,2,3,4-tetrahydropyrimidin-5-yl)-N-[(3-fluorophenyl)methyl]prop-2-ynamide (9b)

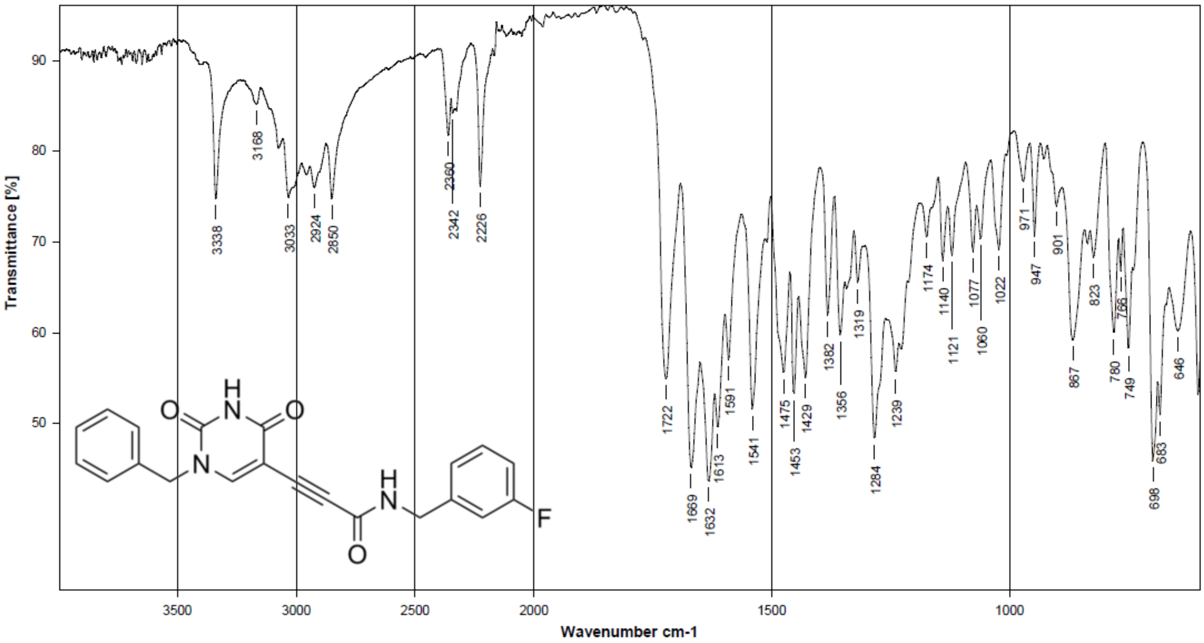

3-(1-Benzyl-2,4-dioxo-1,2,3,4-tetrahydropyrimidin-5-yl)-N-[(4-fluorophenyl)methyl]prop-2-ynamide (9c)

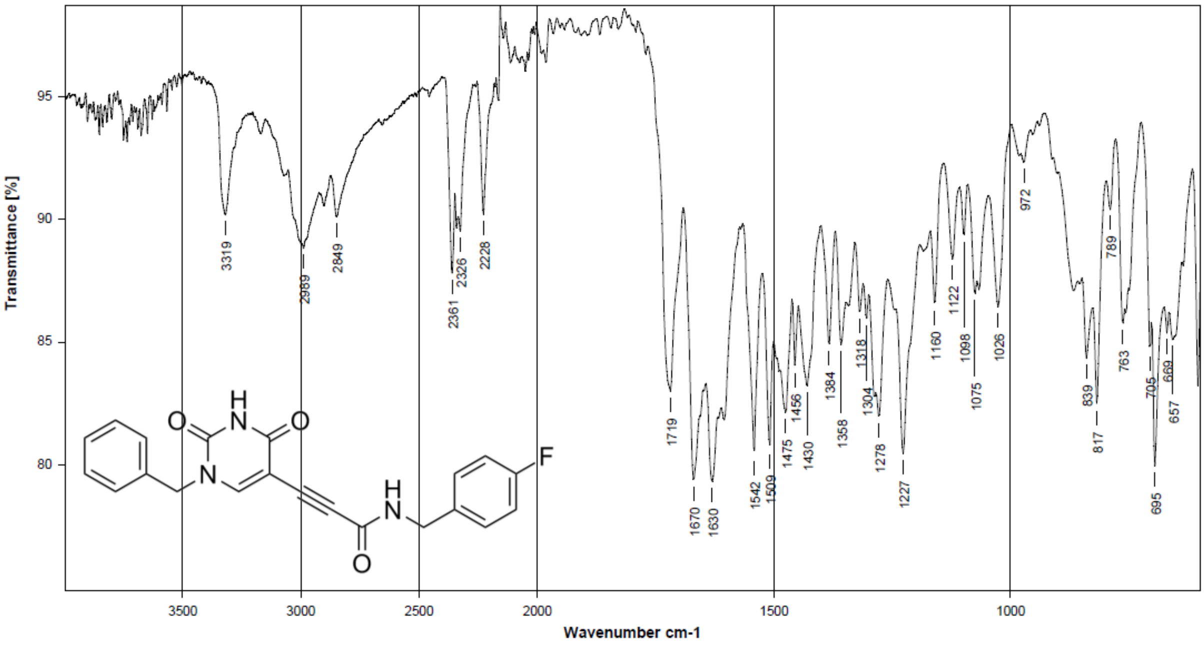

HPLC – Purity

N-Benzyl-3-(1-benzyl-2,4-dioxo-1,2,3,4-tetrahydropyrimidin-5-yl)prop-2-ynamide (2)

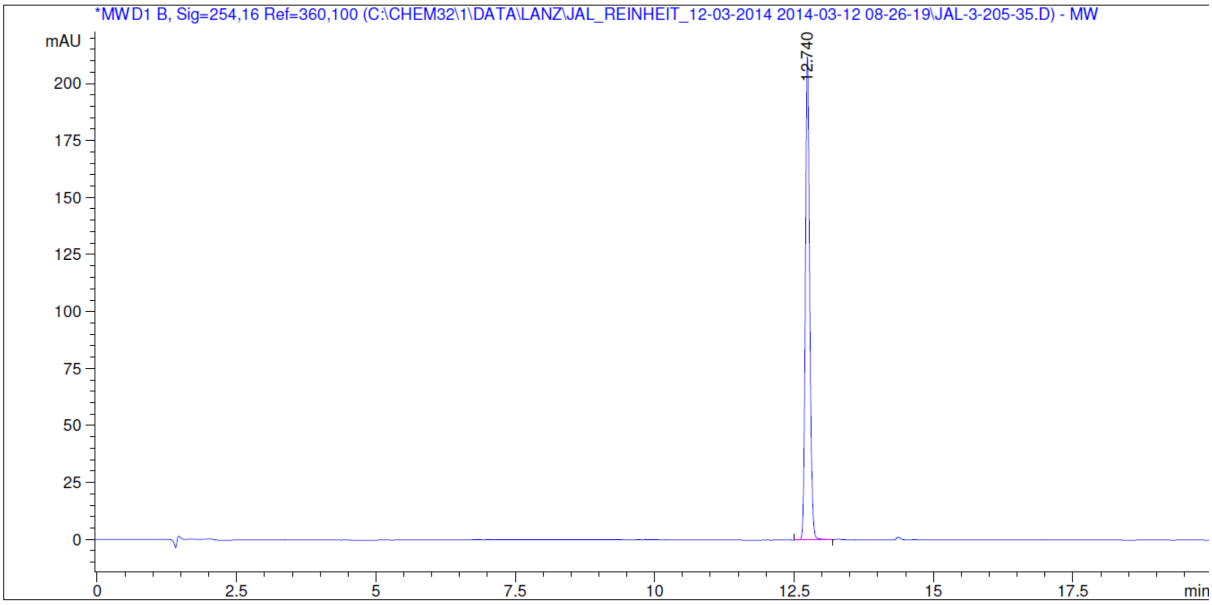


```
Signal 1: MWD1 B, Sig=254,16 Ref=360,100
 Signal has been modified after loading from rawdata file!

Peak RetTime Type  Width     Area       Height     Area
  #    [min]        [min]   [mAU*s]      [mAU]      %
----|-------|----|-------|----------|----------|--------|
   1  12.740 BB    0.0832 1151.00049  212.61676 100.0000

Totals :                  1151.00049  212.61676
```

4-{[5-(2-{[(3-methoxyphenyl)methyl]carbamoyl}eth-1-yn-1-yl)-2,4-dioxo-1,2,3,4-tetrahydropyrimidin-1-yl]methyl}benzoic acid (3)

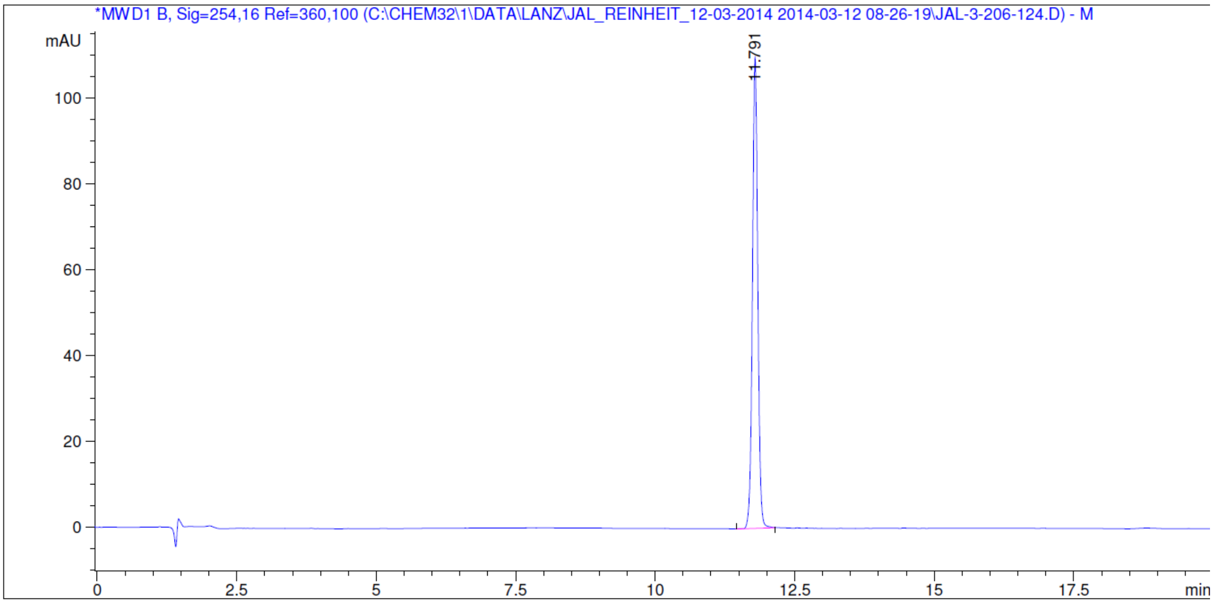


```
Peak RetTime Type  Width     Area       Height     Area
  #    [min]        [min]   [mAU*s]      [mAU]      %
----|-------|----|-------|----------|----------|--------|
   1  11.791 BB    0.0985  721.39197  109.97802 100.0000

Totals :                   721.39197  109.97802
```

4-({5-[2-({[3-(3-Carboxypropoxy)phenyl]methyl}carbamoyl)eth-1-yn-1-yl]-2,4-dioxo-1,2,3,4-tetrahydropyrimidin-1-yl}methyl)benzoic acid (4)

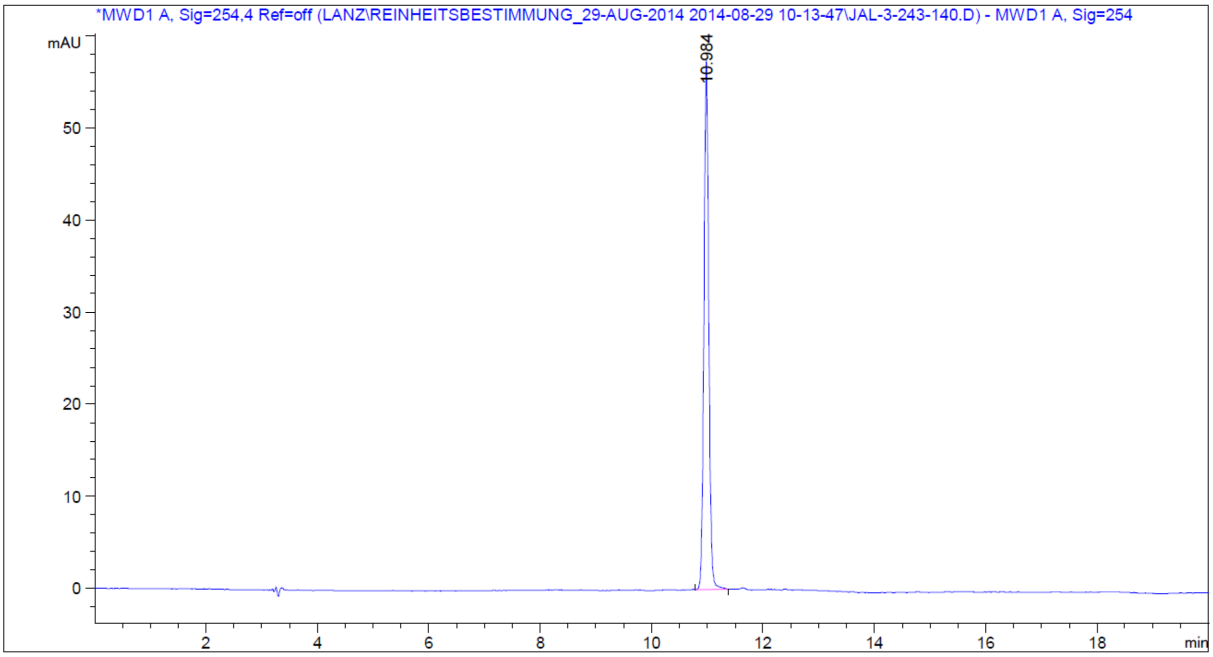

Peak #	RetTime [min]	Type	Width [min]	Area [mAU*s]	Height [mAU]	Area %
1	10.984	BB	0.0924	347.10120	57.48266	100.0000
Totals :				347.10120	57.48266	

3-(1-Benzyl-2,4-dioxo-1,2,3,4-tetrahydropyrimidin-5-yl)-N-[(2-fluorophenyl)methyl]prop-2-ynamide (9a)

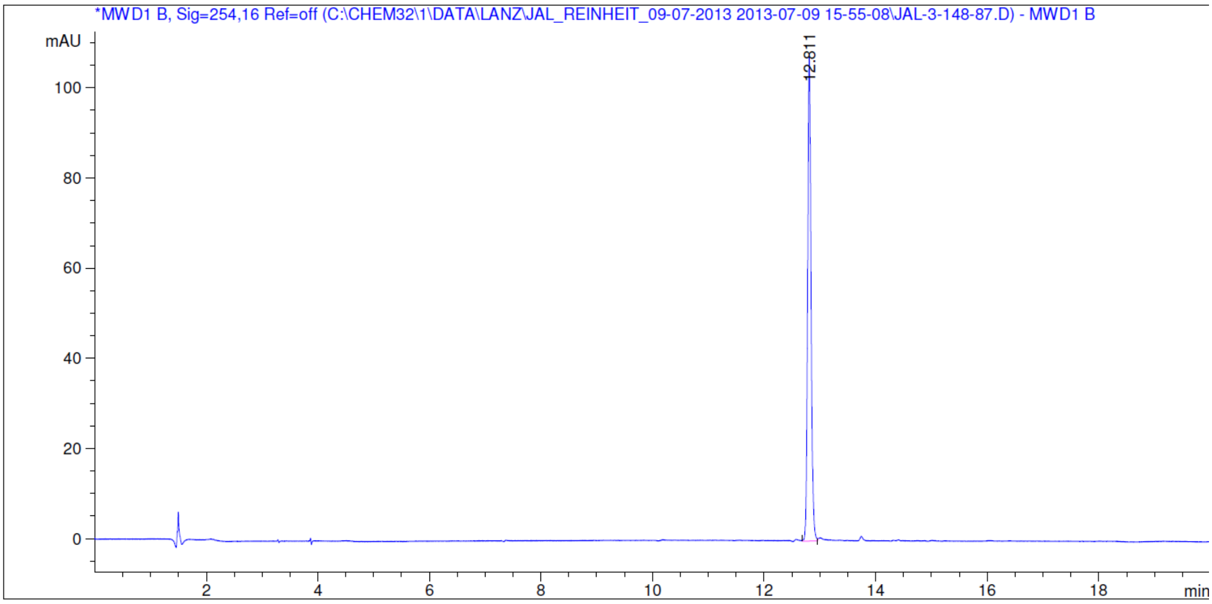

Peak #	RetTime [min]	Type	Width [min]	Area [mAU*s]	Height [mAU]	Area %
1	12.811	VV	0.0647	457.53531	107.35324	100.0000
Totals :				457.53531	107.35324	

3-(1-Benzyl-2,4-dioxo-1,2,3,4-tetrahydropyrimidin-5-yl)-N-[(3-fluorophenyl)methyl]prop-2-ynamide (9b)

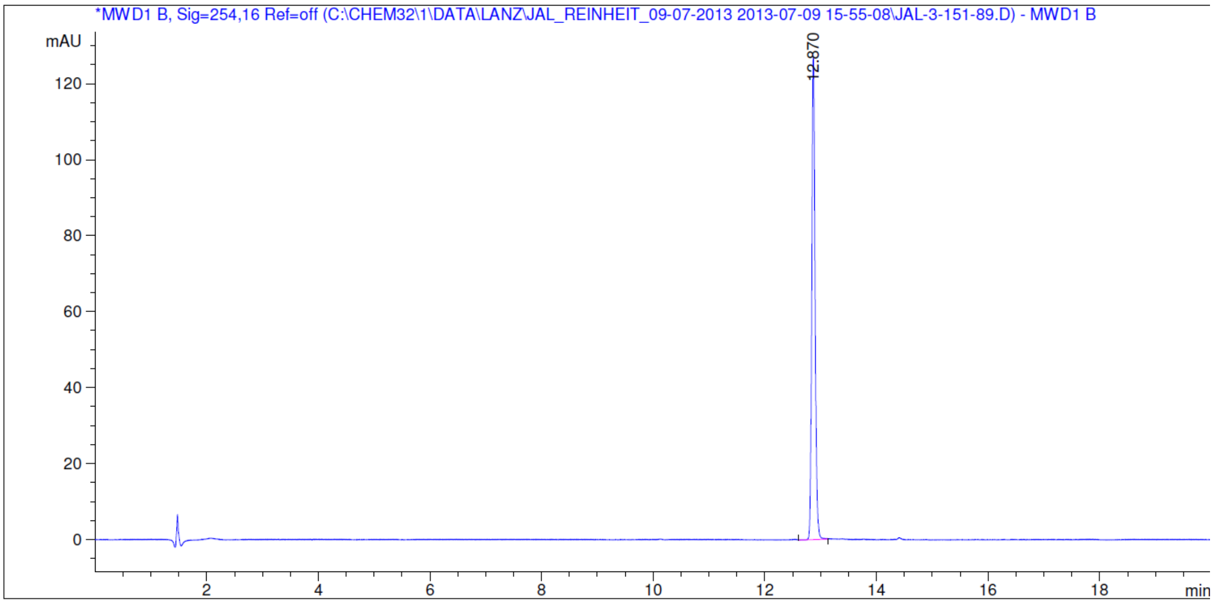

Peak #	RetTime [min]	Type	Width [min]	Area [mAU*s]	Height [mAU]	Area %
1	12.870	VB	0.0623	526.18805	126.99585	100.0000
Totals :				526.18805	126.99585	

3-(1-Benzyl-2,4-dioxo-1,2,3,4-tetrahydropyrimidin-5-yl)-N-[(4-fluorophenyl)methyl]prop-2-ynamide (9c)

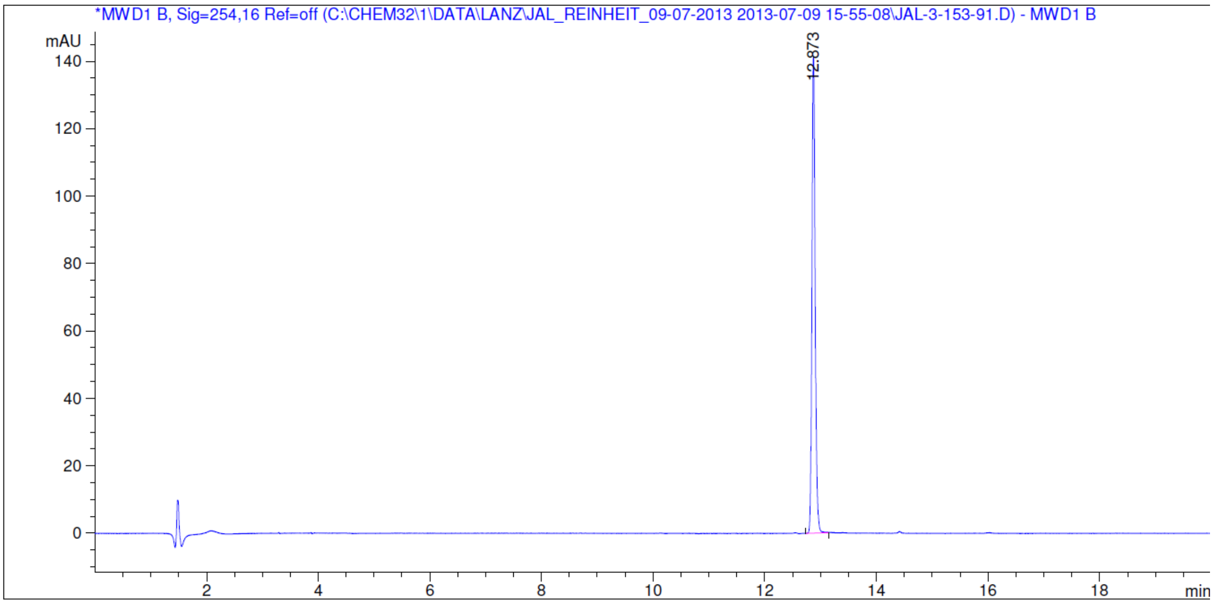

Peak #	RetTime [min]	Type	Width [min]	Area [mAU*s]	Height [mAU]	Area %
1	12.873	VB	0.0614	574.28198	141.21097	100.0000
Totals :				574.28198	141.21097	

Elemental analysis

N-Benzyl-3-(1-benzyl-2,4-dioxo-1,2,3,4-tetrahydropyrimidin-5-yl)prop-2-ynamide (2)

Eidgenössische Technische Hochschule Zürich
Laboratorium für Organische Chemie
Vladimir-Prelog-Weg 3 HCI E304 8093 Zürich Tel: 044/633 43 58
Mikroelementaranalyse

Name: Lanz Jan — Gruppe: OC ZHAW/ICBC
Labor: RT113 — Tel: 058/934 53 59

Substanz: ZHAWOC3212
Molekularformel: C21 H17 N3 O3 — Mr = 359,38 g/mol

Schmelzpunkt: Zers.>195°C — Sublimationspunkt:
gereinigt: chromat. — getrocknet: Vakuum/P2O5

Bestimmungen: C H N

Eingang: 19.03.14 — Ausgang: 19.03.14

M-157772 — Operator: SM

Berechnete Gewichtsanteile:
[C] 70,18% [H] 4,77% [N] 11,69% [O] 13,36%

Gefundene Gewichtsanteile:
Einwaage: 1,013mg — LECO TruSpec Micro
[C] 70,22% [H] 4,69% [N] 11,66% 19.03.14

4-{[5-(2-{[(3-methoxyphenyl)methyl]carbamoyl}eth-1-yn-1-yl)-2,4-dioxo-1,2,3,4-tetrahydropyrimidin-1-yl]methyl}benzoic acid (3)

Eidgenössische Technische Hochschule Zürich
Laboratorium für Organische Chemie
Vladimir-Prelog-Weg 3 HCI E304 8093 Zürich Tel: 044/633 43 58
Mikroelementaranalyse

Name: Lanz Jan — Gruppe: OC ZHAW/ICBC
Labor: RT113 — Tel: 058/934 53 59

Substanz: ZHAWOC3556
Molekularformel: C23 H19 N3 O6 — Mr = 433,42 g/mol

Schmelzpunkt: 207°C — Sublimationspunkt:
gereinigt: chromat./UK — getrocknet: 7d/110°C/Vakuum

Bestimmungen: C H N

Eingang: 09.04.14 — Ausgang: 09.04.14

M-157879 — Operator: PK

Berechnete Gewichtsanteile:
[C] 63,74% [H] 4,42% [N] 9,70% [O] 22,15%

Gefundene Gewichtsanteile:
Einwaage: 1,049mg — LECO TruSpec Micro
[C] 63,73% [H] 4,37% [N] 9,81% 09.04.14

4-({5-[2-({[3-(3-Carboxypropoxy)phenyl]methyl}carbamoyl)eth-1-yn-1-yl]-2,4-dioxo-1,2,3,4-tetrahydropyrimidin-1-yl}methyl)benzoic acid (4)

Eidgenössische Technische Hochschule Zürich
Laboratorium für Organische Chemie
Vladimir-Prelog-Weg 3 HCI E304 8093 Zürich Tel: 044/633 43 58
Mikroelementaranalyse

Name: Lanz Jan Gruppe: OC ZHAW/ICBC
Labor: RT113 Tel: 058/934 53 59

Substanz: ZHAWOC 4457
Molekularformel: C26 H23 N3 O8 Mr = 505,48 g/mol

Schmelzpunkt: Sublimationspunkt:
gereinigt: chromat. getrocknet: 110°C Vac P2O5

Bestimmungen: C H N

Eingang: 23.07.14 Ausgang: 23.07.14

M-158540 Operator: PK

Berechnete Gewichtsanteile:

[C]	61,78%	[H]	4,59%	[N]	8,31%	[O]	25,32%

Gefundene Gewichtsanteile:

Einwaage: 1,061mg LECO TruSpec Micro

**[C]**	**61,55%**	**[H]**	**4,54%**	**[N]**	**8,24%**	23.07.14

3-(1-Benzyl-2,4-dioxo-1,2,3,4-tetrahydropyrimidin-5-yl)-N-[(2-fluorophenyl)methyl]prop-2-ynamide (9a)

Eidgenössische Technische Hochschule Zürich
Laboratorium für Organische Chemie
Vladimir-Prelog-Weg 3 HCI E304 8093 Zürich Tel: 044/633 43 58
Mikroelementaranalyse

Name: Lanz Jan Gruppe: OC ZHAW/ICBC
Labor: RT113 Tel: 058/934 53 59

Substanz: ZHAWOC3376
Molekularformel: C21 H16 N3 O3 F Mr = 377,37 g/mol

Schmelzpunkt: 190°C zers. Sublimationspunkt:
gereinigt: chromat., UK getrocknet: HV

Bestimmungen: C H N

Eingang: 29.08.14 Ausgang: 29.08.14

M-158714 Operator: PK

Berechnete Gewichtsanteile:

[C]	66,84%	[H]	4,27%	[N]	11,13%	[O]	12,72%	[F]	5,03%

Gefundene Gewichtsanteile:

Einwaage: 0,980mg LECO TruSpec Micro

**[C]**	**66,67%**	**[H]**	**4,05%**	**[N]**	**11,02%**	29.08.14

3-(1-Benzyl-2,4-dioxo-1,2,3,4-tetrahydropyrimidin-5-yl)-N-[(4-fluorophenyl)methyl]prop-2-ynamide (9c)

Eidgenössische Technische Hochschule Zürich
Laboratorium für Organische Chemie
Vladimir-Prelog-Weg 3 HCI E304 8093 Zürich Tel: 044/633 43 58
Mikroelementaranalyse

Name: Lanz Jan | Gruppe: OC ZHAW/ICBC
Labor: RT113 | Tel: 058/934 53 50

Substanz:ZHAWOC3372
Molekularformel: C21 H16 N3 O3 F | Mr = 377,37 g/mol

Schmelzpunkt: 195°C zers. | Sublimationspunkt:
gereinigt: chromat., UK | getrocknet: 130°C Vac

Bestimmungen: C H N

Eingang: 10.09.14 | Ausgang: 10.09.14

M-158751 | Operator: PK

Berechnete Gewichtsanteile:

[C]	[H]	[N]	[O]	[F]
66,84%	4,27%	11,13%	12,72%	5,03%

Gefundene Gewichtsanteile:

Einwaage: 1,010mg | LECO TruSpec Micro

[C]	[H]	[N]	
**66,59%**	**4,05%**	**11,17%**	10.09.14

References

[1] *Molecular Operating Environment (MOE)*, Chemical Computing Group Inc., 1010 Sherbooke St. West, Suite #910, Montreal, QC, Canada, H3A 2R7, **2013**.

[2] A. R. Johnson, A. G. Pavlovsky, D. F. Ortwine, F. Prior, C.-F. Man, D. A. Bornemeier, C. A. Banotai, W. T. Mueller, P. McConnell, C. Yan, V. Baragi, C. Lesch, W. H. Roark, M. Wilson, K. Datta, R. Guzman, H.-K. Han, R. D. Dyer, *J. Biol. Chem.* **2007**, *282*, 27781–27791.

[3] H. M. Berman, J. Westbrook, Z. Feng, G. Gilliland, T. N. Bhat, H. Weissig, I. N. Shindaylov, P. E. Bourne, *Nucl. Acids Res.* **2000**, *28*, 235–242.

[4] E. F. Pettersen, T. D. Goddard, C. C. Huang, G. S. Couch, D. M. Greenblatt, E. C. Meng, T. E. Ferrin, *J. Comput. Chem.* **2004**, *25*, 1605–1612.

[5] N. G. Kundu, S. K. Dasgupta, *J. Chem. Soc., Perkin Trans 1* **1993**, 2657-2663.

[6] G. M. Coppola, R. E. Damon, *Synth. Commun.* **1993**, *23*, 2003-2010.
